# Endoscopic nasal delivery of engineered endothelial progenitor cell-derived exosomes improves angiogenesis and neurological deficits in rats with intracerebral hemorrhage

**DOI:** 10.1016/j.mtbio.2025.101652

**Published:** 2025-03-11

**Authors:** Gui Wan, Zhenwei Li, Lingui Gu, Ye Sun, Yuhe Wang, Yiqing Wang, Ruxu Geng, Yangyang Chen, Wenbin Ma, Xinjie Bao, Renzhi Wang

**Affiliations:** a Department of Neurosurgery, Peking Union Medical College Hospital, Chinese Academy of Medical Sciences and Peking Union Medical College, Beijing, 100730, China; b School of Medicine, The Chinese University of Hong Kong, Shenzhen, Guangdong, 518172, China; c State Key Laboratory of Common Mechanism Research for Major Diseases, Beijing, China; d Department of Obstetrics and Gynecology, The First Affiliated Hospital of Anhui Medical University, Anhui Medical College, Hefei, 230031, China

**Keywords:** Intracerebral hemorrhage, Endothelial progenitor cell exosomes, Blood-brain barrier, Brain microvascular endothelial cells, HSP90, Nasal endoscopic delivery

## Abstract

Intracerebral hemorrhage (ICH) remains a life-threatening condition due to its high mortality and limited treatment options. This study explores a novel therapeutic strategy using engineered exosomes derived from endothelial progenitor cells (EPC-EXOs) to improve ICH outcomes. EPC-EXOs were modified with a CD47-enriched red blood cell membrane via co-extrusion to enhance their anti-phagocytic properties, thereby reducing degradation by activated microglia after ICH. A minimally invasive endoscopic-guided delivery system was developed to facilitate the targeted intranasal administration of these engineered EPC-EXOs (m-Oe-EXOs), allowing direct entry into brain tissue. We confirmed m-Oe-EXOs’ high retention and effective distribution in the brain. Functional analysis demonstrated that EPC-EXOs significantly promoted the proliferation, migration, and angiogenesis of brain microvascular endothelial cells (BMECs), with proteomic analysis identifying HSP90 as a key protein activating the Akt pathway in BMECs. *In vivo*, m-Oe-EXOs demonstrated therapeutic efficacy by improving blood-brain barrier integrity, reducing hematoma volume, and enhancing neurological recovery in ICH rats. Collectively, our findings highlight the potential of minimally invasive, endoscopic-guided delivery of m-Oe-EXOs as an innovative approach for ICH treatment, providing new insights into targeted, exosomes-based regenerative therapies.

## Highlights

• EPC-EXOs enhance endothelial function post-ICH, promoting proliferation, migration, and angiogenesis for recovery.
• HSP90 in EPC-EXOs activates Akt signaling, mediating therapeutic effects in brain microvascular endothelial cells.
• Engineered EPC-EXOs with anti-phagocytic properties improve therapeutic efficacy in ICH treatment.
• A targeted, minimally invasive EPC-EXOs delivery system enhances therapeutic potential and clinical translation for ICH.

## Graphical abstract

**Figure:**
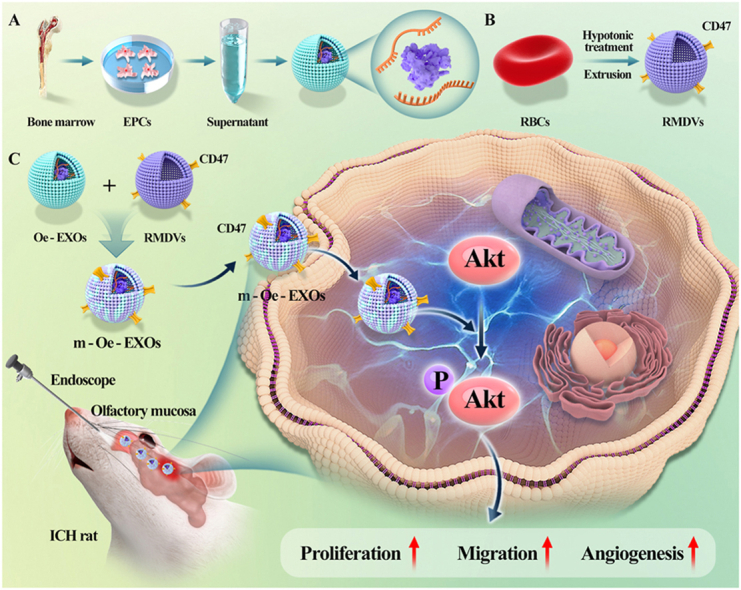

## 1 Introduction

Intracerebral hemorrhage (ICH) is a prevalent form of stroke, constituting approximately 15 % of all strokes and contributing to nearly 50 % of stroke-related mortality, resulting in around 2.8 million deaths globally each year [1]. Unlike ischemic strokes, ICH tends to occur in younger patients and is more likely to lead to fatal or permanently disabling outcomes [2]. The hematoma's mass effect, along with the release of hemoglobin and excess iron ions from ruptured erythrocytes, can cause severe damage to the brain parenchyma [3]. Traditional treatments for ICH, such as surgical hematoma evacuation and pharmacotherapy, come with substantial limitations [4,5]. Surgical interventions carry risks of infection, rebleeding, and tissue damage, while pharmacological treatments often face toxicity and adverse side effects, with challenges in dosing and targeting specificity [5]. Moreover, nearly all macromolecular drugs and over 98 % of small-molecule therapeutics cannot penetrate the blood-brain barrier (BBB) [5]. As a result, the development of novel therapeutic approaches for ICH has become a hot research topic.

Endothelial progenitor cells (EPCs), first identified by Professor Takayuki Asahara in 1997, serve as precursor cells for endothelial cells (ECs) [6,7]. Upon vascular injury, EPCs are directed by chemotactic factors to migrate specifically to the damaged sites, where they differentiate into ECs to aid in vascular repair [6,8]. Studies have demonstrated that elevated circulating levels of EPCs correlate with favorable prognosis in cases of ICH, and EPC transplantation has shown potential to improve certain neurological functions (NLFs) in rat models post-ICH [9,10]. However, clinical applications of EPCs remain limited due to concerns over immunogenicity risk, genetic instability, and ethical issues [11]. Increasing evidence suggests that exosome-based therapies, particularly those derived from stem cells, offer a safer and more feasible approach for clinical translation, thanks to their low immunogenicity and lack of ethical barriers [11]. Previous research has shown that exosomes derived from endothelial progenitor cells (EPC-EXOs) promote cutaneous wound healing by enhancing angiogenesis through Erk1/2 signaling [12]. Additionally, microvesicles from EPCs have been found to activate angiogenic programs in ECs via horizontal mRNA transfer [13]. However, the effects of EPC-EXOs on EC-mediated vascular repair through angiogenesis post-ICH have not yet been reported.

Microglia within the brain are the primary phagocytes in the central nervous system [14]. As members of the mononuclear phagocyte system (MPS), they are among the earliest non-neuronal cells to respond during the innate immune reaction following ICH [15]. Post-ICH, the phagocytic capacity of microglia is upregulated via pathways such as PPARγ/CD36 [15]. However, it remains unreported whether EPC-EXOs are phagocytosed by microglia after ICH. Meanwhile, signal regulatory protein alpha (SIRPα), a typical inhibitory immune receptor, is selectively expressed on myeloid cells, including microglia, macrophages, and granulocytes [16]. The interaction between CD47—an antiphagocytic marker—and SIRPα serves as a “do not eat me” signal and allows for evasion from MPS-mediated phagocytosis [17,18]. Therefore, if EPC-EXOs are phagocytosed by microglia following ICH, it is essential to explore the effects of EPC-EXOs on ICH prognosis, as well as how to engineer EPC-EXOs with CD47 enrichment to attempt to prevent their phagocytosis by microglia. Additionally, comparing the efficacy of EPC-EXOs before and after this engineering modification could provide valuable insights into their potential benefits in ICH treatment.

Stereotaxic injection (SI), a commonly used method of drug delivery in ICH research, can directly provide sufficient drug concentration at the brain lesions to effectively treat ICH [19,20]. However, while SI offers high targeting accuracy, it is also highly invasive, requiring strict operational conditions and expertise, which limits its widespread clinical application and hinders the translation of many ICH studies into practice [21,22]. To overcome the limitations of SI, an increasing number of researchers are focusing on developing novel central nervous system (CNS) drug delivery technologies or optimizing existing delivery strategies [23]. For instance, some researchers are exploring drug carriers as a means to enhance CNS drug delivery [23]. They have investigated the use of heavy-chain ferritin, iron oxide nanoparticles, gold nanoparticles, nanoliposomes, and virus-like nanoparticles as drug carriers to facilitate targeted delivery to the CNS [23]. In addition to carrier-based strategies, other researchers have explored nasal administration as a minimally invasive alternative approach to circumvent the limitations of SI [24]. The nasal mucosa is generally divided into three regions: the anterior vestibule, the respiratory mucosa, and the olfactory mucosa [25,26]. The anterior vestibule has a small surface area and sparse vascular distribution, making it a poor site for drug delivery [25,27]. The respiratory mucosa, which is extensively distributed over the nasal turbinates, has a larger surface area and a rich capillary network, facilitating drug absorption; however, its ciliated surface tends to propel drugs toward the pharynx [25,27]. In contrast, the olfactory mucosa is located deeper within the nasal cavity and has a relatively small area, making it the optimal site for delivering drugs to the brain [25,27,28]. In small experimental animals like rats, traditional drug delivery methods often involve blind insertion into the nasal cavity using pipettes or tubes, leading to a diffuse distribution of the drug within the nasal cavity [25,29,30]. Due to the fluid dynamics of the airway and the presence of cilia on the nasal mucosa, many drugs can inadvertently flow into the pharynx and gastrointestinal tract [25,27]. Therefore, for nasal-to-brain delivery, targeting drugs more precisely to the olfactory mucosa would undoubtedly enhance their entry into the brain. However, no methods for precise targeted delivery to the olfactory mucosa have been reported to date.

Therefore, in this study, we innovatively employed a minimally invasive endoscopic-guided approach for the visualized and targeted intranasal delivery of engineered EPC-EXOs. As a novel therapeutic strategy for ICH, we explored the underlying mechanisms by which EPC-EXOs exert therapeutic effects on ICH, specifically focusing on their impact on rat brain microvascular endothelial cells (RBMECs). Additionally, we engineered EPC-EXOs to possess anti-phagocytic properties and investigated the effects of endoscopic-guided delivery of these modified exosomes on ICH prognosis. Our study provides new insights into potential therapeutic strategies for ICH treatment.

## 2 Materials and methods

### 2.1 EPCs isolation and culture

EPCs were isolated from the bone marrow (BM) of the rats, and their culture and identification were performed as previously described [31,32]. Under sterile conditions, the femurs and tibias were excised from the rats. The BM was then flushed out using a 10 mL syringe, filtered, and resuspended in 4.5 mL of dilution buffer (Solarbio, R1017, China). Subsequently, the BM cell suspension was carefully layered onto the surface of the separation medium (Solarbio, P8980, China), ensuring a clear interface between the two phases. The sample was then centrifuged at 800×*g* for 30 min at room temperature. After centrifugation, four distinct layers were observed in the centrifuge tube, from top to bottom: a dilution buffer layer, a ring-shaped whitish layer of mononuclear cells, a transparent separation medium layer, and a red blood cell (RBC) layer. Using a pipette, the second layer (the mononuclear cell layer) was carefully aspirated and transferred to a clean 15 mL centrifuge tube. The mononuclear cells were washed by adding 10 mL of PBS, followed by centrifugation at 250×*g* for 10 min. This washing process was repeated three times to ensure thorough purification of the mononuclear cells. After the final wash, the collected mononuclear cells were cultured in Endothelial Cell Growth Basal Medium-2 (EBM-2, Lonza, CC-3156, Switzerland) supplemented with SingleQuots (Lonza, CC-4176, Switzerland), following the manufacturer's instructions.

### 2.2 Characterization of EPCs

Bright-field microscopy, fluorescent staining, immunocytochemistry, and flow cytometry analysis were utilized to characterize the EPCs. Initially, the cells were incubated with human DiI-acetylated low-density lipoprotein (DiI-Ac-LDL) for 4 h at 37 °C. Following this, the cells were fixed with 2 % paraformaldehyde and incubated for 1 h with FITC labeled Ulex europaeus agglutinin 1 (FITC-UEA-1) (Shanghai Maokang Biotechnology Co., Ltd, China). Observations were made using a fluorescent microscope (Olympus, Japan). For immunocytochemistry, the cells were fixed in 4 % paraformaldehyde, incubated overnight with primary antibodies, and then incubated for 1 h with secondary antibodies. After washing three times, the cells were examined under the fluorescence microscope. The CD34 antibody (1:100, Cat No. ab81289) was obtained from Abcam (Cambridge, Cambs., UK), while the vWF antibody (1:50, Cat No. 27186-1-AP) was sourced from Proteintech (Wuhan, China).

Lastly, flow cytometry was employed to assess the purity of the EPCs. The cells were digested and resuspended at a concentration of 1 × 106 cells/ml. EPCs were incubated at room temperature for 1 h with anti-CD31, anti-CD34, and anti-CD133 antibodies. The EPCs were then incubated in the dark at room temperature for 35 min with FITC–conjugated Goat Anti-Rabbit IgG(H + L) (Proteintech, China), washed three times with PBS, and resuspended in 200 μl PBS. Cell fluorescence intensity was measured using a FACS Calibur flow cytometer (BD, Franklin Lakes, USA). EPCs incubated with normal IgG served as negative controls. The CD31 antibody (1:100, Cat No. 15585) was purchased from Cell Signaling Technology (Boston, USA), the CD34 antibody (1:50, Cat No. ab81289) from Abcam (Cambridge, Cambs., UK), and the CD133 antibody (1:50, Cat No. 18470-1-AP) from Proteintech (Wuhan, China).

### 2.3 Isolation and identification of EPC-EXOs

EPC-EXOs were isolated from the supernatant of EPCs using the Total Exosomes Isolation Kit according to the manufacturer's instructions (Invitrogen, Asheville, NC, USA), as previously described [33]. Briefly, the culture medium was collected and centrifuged at 2000×*g* for 30 min to eliminate cells and debris. Exosomes were then extracted from the cell-free medium using the Total Exosomes Isolation Kit, resuspended in PBS, and stored at −80 °C. The protein concentration was quantified using a BCA protein assay kit (Beyotime Biotechnology, Shanghai, China). The morphology of the EPC-EXOs was observed using a transmission electron microscope (TEM, Hitachi, Tokyo, Japan). The size and zeta potential of the EPC-EXOs were evaluated using the ZETASIZER PRO (Malvern Panalytical, UK). Exosomal positive markers CD9, CD63, and TSG101, as well as the negative marker Calnexin, were analyzed by Western Blot (WB).

### 2.4 Intracellular uptake of exosomes

Exosomes were labeled with the cell membrane red fluorescent probe DiI (Beyotime, C1036, Shanghai, China) following the manufacturer's instructions. RBMECs or rat microglia (RM) were cultured in confocal dishes and incubated with DiI-labeled exosomes for 24 h. Phalloidin and DAPI (Solarbio, Beijing, China) were used to stain the cytoskeleton and nucleus prior to image acquisition. Images were captured using a confocal microscope (Nikon, Tokyo, Japan).

### 2.5 *In vitro* tube formation assay

RBMECs (1 × 10^4^) were first plated in 96-well plates coated with Matrigel Basement Membrane Matrix (BD Biosciences, USA). After incubating for 6 h at 37 °C, microscopy was employed to assess the tube formation capability of the RBMECs. The total capillary length and the number of branch points of the tubes were measured using the “Angiogenesis Analyzer" plugin available in ImageJ [34,35].

### 2.6 RBMEC migration assay

The migration ability of RBMECs was evaluated using a 24-well Transwell Chamber with an 8.0 μm pore size (Corning, USA). A total of 2 × 104 RBMECs suspended in serum-free medium were placed in the upper chamber, while the lower chamber contained complete medium with 20 % fetal bovine serum. After incubating the plate for 16 h at 37 °C in 5 % CO_2_, the cells were fixed with 4 % paraformaldehyde for 15 min and stained with 0.05 % crystal violet in phosphate-buffered saline (PBS) for another 15 min. Cells on the upper side of the filters were then removed using cotton-tipped swabs, and the filters were washed three times with PBS. Finally, a microscope was used to observe the cells on the underside of the filters [36].

### 2.7 EdU staining

RBMECs were seeded in 96-well plates at a density of 7000 cells per well and incubated for 24 h. Following this, the cells were treated with EdU for 2 h to assess proliferation rates using the BeyoClick™ EdU Cell Proliferation Kit with Alexa Fluor 488 (Beyotime, China). The results were analyzed using Image Pro Plus 6.0.

### 2.8 Label-free quantitative proteomics

Label-free quantitative proteomics was conducted by Wayen Biotechnologies (Shanghai, China). The workflow is detailed as follows.

#### 2.8.1 Exosome protein extraction

Exosome samples were precipitated by adding six volumes of 100 % acetone, followed by incubation at −20 °C for 2 h. The precipitate was centrifuged at 13,000 rpm for 15 min at 4 °C, and the supernatant was discarded. The EasyPapt Ex Kit (OSFP0001) was used for protein extraction. The precipitate was resuspended with 20 μL of Reagent A from the kit and processed for protein quantification using a BCA assay. Based on the quantification results, 20 μg of protein from each sample was used for subsequent proteolysis.

#### 2.8.2 Protein digestion

The protein solution was adjusted to a total volume of 20 μL with Reagent A and vortexed. The mixture was heated at 95 °C for 5 min and allowed to cool to room temperature. Subsequently, 15 μL of Reagent B from the kit was added and incubated at 37 °C for 2 h. The digestion reaction was terminated by adding 4 μL of Reagent C from the kit, leading to precipitation, which was centrifuged at 20,000 g for 1 min.

#### 2.8.3 Peptide desalting

Peptides were desalted using desalting columns (Thermo Scientific™, 84,850) following these steps: 1. Add 100 μL of methanol to the column and centrifuge at 700 g for 1 min; 2. Add 100 μL of Condition Buffer, centrifuge at 700 g for 1 min; 3. Add 100 μL of Wash Buffer, centrifuge at 700 g for 1 min; 4. Load the entire sample onto the column and centrifuge at 700 g for 1 min, repeating the step once; 5. Add 100 μL of Wash Buffer and centrifuge at 700 g for 1 min, repeating the step once; 6. Elute peptides twice using 50 μL of Elution Buffer, centrifuging at 700 g for 1 min each time. The final volume was concentrated using a vacuum concentrator.

#### 2.8.4 Liquid chromatography-mass spectrometry (LC-MS) analysis

The dried peptides were reconstituted in mobile phase A (100 % H_2_O, 0.1 % formaldehyde) and centrifuged at 20,000 g for 10 min. The supernatant was injected into a Bruker NanoElute system for separation. The peptides were first enriched and desalted on a trap column and then separated on a self-packed C18 analytical column (75 μm inner diameter, 1.8 μm particle size, 25 cm length) at a flow rate of 300 nL/min. The gradient was as follows: 1. 0 min: 2 % mobile phase B (100 % ACN, 0.1 % formaldehyde); 2. 0–45 min: linear increase of mobile phase B from 2 % to 22 %; 3. 45–50 min: increase of mobile phase B from 22 % to 35 %; 4. 50–55 min: increase of mobile phase B from 35 % to 80 %; 5. 55–60 min: 80 % mobile phase B. The separated peptides were ionized using a CaptiveSpray source and analyzed on a timsTOF Pro2 mass spectrometer in DIA (Data Dependent Acquisition) mode. Key parameters included: 1. Ion source voltage: 1.5 kV; 2. Ion mobility range: 0.76–1.29 V s/cm^2^; 3. MS1 range: 452–1152 *m*/*z*; 4. Signal intensity threshold: 2500; 5. DIA windows: 4 steps, 7 windows per step, 56 total windows; 6. Collision-induced dissociation (CID) energy: 20–59 eV; 7. Cycle time: 1.59 s.

#### 2.8.5 Instrumentation

1. Refrigerated centrifuge: Fresco 17, Thermo Scientific, USA; 2. Room temperature centrifuge: Pico21, Thermo Scientific, USA; 3. Vortex mixer: Vortex 5, QILINBER, China; 4. Vacuum concentrator: SPD111V, Thermo Scientific, USA; 5. UPLC system: NanoElute, Bruker, Germany; 6. Mass spectrometer: timsTOF Pro2, Bruker, Germany; 7. Microplate reader: Infinite® F50, TECAN, USA.

#### 2.8.6 Reagents and consumables

1. Trifluoroacetic acid: T6508-100 mL, Sigma, USA; 2. Acetonitrile: A998-4, Thermo Scientific, USA; 3. Acetone: A929-4, Thermo Scientific, USA; 4. Acetic acid: 10000218, Sinopharm, China; 5. BCA assay kit (micro): P0009, Beyotime, China; 6. EasyPapt Kit: OSFP0001, EasyPapt, China.

#### 2.8.7 KEGG pathway enrichment analysis

Subsequently, KEGG pathway enrichment analysis was performed on the raw data (Supplementary File 4) to identify key pathways and molecules involved in the functions of the exosomes.

### 2.9 Western blotting

Equal amounts of total protein (20–40 μg) were subjected to sodium dodecyl sulfate-polyacrylamide gel electrophoresis (SDS-PAGE) and transferred to polyvinylidene fluoride (PVDF) membranes. The membranes were blocked with 5 % w/v bovine serum albumin for 1 h at room temperature, followed by overnight incubation with primary antibodies at 4 °C. Afterward, the membranes were incubated with secondary antibodies for 2 h and visualized using X-ray film and the BioSpectrum Imaging System (UVP, CA, USA). The references for all antibodies used in this study are listed in Table S3 (Supplementary File 1). And the uncut membranes of WB are included in the Supplementary File 3.

### 2.10 Lentiviral infection

The lentiviral vectors used for overexpression of rat HSP90 cDNA (Oe) and the empty vector (Ev) were constructed by Shanghai Genechem Co., Ltd. Additionally, lentiviral vectors carrying three distinct HSP90 short hairpin RNAs (shRNAs) and a negative control (NC) were also obtained from the same company. The target sequences are provided in Table S1 (Supplementary File 1). Lentiviral infection was performed on EPCs cultured to 30 % confluence in 12-well plates, using lentiviral particles at a multiplicity of infection (MOI) of 40. The efficiency of the infection was assessed by quantitative real-time polymerase chain reaction (qRT-PCR) and WB analysis.

### 2.11 Manufacture of CD47-enriched EPC-EXOs

The production of CD47-enriched EPC-EXOs utilized the protocols outlined by Ennio Tasciotti et al. [37]. Briefly, following the isolation of RBCs from blood, CD47-enriched EPC-EXOs were synthesized using a two-step process: (i) RBC membrane-derived vesicles (RMDVs) were fabricated through hypotonic treatment of RBCs, followed by extrusion, and (ii) exosomes were coated with CD47 via mechanical co-extrusion to fuse them together.

### 2.12 *In vitro* cellular uptake assay

Flow cytometry was employed to assess the *in vitro* cellular uptake rate. In brief, RBMECs, RM, HT22 neurons or CTX-TNA2 astrocytes were seeded into 6-well culture plates for 12 h. After the cells had adhered, the culture medium was then replaced with fresh medium (2 mL per well). DiI-labeled Oe-EXOs (160 μg per well, corresponding to a concentration of 80 μg/mL) or DiI-labeled Oe-EXOs modified with RMDVs at protein weight ratios of 1:1, 1:2, and 1:3 (Oe-EXOs:RMDVs, 160 μg per well, corresponding to a concentration of 80 μg/mL) were added to the medium. The cells were incubated for an additional 24 h. Following incubation, the cells were washed twice with PBS and then harvested. The cellular uptake of these exosomes was evaluated using an LSRFortessa Flow Cytometer (BD, USA) according to the manufacturer's instructions. Data analysis was performed using FlowJo software (version 10.8.1, BD Biosciences).

### 2.13 ICH model induction and delivery of exosomes

The collagenase-induced ICH animal model was employed in this study [38]. All rats were divided randomly into four groups as follows: exosomes SI-delivered group, PBS SI-delivered group, intranasal exosomes delivered group and intranasal PBS delivered group. Briefly, each rat was intubated and maintained under anesthesia using 3 % isoflurane mixed with oxygen in a 7:3 ratio, delivered via a rodent ventilator (Harvard Apparatus, Holliston, MA, USA). ICH was induced by sequentially injecting 1 μL of saline and 0.2 U of collagenase (type IV, Sigma) into the basal ganglion at the following stereotaxic coordinates: 0.26 mm anterior, 3.0 mm left lateral, and 6.0 mm deep.

One hour after collagenase injection, 100 μL of m-Oe-EXOs (at a concentration of 80 μg/mL) or PBS were delivered via SI into the basal ganglion in the first two SI groups. Then, the burr hole was sealed with bone wax, the skin was sutured, and the area was sterilized. In the last two groups treated by intranasal pathway, rats were placed in a supine position and first administered 100 U of hyaluronidase into the nasal cavity. After 30 min, under endoscopic guidance, a total of 100 μL of m-Oe-EXOs (at a concentration of 80 μg/mL) or PBS (50 μL per nostril) were targeted and injected into the olfactory mucosa (Supplementary File 2). After the injection, rats maintained the supine position for 10 min with a pillow placed under the head [39]. If the rats exhibited a significant cough reflex, the position was changed to prone. Throughout the procedure, body temperature was maintained at 37 °C using an electric blanket.

### 2.14 Study of brain distribution of exosomes

For the study of the brain distribution of exosomes, the exosomes were labeled with DiI. After exosome administration, fluorescence intensity of DiI in the brain was monitored at 3, 6, 9, 12, and 24 h using a small animal live imaging system.

### 2.15 Evans Blue Leakage Assay and hemoglobin detection

On the third day post-ICH induction, a 4 % Evans blue solution (4 mL/kg) was administered via tail vein injection. Four hours later, the rats were deeply anesthetized. Each rat was perfused with 200–300 mL of cold PBS through the heart, and the cerebral hemispheres on the hemorrhagic side were harvested. One milliliter of ultrapure water was added to each hemisphere, followed by centrifugation at 12,000 g for 30 min at 4 °C. The resulting supernatant was then analyzed for both hemoglobin content and the amount of Evans blue leakage.

### 2.16 Modified Garcia Test

Six distinct aspects were evaluated, including voluntary movement, posture symmetry, forelimb extension function, climbing ability and grip strength, bilateral body sensation, and bilateral whisker touch response. Each test was scored on a scale of either 0–3 or 1–3, with a maximum possible score of 18. A higher score indicated better NLF.

### 2.17 Corner Test

Rats were placed at the entrance of a 30° angle formed by two boards for 10 trials, and the percentage of rightward turns was calculated. A higher percentage of rightward turns indicated more severe neurological deficits.

### 2.18 Statistical analysis

Data are presented as mean ± standard deviation (SD). The Shapiro-Wilk test, performed using SPSS software, was employed to assess the normality of the data. Datasets meeting the assumption of normal distribution (p-value >0.05) were subjected to further statistical analysis. Subsequent statistical analyses were conducted using GraphPad Prism 8.0, with either one-way ANOVA or Student's t-test applied, as appropriate. A p-value of <0.05 was considered statistically significant. All statistical graphs were generated using GraphPad Prism 8.0.

## 3 Results

### 3.1 Characterization of EPCs and EPC-EXOs

**Fig. 1:**
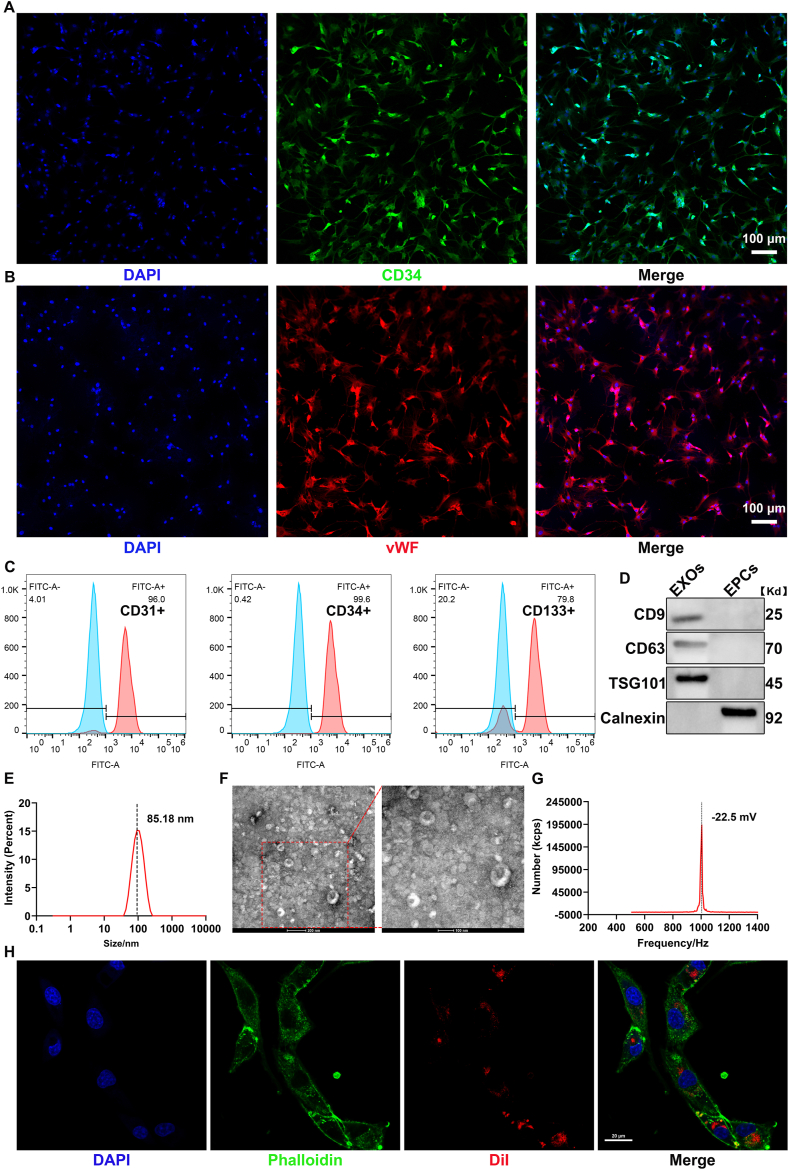
Characterization of EPCs and EPC-EXOs. (A, B) Immunocytochemistry showed that EPCs were positive for CD34 and vWF staining. Scale bar: 100 μm. (C) The flow cytometry analysis showed that 96 % of the harvested cells were positive for CD31, 99.6 % were positive for CD34 and 79.8 % were positive for CD133. (D) Western blot analysis of EPC-EXOs markers including CD9, CD63, TSG101 and Calnexin. (E–G) Show the diameter, morphology and zeta potential of EPC-EXOs respectively. (H) Confocal images of RBMECs incubated with 20 μg DiI-labeled EPC-EXOs for 24 h. Scale bar: 20 μm.

Primary EPCs were successfully isolated from rat BM cell suspensions and identified based on their morphology, dual uptake properties, and surface markers. Microscopic analysis revealed that the EPCs exhibited a typical “paving-stone” morphology (Supplementary File 1: Fig. S1). Additionally, these cells demonstrated the ability to uptake both DiI-Ac-LDL and FITC-UEA-1, which is characteristic of EPCs (Supplementary File 1: Fig. S2). To further confirm their identity, four surface markers highly expressed on EPCs—CD34, vWF, CD31, and CD133—were utilized. Both immunocytochemical staining and flow cytometry analysis confirmed that high-purity EPCs were successfully isolated for use in the present study (Fig. 1A–C). These findings collectively verify the successful isolation of EPCs.

Exosomes were isolated from the culture supernatants of EPCs. WB analysis revealed the expression of typical exosomal markers, including CD9, CD63, and TSG101, while Calnexin was not detected (Fig. 1D). As shown in Fig. 1E–G, the measured diameter and zeta potential of the exosomes were about 85.18 nm and −22.5 mV, respectively, which fall within the expected range for exosomes. Additionally, TEM analysis confirmed the presence of characteristic exosomal structures (Fig. 1F). These results collectively confirm the successful isolation of EPC-EXOs.

### 3.2 EPC-EXOs are internalized by RBMECs and promote angiogenesis, migration, and proliferation in hemin-treated RBMECs

**Fig. 2:**
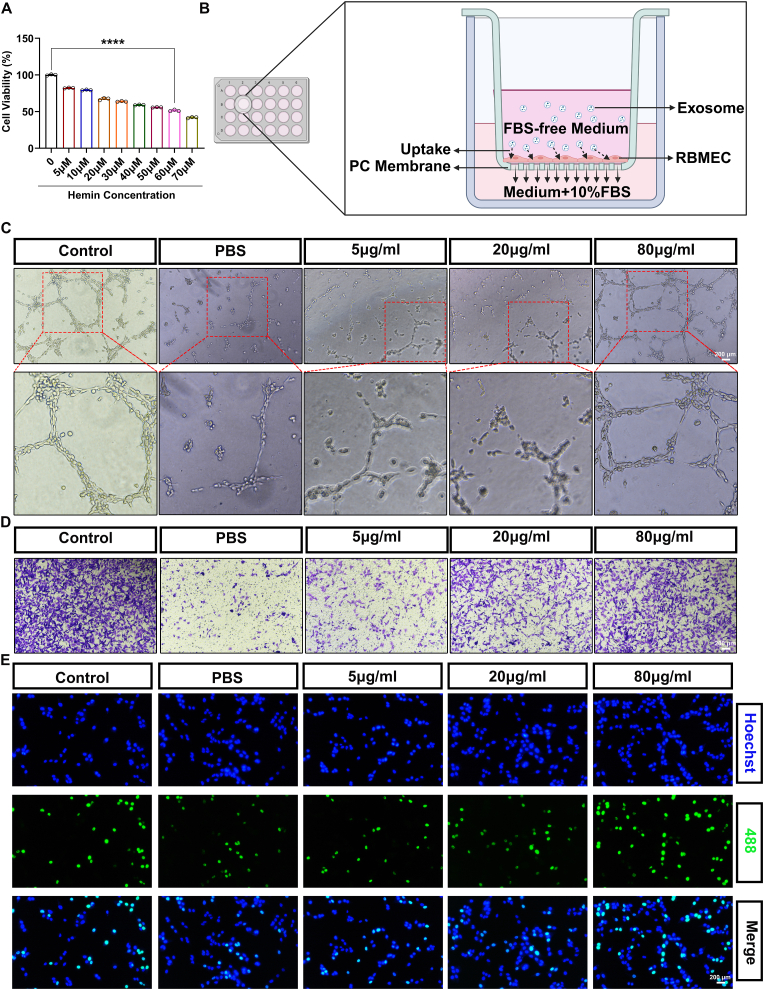
EPC-EXOs could promote hemin-treated RBMECs function. (A) Cell viability determined by the CCK8 analysis. (B) Schematic diagram of transwell migration assay. (C) Representative images of tube formation of RBMECs. Scale bar: 200 μm. (D) Representative images of migrating RBMECs in transwell assay. The migrating RBMECs stained purple by crystal violet. Scale bar: 200 μm. (E) Representative images of proliferative RBMECs in EdU staining. The proliferative cells were stained with green color, and their cellular nucleis were stained with blue color. Scale bar: 200 μm. (ns indicates no significant, ∗p < 0.05, ∗∗p < 0.01, ∗∗∗p < 0.001, ∗∗∗∗p < 0.0001). (For interpretation of the references to color in this figure legend, the reader is referred to the Web version of this article.)

After ICH, blood extravasates into the brain parenchyma, accumulating locally to form a hematoma. The hematoma contains a high concentration of hemoglobin, which plays a critical role in secondary injuries to neurons and vascular endothelial cells [3]. Hemoglobin within the hematoma undergoes a sequential degradation process into oxyhemoglobin, deoxyhemoglobin, and ultimately heme and ferrous ions. Studies have shown that heme can accumulate intracellularly, causing peroxidative damage to membrane structures, impairing mitochondrial function, and disrupting cellular energy metabolism. Membrane damage increases cellular permeability, leading to cell swelling, impaired material exchange, and altered membrane potential. Thus, heme serves as a key factor in simulating the ICH microenvironment *in vitro* to explore post-ICH cellular functional changes [40]. To replicate the excessive release of heme following RBC rupture in ICH, we first employed hemin treatment. RBMECs were exposed to various concentrations of hemin (0, 5, 10, 20, 30, 40, 50, 60, and 70 μmol/L) for 24 h. The CCK-8 assay revealed a reduction in cell viability at as low as 5 μmol/L hemin, with a progressive decline in viability as the hemin concentration increased. At 60 μmol/L, cell viability decreased by approximately 50 %. Collectively, these findings indicate that hemin treatment of RBMECs effectively models post-ICH injury responses in RBMECs. Consequently, 60 μmol/L hemin was selected for subsequent experiments (Fig. 2A). Next, we evaluated the internalization of EPC-EXOs by RBMECs. The cells were incubated with 20 μg of DiI-labeled EPC-EXOs for 24 h. The red fluorescence observed in the cytoplasm of RBMECs clearly indicated that EPC-EXOs were efficiently internalized by the cells (Fig. 1H).

To investigate the functional impact of EPC-EXOs on hemin-treated RBMECs, different concentrations of EPC-EXOs ranging from 5 to 80 μg/mL were applied, taking into account both potential side effects and the cost considerations of high-concentration treatments [41]. As shown in Fig. 2C and Supplementary File 1: Figs. S3A and B, the analysis of tube formation length and branching demonstrated no significant difference between 5 μg/mL and 20 μg/mL EPC-EXOs. However, overall, the mean length and branch number of tube-like structures were higher in the 20 μg/mL group compared to the 5 μg/mL group. EPC-EXOs promoted tube formation in a dose-dependent manner, with 80 μg/mL yielding the most substantial enhancement. Furthermore, a transwell migration assay was used to assess the effect of EPC-EXOs on the migratory capacity of RBMECs (Fig. 2B). RBMECs were loaded onto the upper side of the transwell membrane and after 24 h, the cells that had migrated to the bottom of the membrane were fixed, stained, and counted. The highest migration rate was observed in the 80 μg/mL EPC-EXOs-treated group. The migration rates in the other EPC-EXOs-treated groups decreased in the following order: 20 μg/mL > 5 μg/mL > 0 μg/mL (PBS). This result confirmed that EPC-EXOs exert a dose-dependent pro-migratory effect on RBMECs (Fig. 2D and Supplementary File 1: Fig. S3C). Finally, EdU staining was employed to assess the effect of EPC-EXOs on the proliferation of RBMECs. As shown in Fig. 2E and Supplementary File 1: Fig. S3D, the proliferation rate of RBMECs increased gradually with the concentration of EPC-EXOs, reaching the highest level in the 80 μg/mL group. This dose-dependent effect on proliferation was similar with the pro-migratory effect observed. In conclusion, these findings demonstrate that EPC-EXOs can be internalized by RBMECs and enhance their angiogenic potential, migration, and proliferation in response to hemin-induced injury. Moreover, the effects of EPC-EXOs were found to be dose-dependent.

### 3.3 Exosomal HSP90 is the key protein in EPC-EXOs that regulates the function of RBMECs identified by proteomic analysis of EPC-EXOs

**Fig. 3:**
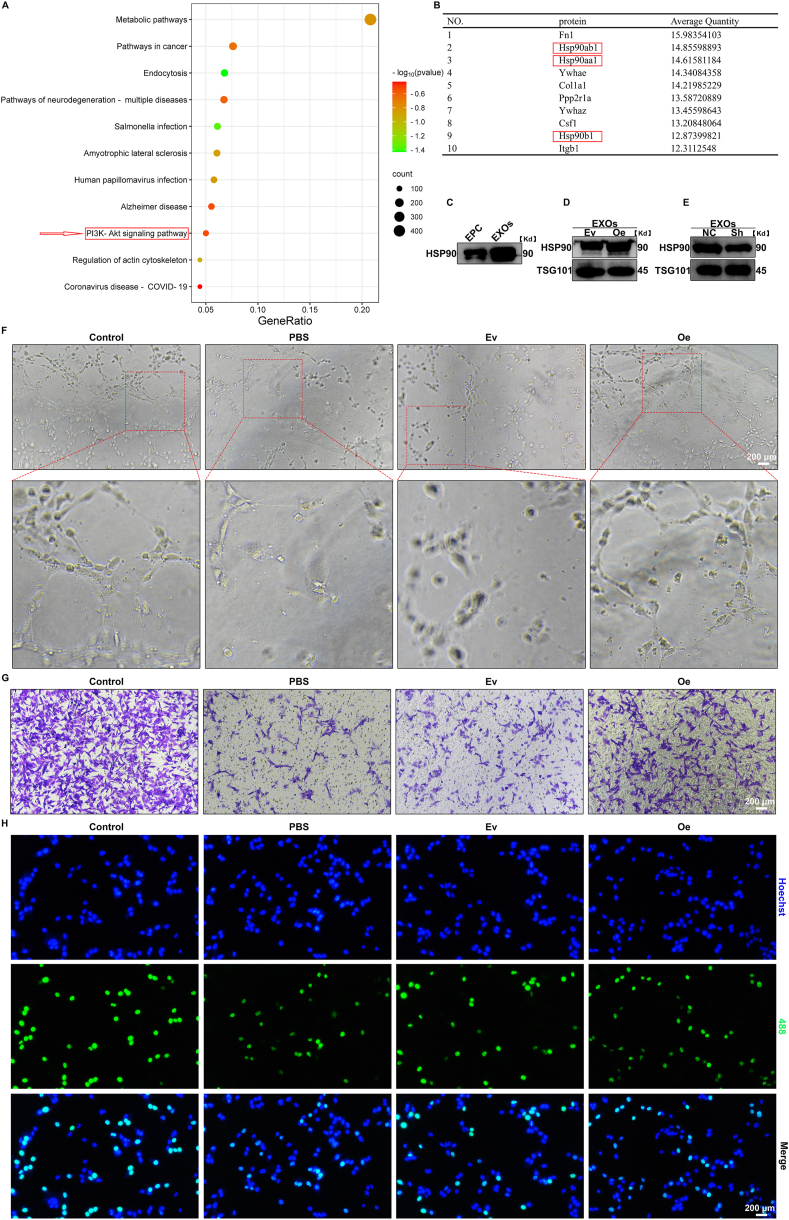
Exosomal HSP90 enhanced the function of hemin-treated RBMECs. (A) The KEGG pathway enrichment analysis was carried out on the 3972 proteins identified by proteomic analysis. (B) Illustration of the top ten high-abundance proteins enriched in the PI3K-Akt signaling pathway. (C) Western blot analysis of HSP90 levels in EPCs and EPC-EXOs. (D) The protein expression level of HSP90 in Ev-EXOs and Oe-EXOs was analyzed by western blotting assay. (E) The protein expression level of HSP90 in NC-EXOs and Sh-EXOs was analyzed by western blotting assay. (F) Representative images of tube formation of RBMECs. Scale bar: 200 μm. (G) Representative images of migrating RBMECs in transwell assay. The migrating RBMECs stained purple by crystal violet. Scale bar: 200 μm. (H) Representative images of proliferative RBMECs in EdU staining. The proliferative cells were stained with green color, and their cellular nucleis were stained with blue color. Scale bar: 200 μm. (For interpretation of the references to color in this figure legend, the reader is referred to the Web version of this article.)

To investigate the mechanism by which exosomes regulate the function of hemin-treated RBMECs, we performed a proteomic analysis of EPC-EXOs. A total of 3972 proteins were identified, and KEGG pathway enrichment analysis was conducted to explore key molecular pathways involved. Fig. 3A shows the top 10 ranked pathways by gene number. Among them, the PI3K-Akt signaling pathway stands out as a critical intracellular signal transduction pathway that responds to extracellular signals and promotes metabolism, proliferation, cell survival, migration, growth, and angiogenesis [42,43]. Based on these findings, we hypothesized that the recovery of RBMEC function in the ICH environment promoted by EPC-EXOs might be associated with the PI3K-Akt signaling pathway. Next, we sorted the 104 proteins enriched in the PI3K-Akt signaling pathway by their abundance. Fig. 3B shows the top 10 proteins ranked by abundance, among which three were identified as HSP90 proteins. As a heat shock protein, HSP90 is known for its role in maintaining cellular homeostasis and protecting the body under stress [44]. Therefore, we hypothesized that exosomal HSP90 is the key protein responsible for modulating the function of RBMECs by EPC-EXOs. To verify this, WB analysis was firstly performed to confirm the high abundance of HSP90 in EPC-EXOs (Fig. 3C). For WB analysis, both groups were loaded with 30 μg of protein per lane. Subsequently, to explore the relationship between exosomal HSP90 and the functionality of hemin-treated RBMECs, we overexpressed and knocked down HSP90 in EPCs using lentivirus to obtain EPC-EXOs with either overexpressed (Oe-EXOs) or knocked down HSP90 (Sh-EXOs). As shown in Supplementary File 1: Figs. S5A and B, qRT-PCR confirmed the successful overexpression and knockdown of HSP90 in EPCs. Since the knockdown efficiency of Sh2 was the highest, all subsequent experiments used Sh2 for HSP90 knockdown. Exosomes were successfully isolated from EPCs with HSP90 overexpression or knockdown (Supplementary File 1: Fig. S6), and WB revealed significant changes in HSP90 protein levels in the exosomes derived from these EPCs (Fig. 3D and E). To assess the impact of exosomal HSP90 on the function of hemin-treated RBMECs, we performed *in vitro* tube formation assays, RBMEC migration assays, and EdU staining. As shown in Fig. 3F–H, Supplementary File 1: Fig. S4 and Fig. S5C-J, compared to exosomes derived from the EV group (Ev-EXOs), Oe-EXOs significantly enhanced the functional recovery of hemin-treated RBMECs. In contrast, Sh-EXOs showed a weakened effect on the functional recovery of hemin-treated RBMECs compared to those derived from the NC group (NC-EXOs). Collectively, these findings indicate that exosomal HSP90 plays a pivotal role in enhancing the function of hemin-treated RBMECs, and it is a key protein in EPC-EXOs that regulates RBMECs’ functionality.

### 3.4 Exosomal HSP90 enhances the function of hemin-treated RBMECs by activating the Akt Pathway in RBMECs

**Fig. 4:**
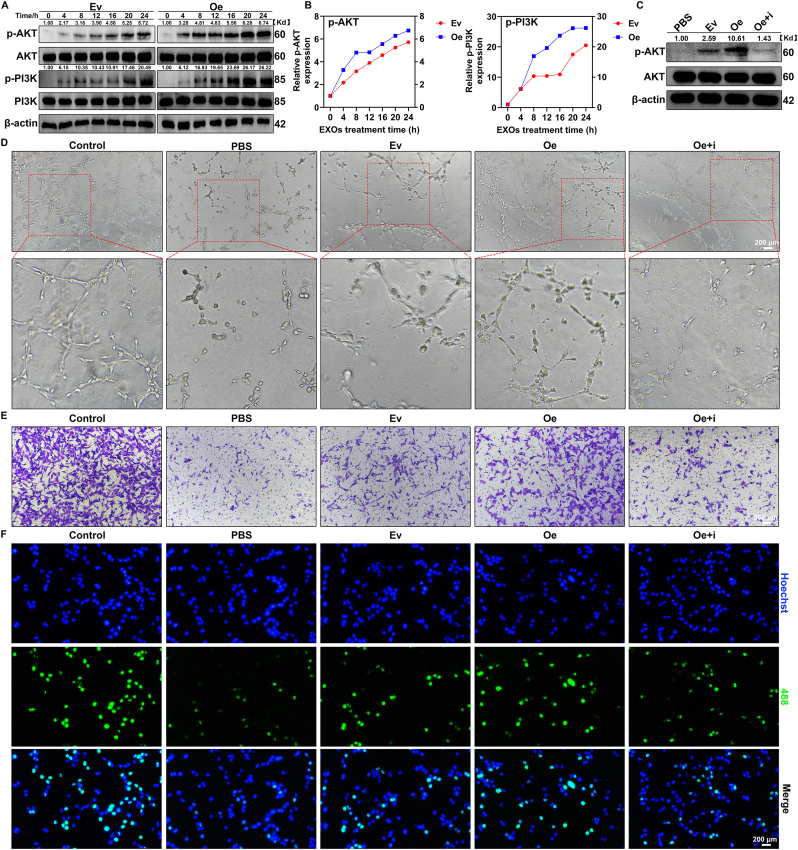
Exosomal HSP90 enhanced the function of hemin-treated RBMECs by activating the Akt Pathway in RBMECs. (A) Western blot analysis of the activation of Akt pathways in RBMECs after treated with Ev-EXOs and Oe-EXOs at different times. (B) Data presentation of Fig. 4A. (C) Western blot analysis of the activation of Akt pathways in RBMECs after using MK-2206. (D) Representative images of tube formation of RBMECs. Scale bar: 200 μm. (E) Representative images of migrating RBMECs in transwell assay. The migrating RBMECs stained purple by crystal violet. Scale bar: 200 μm. (F) Representative images of proliferative RBMECs in EdU staining. The proliferative cells were stained with green color, and their cellular nucleis were stained with blue color. Scale bar: 200 μm. (For interpretation of the references to color in this figure legend, the reader is referred to the Web version of this article.)

Next, we further investigated the intracellular signaling pathways activated in RBMECs during the incubation with EPC-EXOs. We initially focused on the Akt pathway, which was identified as an important signaling pathway through KEGG enrichment analysis. As shown in Fig. 4A, B and Supplementary File 1: Figs. S7A–C, RBMECs were treated with exosomes for different time (0, 4, 8, 12, 16, 20, and 24 h) after being exposed to Hemin, and proteins from the cells were collected for WB analysis. The results revealed that Ev-EXOs and NC-EXOs were able to induce the phosphorylation of PI3K and Akt, with phosphorylation levels increasing over time. When HSP90 was overexpressed in EPC-EXOs, the phosphorylation of PI3K and Akt at each time point was significantly higher compared to the EV group. Conversely, when HSP90 was knocked down in EPC-EXOs, the phosphorylation levels of PI3K and Akt were lower at each time point compared to the NC group. These results provide strong evidence that exosomal HSP90 directly participates in activating the Akt pathway in hemin-treated RBMECs. Therefore, we hypothesized that exosomal HSP90 enhances the function of hemin-treated RBMECs by activating the Akt pathway. To test this hypothesis, we utilized MK-2206 (an Akt inhibitor) and SC79 (an Akt activator) to further investigate the phosphorylation levels of Akt in RBMECs and assess their functions. As shown in Fig. 4C, hemin-treated RBMECs were incubated with PBS, Ev-EXOs, or Oe-EXOs for 24 h. In last group, RBMECs were pre-treated with MK-2206 before being incubated with Oe-EXOs. The results showed that, compared to PBS treatment, Ev-EXOs induced phosphorylation of Akt in RBMECs. Correspondingly, *in vitro* tube formation assays, RBMEC migration assays, and EdU staining demonstrated enhanced functional activity in RBMECs (Fig. 4D–F and Supplementary File 1: Fig. S9 A, B, E, G). Oe-EXOs induced a further increase in Akt phosphorylation and significantly enhanced RBMEC function compared to the EV group. Interestingly, when RBMECs were pre-treated with MK-2206, the Akt phosphorylation induced by Oe-EXOs was inhibited, and RBMEC function was also diminished. A similar pattern was observed in experiments involving PBS, NC-EXOs, Sh-EXOs, and SC79 (Supplementary File 1: Fig. S7D, Fig. S8 and Fig. S9 C, D, F, H). Collectively, these data strongly suggest that exosomal HSP90 enhances the function of hemin-treated RBMECs by activating the Akt pathway in RBMECs.

### 3.5 Engineering EPC-EXOs with RBC membranes to prevent phagocytosis by microglia

**Fig. 5:**
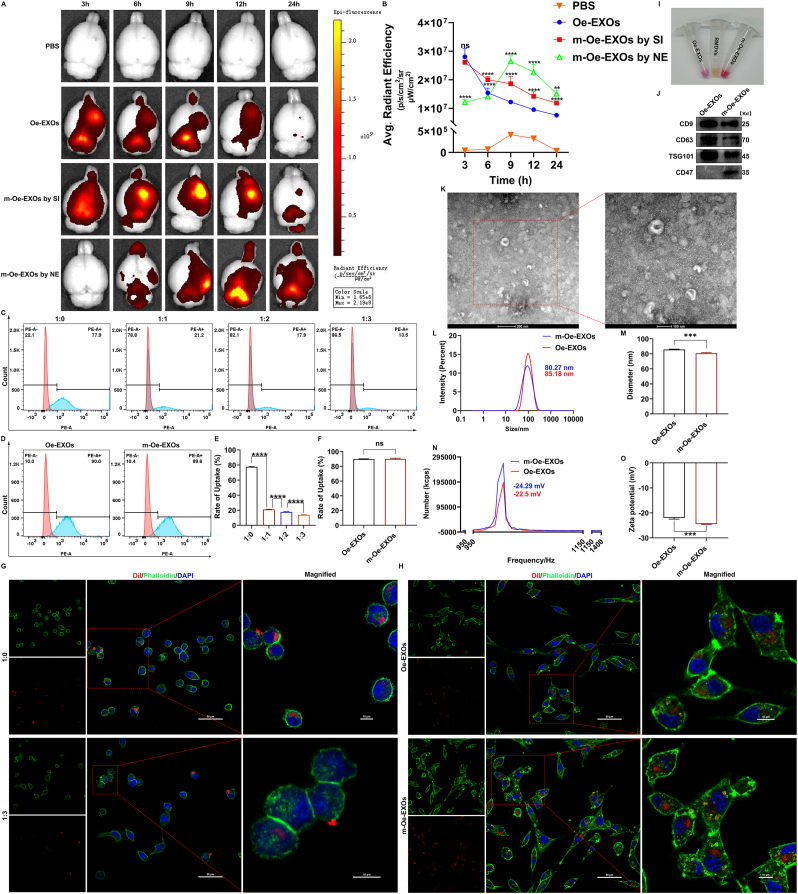
Engineering EPC-EXOs with RBC membranes effectively prevented phagocytosis by microglia. (A) Changes of DiI fluorescence intensity in brain over time in ICH rat. (B) Statistical analysis of A. N = 6. (C) Rate of uptake of microglia detected by flow cytometry analysis. (D) Rate of uptake of RBMECs detected by flow cytometry analysis. (E, F) Statistical analysis of C and D respectively. N = 3. (G, H) Confocal images of microglia and RBMEC respectively. Scale bar: 50 and 10 μm. (I) Three tubes containing DiI-labeled Oe-EXOs, DiO-labeled RMDVs and the co-extrusion product at a 1:3 ratio respectively. (J) Western blot analysis of typical exosomal markers and CD47 in Oe-EXOs and the co-extrusion product at a 1:3 ratio. (K) The morphology of the co-extrusion product at a 1:3 ratio (L–O) Comparison of diameter and zeta potential of Oe-EXOs and m-Oe-EXOs. N = 3. (ns indicates no significant, ∗p < 0.05, ∗∗p < 0.01, ∗∗∗p < 0.001, ∗∗∗∗p < 0.0001). NE: nasal endoscope.

Given the capability of exosomal HSP90 to enhance the function of hemin-treated RBMECs, we explored the application of Oe-EXOs in treating rats with ICH. One hour after collagenase injection, 100 μL of high-concentration DiI-labeled Oe-EXOs were stereotaxically injected into the brain of ICH rats. After injection, rats were sacrificed at 3, 6, 9, 12, and 24 h to collect whole brain tissue (including the olfactory bulb, cerebrum, and cerebellum). Small animal live imaging showed that Oe-EXOs were rapidly degraded in the brain, with approximately two-thirds degraded within 24 h (Fig. 5A and B). Considering the enhanced phagocytic activity of microglia post-ICH [15], we hypothesized that microglia may be significantly phagocytosing and degrading Oe-EXOs within the brain. To investigate this, we conducted an *in vitro* uptake assay by incubating RM with 20 μg of DiI-labeled Oe-EXOs for 24 h. As shown in Supplementary File 1: Fig. S10, abundant red fluorescence was observed in the cytoplasm of RM, indicating that Oe-EXOs were readily phagocytosed by microglia. To address this issue, we utilized CD47 and RBC membrane engineering as a potential solution. Previous studies have shown that RBC membranes, rich in CD47, serve as a biomimetic coating to protect nanoparticles from phagocytosis by the MPS [45]. Therefore, we attempted to engineer Oe-EXOs with anti-phagocytic properties using RBC membranes. After isolating RMDVs, we co-extruded Oe-EXOs with RMDVs at protein weight ratios (Oe-EXOs:RMDVs) of 1:0, 1:1, 1:2, and 1:3. DiI-labeled co-extrusion products were then analyzed via flow cytometry to assess their phagocytosis by RMs. As shown in Fig. 5C–E, in the absence of RMDVs, approximately 77.9 % of RM took up the co-extrusion product. With increasing proportions of RMDVs, RM uptake of the co-extrusion product progressively decreased to 21.2 %, 17.9 %, and 13.5 %, respectively, indicating that RBC membranes effectively prevented Oe-EXOs from being phagocytosed by microglia. Consequently, we adopted a 1:3 protein weight ratio (Oe-EXOs: RMDVs) for anti-phagocytic engineering of Oe-EXOs in subsequent experiments. To further validate the anti-phagocytic modification, we employed confocal microscopy. As illustrated in Fig. 5G, the co-extrusion product at a 1:3 ratio exhibited significantly reduced presence in the cytoplasm of RM compared to the 1:0 control, consistent with flow cytometry results. Following initial confirmation of anti-phagocytic functionality, we verified the fusion of Oe-EXOs with RMDVs. As shown in Fig. 5I, DiI-labeled Oe-EXOs and DiO-labeled RMDVs were co-extruded at a 1:3 protein weight ratio, with the resulting product (m-Oe-EXOs) in the third tube suggesting effective fusion of Oe-EXOs and RMDVs. WB was subsequently performed to analyze specific protein markers (Fig. 5J). Three typical exosomal markers—CD9, CD63, and TSG101—and CD47, expressed on the RBC membrane, were all detected on m-Oe-EXOs. Further, we characterized the morphology, diameter, and zeta potential of m-Oe-EXOs. As shown in Fig. 5K–O, Oe-EXOs maintained their exosomal structure post-co-extrusion with RMDVs, with a diameter and zeta potential of approximately 80.27 nm and −24.29 mV, slightly reduced compared to EPC-EXOs but still within the acceptable range for exosomes. Finally, we assessed the uptake of m-Oe-EXOs by RBMECs, HT22 neurons, and CTX-TNA2 astrocytes. Both Oe-EXOs and m-Oe-EXOs were labeled with DiI. As shown in Supplementary File 1: Fig. S11, flow cytometry analysis revealed that only approximately 11.6 % of HT22 cells and 14.8 % of CTX-TNA2 cells internalized Oe-EXOs, indicating a relatively low uptake rate for these two cell types. Notably, the uptake rates of m-Oe-EXOs by these cells were not significantly different from those of Oe-EXOs. Moreover, as shown in Fig. 5D–F, the uptake rates of RBMECs for m-Oe-EXOs and Oe-EXOs were nearly identical, around 90 %. This observation was further corroborated by confocal imaging (Fig. 5H). Collectively, these data indicate that EPC-EXOs are susceptible to phagocytosis by microglia, and engineering EPC-EXOs with RBC membranes effectively prevents this.

### 3.6 m-Oe-EXOs exhibit enhanced anti-phagocytic properties *in vivo* and provide therapeutic benefits in ICH rats

We next evaluated the therapeutic potential of m-Oe-EXOs in rats with ICH, focusing initially on their improved resistance to phagocytosis. Following the protocol described above, we administered 100 μL of DiI-labeled m-Oe-EXOs into the brains of ICH rats via SI 1 h post-collagenase injection. After injection, rats were sacrificed at 3, 6, 9, 12, and 24 h, and whole brain tissues were harvested. The small animal live imaging demonstrated a notably slower degradation of m-Oe-EXOs in the brain tissue compared to non-engineered exosomes, suggesting an enhanced anti-phagocytic capability post-modification (Fig. 5A and B).

**Fig. 6:**
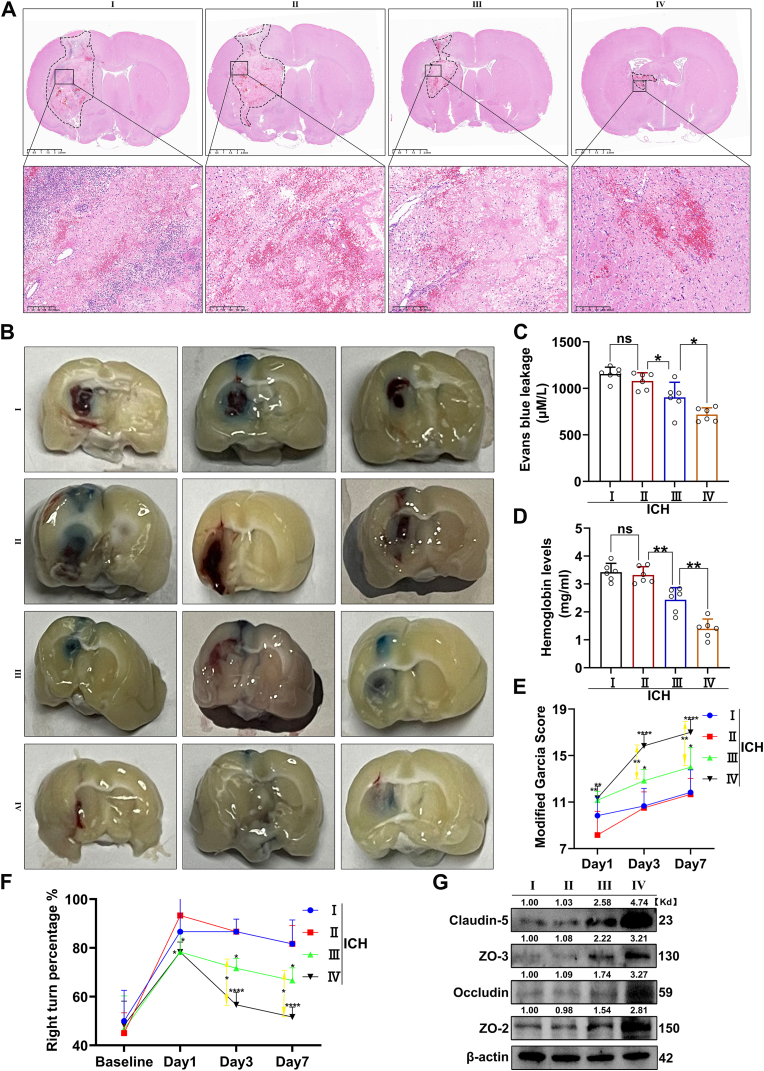
Therapeutic efficacy of m-Oe-EXOs delivered by SI and endoscope in a rat model of collagenase-induced ICH. (A) HE staining of brain tissue on day 3 post-ICH, with the dotted line area indicating the hemorrhagic lesion. (B) Stereogram of brain tissue on day 3 post-ICH, with the blue substance representing Evans Blue dye leaked into the brain. (C) Evans Blue leakage assay measuring the content of Evans Blue dye in the ipsilateral brain tissue of ICH rat. N = 6. (D) Hemoglobin content in the ipsilateral brain tissue of ICH rat. N = 6. (E) Modified Garcia Scores of rats on days 1, 3, and 7 post-ICH. N = 6. (F) Corner turn test results of rat on days 1, 3, and 7 post-ICH. N = 6. (G) Western blotting analysis of four major tight junction proteins: Claudin-5, ZO-3, Occludin and ZO-2 (ns indicates no significant, ∗p < 0.05, ∗∗p < 0.01, ∗∗∗p < 0.001, ∗∗∗∗p < 0.0001). Ⅰ: PBS delivered by SI; Ⅱ: PBS delivered by nasal endoscope; Ⅲ: m-Oe-EXOs delivered by SI; Ⅳ: m-Oe-EXOs delivered by nasal endoscope. (For interpretation of the references to color in this figure legend, the reader is referred to the Web version of this article.)

Subsequently, we conducted SI of either 100 μL m-Oe-EXOs or an equivalent volume of PBS in ICH rats. On day three post-injection, brain tissues were collected and subjected to HE staining. As shown in Fig. 6A, m-Oe-EXO treatment resulted in a smaller brain injury area than PBS and reduced pathological damage surrounding the hematoma. Additionally, the Evans Blue Leakage Assay indicated lower dye levels in the hemorrhagic brain tissue following m-Oe-EXO injection, demonstrating reduced BBB disruption in m-Oe-EXO-treated rats (Fig. 6B and C). Furthermore, BBB integrity was evaluated by analyzing the expression of key tight junction proteins in the cerebral hemispheres on the hemorrhagic side (excluding the olfactory bulb, cerebellum, and brainstem) on the third day post-ICH induction. Total protein was extracted from the brain tissue, and WB analysis was performed to assess changes in four major tight junction proteins: Claudin-5, ZO-3, Occludin and ZO-2. The results demonstrated that m-Oe-EXO treatment upregulated the expression levels of Claudin-5, ZO-3, Occludin and ZO-2 compared to the PBS-treated group (Fig. 6G). Together with the findings from the Evans Blue Leakage Assay, these results provide robust evidence supporting the reduced BBB disruption in m-Oe-EXO-treated rats. Hemoglobin quantification further revealed a smaller hematoma size in the m-Oe-EXO group (Fig. 6D). We then assessed NLF recovery using the Modified Garcia Test and the Corner Test. As illustrated in Fig. 6E and F, both groups exhibited progressive recovery over time, with Modified Garcia Scores increasing and right-turn ratios decreasing. Specifically, rats treated with m-Oe-EXOs showed a significantly enhanced recovery of NLF compared to the PBS group. Finally, to assess angiogenesis within the brain injury region, immunofluorescent staining for CD31 and CD34 was performed on brain tissues collected seven days post-injection. As depicted in Supplementary File 1: Fig. S12, m-Oe-EXO-treated rats demonstrated increased angiogenesis in the injured area. In summary, these findings indicate that m-Oe-EXOs display robust anti-phagocytic capabilities *in vivo* and provide therapeutic benefits in ICH rats by reducing BBB damage, minimizing hematoma size, promoting angiogenesis, and facilitating NLF recovery.

### 3.7 Minimally invasive endoscopic-guided targeted delivery of m-oe-EXOs offers enhanced therapeutic efficacy compared to SI in ICH treatment

Given the high invasiveness of SI, which limits clinical translation, we developed a minimally invasive, endoscope-assisted intranasal delivery method for m-Oe-EXOs. This approach aims to provide therapeutic benefits while minimizing invasive damage. As demonstrated in the Supplementary File 2, under endoscopic guidance, m-Oe-EXOs were accurately delivered to the olfactory mucosa in the deep nasal cavity, an optimal region for nasal-to-brain transport.

To evaluate brain distribution, we administered 100 μL of DiI-labeled m-Oe-EXOs into the bilateral nasal cavities (50 μL per side) of ICH rats 1 h post-collagenase injection, targeting the olfactory mucosa under endoscopic visualization. Fig. 5A, B shows that, compared to SI, m-Oe-EXO levels in brain tissue were lower at 3 h post-delivery but gradually increased from 3 to 9 h, surpassing levels achieved by SI at a certain point within this period. From 9 to 24 h post-delivery, m-Oe-EXO levels decreased but remained higher than those observed with SI.

Next, we compared the therapeutic effects of endoscope-guided intranasal delivery of m-Oe-EXOs versus SI in ICH rats. As shown in Fig. 6A, endoscopic delivery resulted in a significantly reduced brain injury area and diminished pathological damage surrounding the hematoma. Additionally, the Evans Blue Leakage Assay indicated that endoscopic delivery of m-Oe-EXOs led to a notably lower Evans Blue dye concentration in hemorrhagic brain tissue, signifying reduced BBB disruption in ICH rats (Fig. 6B and C). Moreover, the WB analysis of major tight junction proteins corroborated that endoscopic delivery of m-Oe-EXOs reduced BBB disruption in ICH rats compared to the SI administration (Fig. 6G). Hemoglobin measurement further confirmed that endoscopic delivery of m-Oe-EXOs significantly reduced hematoma size compared to SI (Fig. 6D). Moreover, NLF tests revealed that endoscopic delivery of m-Oe-EXOs led to a notably better recovery of NLF (Fig. 6E and F). Immunofluorescent staining further demonstrated increased angiogenesis in the brain injury area following endoscopic delivery of m-Oe-EXOs (Supplementary File 1: Fig. S12). Finally, we assessed the biosafety of endoscopic delivery of m-Oe-EXOs by analyzing peripheral blood and major organs (heart, liver, spleen, lungs, and kidneys) on day 7 post-ICH. Supplementary File 1: Fig. S13 showed no abnormal pathological findings in these organs, and Supplementary File 1: Fig. S14 confirmed no significant deviations in main serum biochemical indices from normal thresholds. Collectively, these findings demonstrate that endoscope-guided intranasal delivery enables effective m-Oe-EXO entry into brain tissue, maintaining high tissue concentrations. Compared to SI, this method provides a less invasive and more effective therapeutic approach for ICH treatment.

## 4 Discussion

To date, ICH has remained a severe type of stroke caused by the rupture of blood vessels within the brain parenchyma, resulting in primary and secondary brain injuries [46]. BMECs, a critical component of the BBB, suffer dysfunction due to the substantial release of heme following ICH, which further impedes the repair of damaged blood vessels and the BBB [5,47]. Although previous studies have indicated that extracellular vesicles derived from EPCs can activate angiogenic programs in ECs via horizontal mRNA transfer and promote angiogenesis through Erk1/2 signaling accelerating cutaneous wound healing [12,48], the effects of EPC-EXOs on BMEC dysfunction post-ICH remain unexplored. In this study, EPCs were successfully isolated and cultured from rat BM cells, and EPC-EXOs were obtained from their supernatant. We developed an *in vitro* model of ICH using RBMECs and investigated the effects of EPC-EXOs on RBMECs proliferation, migration, and angiogenesis. Through proteomic analysis, we identified HSP90 as a key protein within EPC-EXOs that facilitates RBMEC function, exerting its effects via the Akt signaling pathway. To enhance therapeutic efficacy and reduce phagocytic clearance, we engineered EPC-EXOs with anti-phagocytic properties and delivered them using a minimally invasive, endoscopic-guided targeted delivery system in an ICH rat model. In summary, our study presents three key innovations: 1. EPC-EXOs as a novel therapeutic strategy for ICH: This is the first study to utilize EPC-EXOs—a highly promising tool in regenerative medicine—for the treatment of ICH. Both *in vitro* and *in vivo* experiments demonstrated that EPC-EXOs promote RBMEC functional recovery, reduce hematoma size, enhance angiogenesis, and facilitate NLF recovery following ICH. Mechanistically, we provided evidence for the role of the Akt signaling pathway mediated by exosomal HSP90. 2. Enhanced anti-phagocytic capability of EPC-EXOs: We creatively adapted a well-established technique by fusing RBC membranes with EPC-EXOs. This approach effectively mitigated the phagocytosis of EPC-EXOs by microglia. The anti-phagocytic properties of these engineered EPC-EXOs were rigorously validated through flow cytometry and other assays. 3. Development of a novel delivery system: Recognizing the high invasiveness of conventional SI which limits clinical translation, we designed and developed a proprietary minimally invasive, endoscopic-guided targeted delivery system. This system enables the precise delivery of m-Oe-EXOs to the olfactory mucosa in the deep nasal cavity (Supplementary File 2), facilitating brain entry while significantly reducing invasiveness and enhancing the feasibility of clinical translation.

The characterization of EPCs showed high expression of CD133 (a stemness marker) and endothelial surface antigens, such as CD31, CD34 and vWF [49,50], with immunocytochemistry and flow cytometry confirming successful EPC isolation. The uptake of Ac-LDL and UEA-1 staining further validated the identity of the isolated cells [51,52]. Additionally, EPC-EXOs were purified from EPC supernatant and characterized using WB and other established methods.

Next, we established an *in vitro* ICH model using RBMECs to simulate their conditions within the brain. After confirming that EPC-EXOs could be internalized and utilized by RBMECs, we treated the RBMECs with varying concentrations of EPC-EXOs. Through assessments of cell proliferation, migration, and angiogenesis, we demonstrated that EPC-EXOs significantly enhance the functional capabilities of RBMECs. Given the diverse functional components in exosomes, including RNA, DNA, proteins, glycans, lipids, and metabolites [53], we focused on EPC-EXOs proteins to elucidate their mechanism of action on RBMEC function. Employing proteomic analysis—a novel approach in exosome research—we identified 3972 proteins from five EPC-EXO samples. KEGG pathway enrichment indicated significant involvement of the PI3K-Akt signaling pathway, narrowing our focus to HSP90 as a highly abundant EPC-EXOs protein within this pathway. WB validation confirmed HSP90's elevated expression in EPC-EXOs.

To verify HSP90's role in RBMEC function, we employed lentiviral transduction to generate Oe-EXOs or Sh-EXOs, confirming that exosomal HSP90 is pivotal in enhancing RBMEC functionality. Next, we further investigated the downstream pathways through which HSP90 regulates RBMEC function. Using Akt agonists and inhibitors, we demonstrated that HSP90 activates the Akt pathway in RBMECs, thereby enhancing their functional capabilities.

Engineering exosomes is an effective strategy for endowing them with new functional properties [54,55]. In this study, we pioneered an anti-phagocytic engineering approach by coating EPC-EXOs with RBC membranes enriched in CD47 using a co-extrusion technique. This modification aims to reduce phagocytic degradation by microglia with enhanced phagocytic activity post-ICH [15]. We assessed these engineered EPC-EXOs across multiple dimensions, including brain distribution, morphology, particle size, and zeta potential. Finally, we utilized a proprietary minimally invasive endoscopic-guided targeted delivery system for intranasal delivery of anti-phagocytic EPC-EXOs to treat rats with ICH (Supplementary File 2). We demonstrated that this approach enables efficient entry of m-Oe-EXOs into brain tissue, maintaining high tissue concentrations. Additionally, we evaluated therapeutic outcomes through HE staining, Evans Blue leakage assay, hemoglobin detection, neurological function assessments, and immunofluorescence to assess BBB integrity, hematoma size, angiogenesis, and neurological recovery post-ICH. Our findings confirm that endoscopic-guided delivery of m-Oe-EXOs provides substantial therapeutic benefits for ICH.

Taken together, our study is the first to utilize a proprietary minimally invasive endoscopic-guided targeted delivery system for delivering anti-phagocytic EPC-EXOs to treat ICH. However, there are limitations to consider. First, our study focuses only on RBMECs post-ICH, evaluating the therapeutic effect of EPC-EXOs only from the perspective of RBMECs, while mechanisms involving other cell types remain unexplored. Second, due to time and resource constraints, our research mainly delves into the PI3K-Akt signaling pathway and the role of exosomal HSP90. The broader implications of other proteins identified through exosomal proteomics analysis (Supplementary File 4), particularly their effects on different cell types and diseases, should be addressed in future studies. Third, for enhanced clinical translation, it is crucial to elucidate the mechanisms through which intranasally delivered exosomes enter the brain from the olfactory mucosa. Lastly, we have demonstrated therapeutic effects only in ICH rats, so further translational research in humans is essential.

## 5 Conclusion

Our study presents a novel, minimally invasive, and highly effective therapeutic strategy for ICH using endoscopic-guided intranasal delivery of engineered EPC-EXOs. By focusing on the role of RBMECs, we demonstrated that EPC-EXOs can significantly promote RBMEC functions, including proliferation, migration, and angiogenesis, crucial for the repair of BBB integrity post-ICH. Furthermore, our proteomic analysis identified HSP90 as a key molecular mediator in EPC-EXOs, activating the Akt signaling pathway to enhance the functional recovery of RBMECs. The modification of EPC-EXOs with anti-phagocytic properties further improved their therapeutic potential, enabling sustained brain distribution and reduced degradation by microglia. Through this innovative delivery system, we showed that EPC-EXOs effectively ameliorate brain injury, reduce hemorrhagic damage, and promote angiogenesis in ICH rats, with no significant adverse effects observed. In summary, our findings offer new insights into exosome-based therapy for ICH, emphasizing the potential of minimally invasive delivery systems for clinical translation and holding promise for improved patient outcomes in the future.

## CRediT authorship contribution statement

**Gui Wan:** Writing – original draft, Conceptualization. **Zhenwei Li:** Writing – original draft, Conceptualization. **Lingui Gu:** Methodology. **Ye Sun:** Methodology. **Yuhe Wang:** Investigation. **Yiqing Wang:** Writing – review & editing. **Ruxu Geng:** Investigation. **Yangyang Chen:** Writing – review & editing. **Wenbin Ma:** Supervision, Resources. **Xinjie Bao:** Supervision, Resources, Funding acquisition. **Renzhi Wang:** Supervision, Resources, Funding acquisition.

## Funding

This work was supported by the 10.13039/501100008235Peking Union Medical College Hospital Outstanding Young Talent Development Program (UBJ11023), the 10.13039/501100012166National Key Research and Development Program of China (2018YFA0108600), the National High Level Hospital Clinical Research Funding (2022-PUMCH-C-042), the 10.13039/501100001809National Natural Science Foundation of China (82170799, 82171475), the Special Research Fund for Central Universities, 10.13039/501100011176Peking Union Medical College (3332024110), the CAMS Initiative for 56 lnnovative Medicine (2021-1-12M−019), Science Technology Innovation Commission of Shenzhen Municipality, 10.13039/501100017609Shenzhen Knowledge Innovation Program - Shenzhen Key Project in Basic Research (JCYJ20220818103007014) and 2022 Shenzhen Higher Education Institutions Stability Support Project.

## Declaration of competing interest

The authors declare no competing interests.

## Data Availability

Data will be made available on request.

## Abbreviations

ICH: intracerebral hemorrhage
BBB: blood-brain barrier
EPCs: endothelial progenitor cells
ECs: endothelial cells
NLFs: neurological functions
EPC-EXOs: exosomes derived from endothelial progenitor cells
MPS: mononuclear phagocyte system
SIRPα: signal regulatory protein alpha
SI: stereotaxic injection
BM: bone marrow
DiI-Ac-LDL: DiI-acetylated low-density lipoprotein
FITC-UEA-1: FITC labeled Ulex europaeus agglutinin 1
TEM: transmission electron microscope
WB: Western Blot
RBMECs: Rat brain microvascular endothelial cells
PBS: phosphate-buffered saline
Ev: empty vector
NC: negative control
RBCs: red blood cells
RMDVs: RBC membrane-derived vesicles
RM: rat microglia
