# Supplementary material for: Endoscopic nasal delivery of engineered endothelial progenitor cell-derived exosomes improves angiogenesis and neurological deficits in rats with intracerebral hemorrhage
Source: Mater Today Bio. 2025 Mar 11;32:101652. doi: 10.1016/j.mtbio.2025.101652 (PMC11953990; doi:10.1016/j.mtbio.2025.101652)
Supplement: Multimedia component 1 [file mmc1.docx]

**Supplementary Material**

**Endoscopic Nasal Delivery of Engineered Endothelial Progenitor Cell-Derived Exosomes Improves Angiogenesis and Neurological Deficits in Rats with Intracerebral Hemorrhage**

Gui Wan^1,#^ · Zhenwei Li ^1,#^ · Lingui Gu^1,#^ · Ye Sun^1^ · Yuhe Wang^1^ · Yiqing Wang^1^ · Ruxu Geng^1^ · Yangyang Chen^4^ · Wenbin Ma^1*^ · Xinjie Bao^1,3*^ · Renzhi Wang^1,2*^

#These authors contributed equally to this work.

*Corresponding author

Xinjie Bao: baoxinjie1@pumch.cn

Wenbin Ma: mawb2001@hotmail.com

Renzhi Wang: wangrz@126.com

**1. Materials and methods**

**1.1 Animals**

Normal male Sprague-Dawley rats (approximately 250 g) were obtained from the Animal Center of Spiff Biotechnology Co., Ltd. (Beijing). The rats were housed at room temperature (22 ± 1°C) on a 12-hour light/dark cycle (humidity: 60 ± 5%), with ad libitum access to food and water. Our experimental protocols received approval from the Animal Ethics Committee of the Chinese Academy of Medical Sciences and Peking Union Medical College. All animal studies adhered to the National Institutes of Health guidelines for the care and use of laboratory animals and the ARRIVE (Animal Research: Reporting *In Vivo* Experiments) guidelines, with further approval from the Institutional Animal Care and Use Committee of the Chinese Academy of Medical Sciences and Peking Union Medical College.

**1.2 Construction of an *In Vitro* Model of ICH**

*In vitro* models of ICH were generated using Hemin (Solarbio, Beijing, China). RBMECs were seeded and treated with varying concentrations of Hemin for 24 hours. Cell viability was assessed using a CCK-8 kit (Solarbio, Beijing, China), and absorbance values were measured at 450 nm using a microplate reader (Tecan, Männedorf, Switzerland).

**1.3 Total RNA Isolation and qRT‒PCR**

Total RNA was extracted from cells using TRIzol reagent (DP430, TIANGEN) according to the manufacturer's protocol. cDNA synthesis was then performed using HiScript III RT SuperMix for qPCR (+gDNA wiper) (Vazyme, China). qRT-PCR was conducted using AceQ® qPCR SYBR Green Master Mix (Vazyme, China). The relative expression levels of HSP90 were quantified using the 2−ΔΔCt method, with β-actin serving as the internal reference gene for normalization. Moreover, the PCR primers are listed in Table S2 (Supplementary File 1).

**1.4 Haematoxylin‐eosin (HE) Staining**

The paraffin-embedded brain, heart, liver, spleen, lung, and kidney tissues were sectioned into 5-μm thick slices. The slices were then deparaffinized, debenzylated, and stained with HE following standard protocols. After staining, the slides were mounted with neutral balsam and sealed with clean coverslips. Finally, the sections were photographed, and histopathological changes were examined under a microscope.

**1.5 Immunostaining**

The status of brain vasculature in the hemorrhagic lesion area was assessed by incubating sections overnight at 4°C with CD31 and CD34 antibodies. After three PBS washes, the sections were incubated with a secondary antibody (Aspen, China) at room temperature for 1 hour. Images were captured using a microscope.

**1.6 Serum Biochemical Assay**

On the seventh day following ICH, all rats were euthanized after completing the neurobehavioral evaluations. Blood samples were collected via cardiac puncture and stored at 4°C overnight. Serum was separated by centrifugation at 1500 rpm for 15 minutes at 4°C, and the resulting supernatant was used for the analysis of blood biochemical markers.

**2. Supplemental Figures**

**Figure S1**


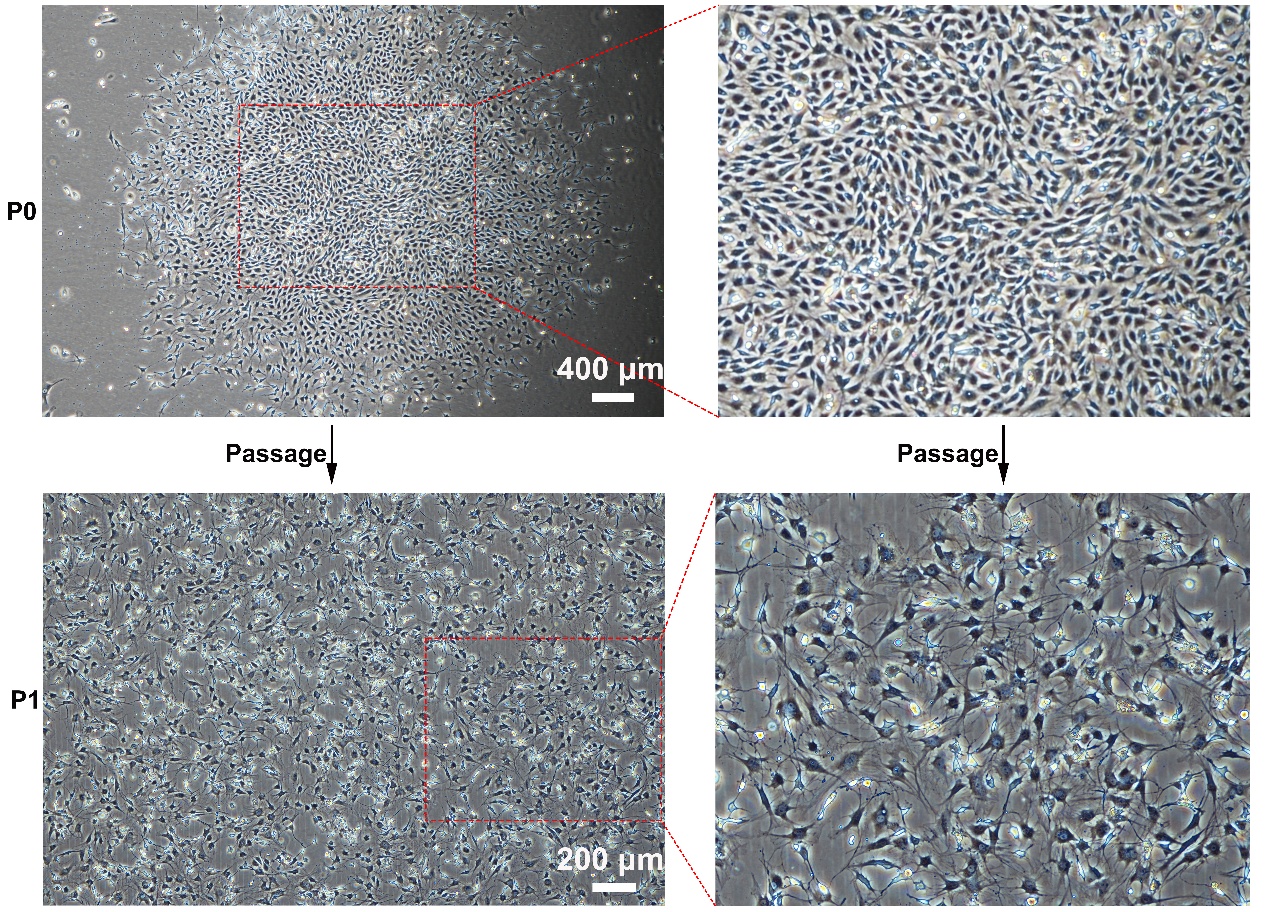


**Fig. S1. Bright-field images of EPCs.**

EPCs exhibited a typical “paving-stone” morphology. P0: Passage 0; P1: Passage 1.

**Figure S2**

**
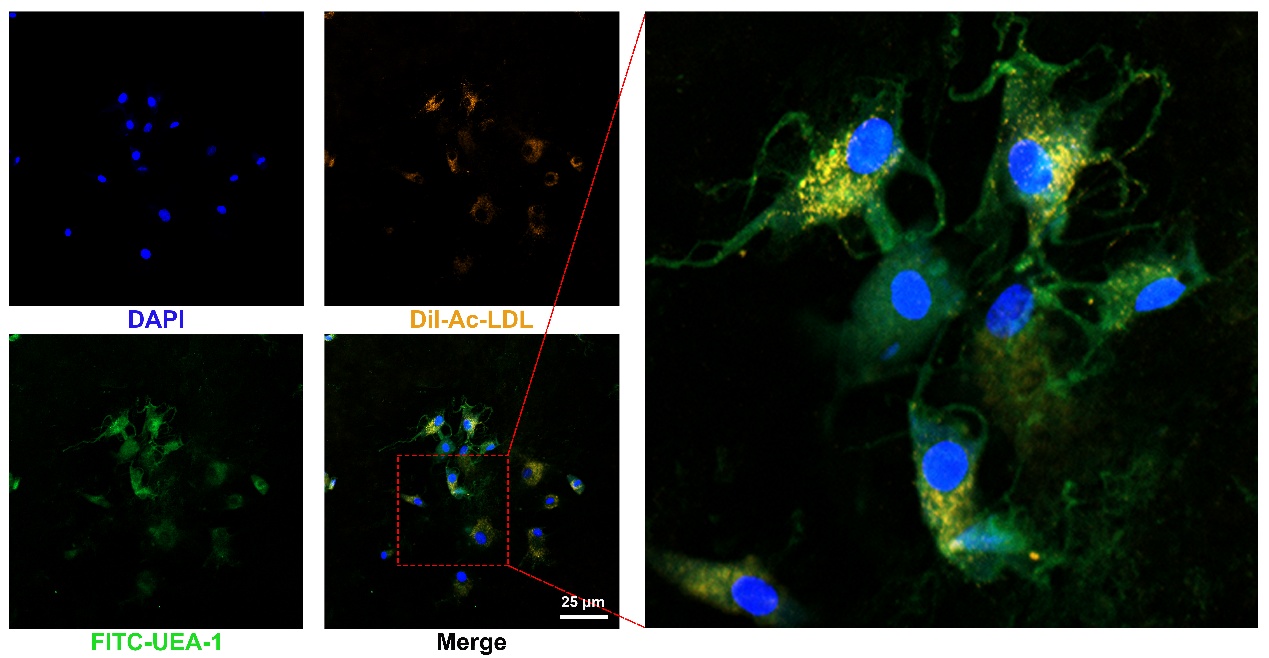
**

**Fig. S2.** EPCs could absorb both DiI-Ac-LDL and FITC-UEA-1. Scale bar: 25 μm.

**Figure S3**

**
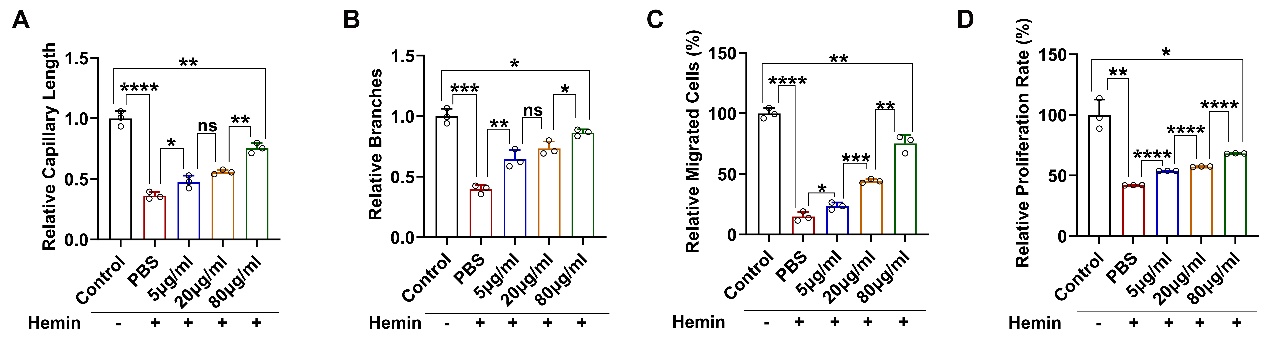
**

**Fig. S3. Statistical analysis of RBMEC functions.**

(A, B) Statistical analysis of Fig. 2C. N = 3. (C) Statistical analysis of Fig. 2D. N = 3. (D) Statistical analysis of Fig. 2E. N = 3. (ns indicates no significant, *p < 0.05, **p < 0.01, ***p < 0.001, ****p < 0.0001).

**Figure S4**

**
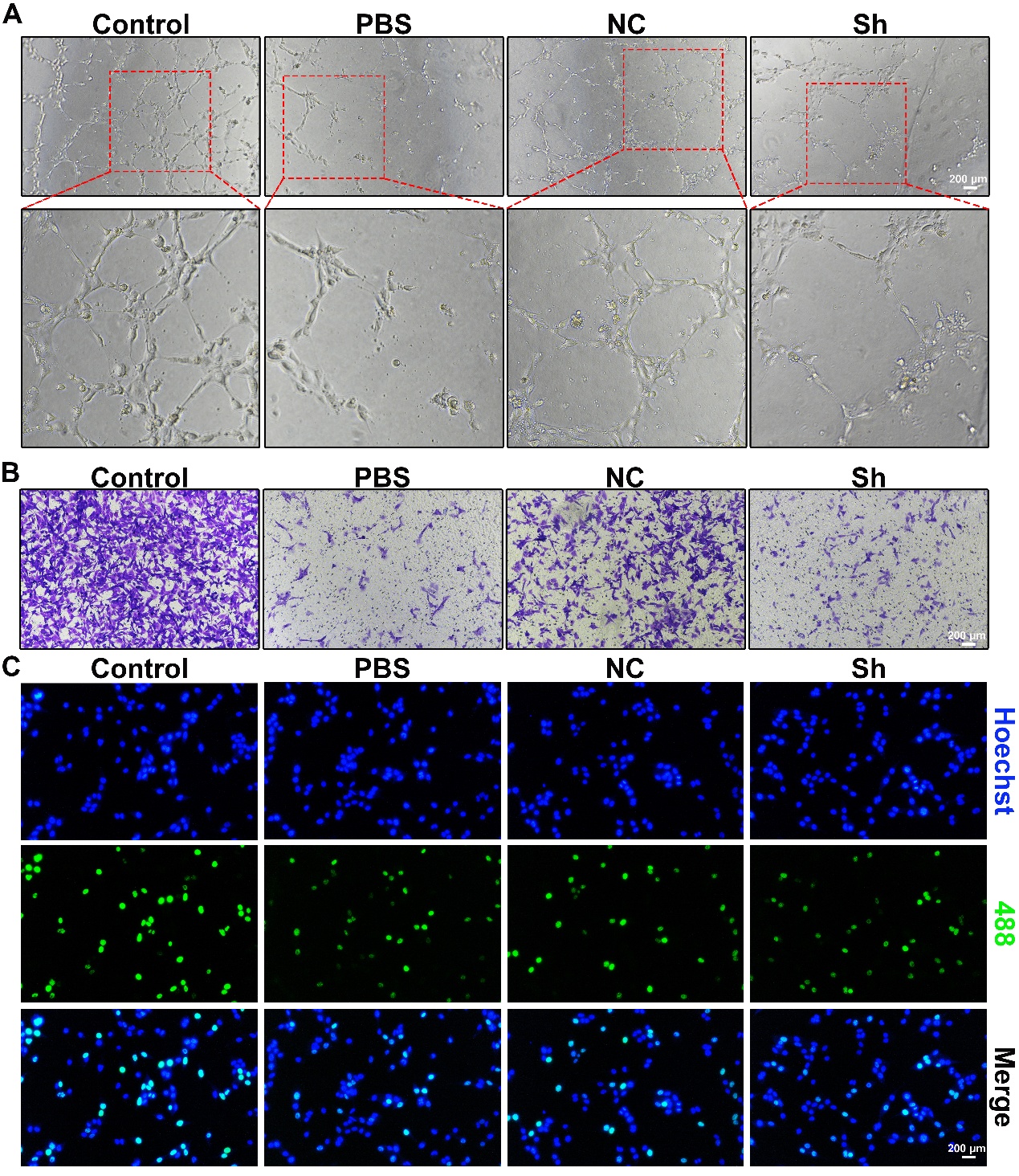
**

**Fig. S4. Exosomal HSP90 enhanced the function of hemin-treated RBMECs.**

(A) Representative images of tube formation of RBMECs. Scale bar: 200 μm. (B) Representative images of migrating RBMECs in transwell assay. The migrating RBMECs stained purple by crystal violet. Scale bar: 200 μm. (C) Representative images of proliferative RBMECs in EdU staining. The proliferative cells were stained with green color, and their cellular nucleis were stained with blue color. Scale bar: 200 μm.

**Figure S5**

**
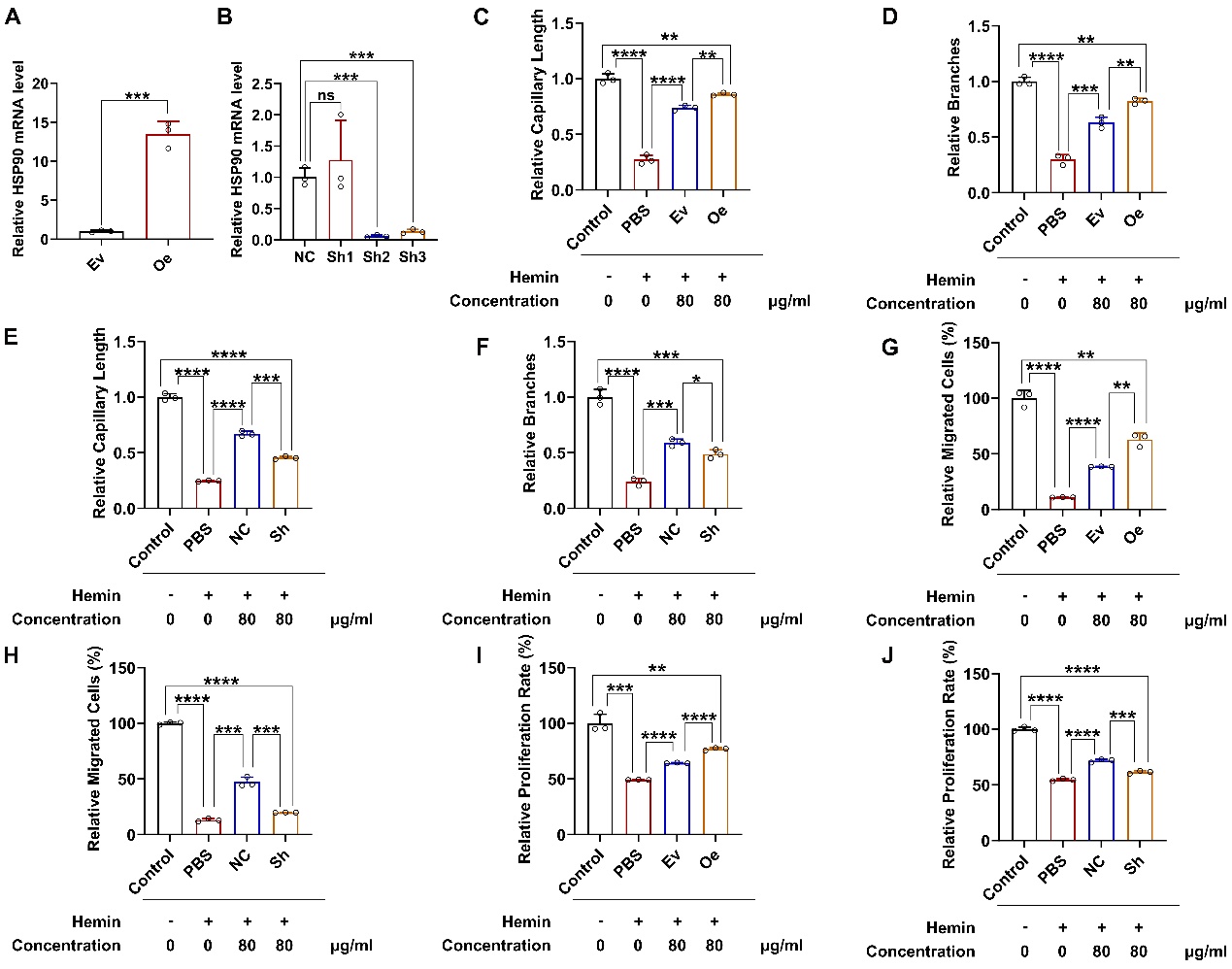
**

**Fig. S5.** (A) The overexpression efficiency determined by qRT-PCR. (B) The knockdown efficiency of different Sh-HSP90 sequence determined by qRT-PCR. (C, D) Statistical analysis of Fig. 3F. N = 3. (E, F) Statistical analysis of Fig. S4A. N = 3. (G) Statistical analysis of Fig. 3G. N = 3. (H) Statistical analysis of Fig. S4B. N = 3. (I) Statistical analysis of Fig. 3H. N = 3. (J) Statistical analysis of Fig. S4C. N = 3. (ns indicates no significant, *p < 0.05, **p < 0.01, ***p < 0.001, ****p < 0.0001).

**Figure S6**

**
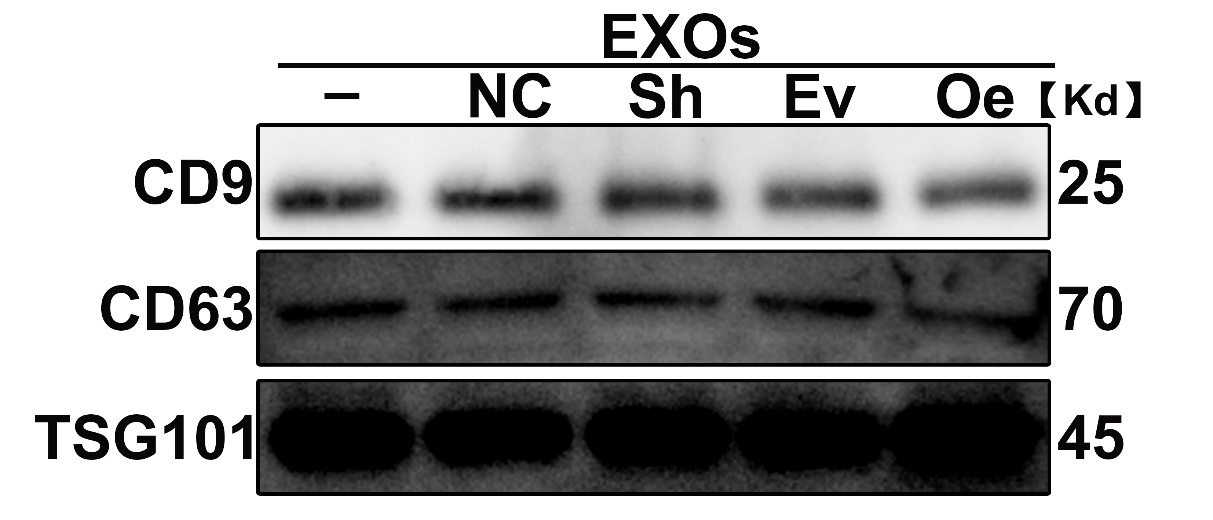
**

**Fig. S6. Identification of exosomal markers in exosomes from cell supernatants.**

The expression of exosomal markers CD9, CD63 and TSG101 in exosomes from EPCs with or without lentiviral infection was analyzed by western blotting assay.

**Figure S7**

**
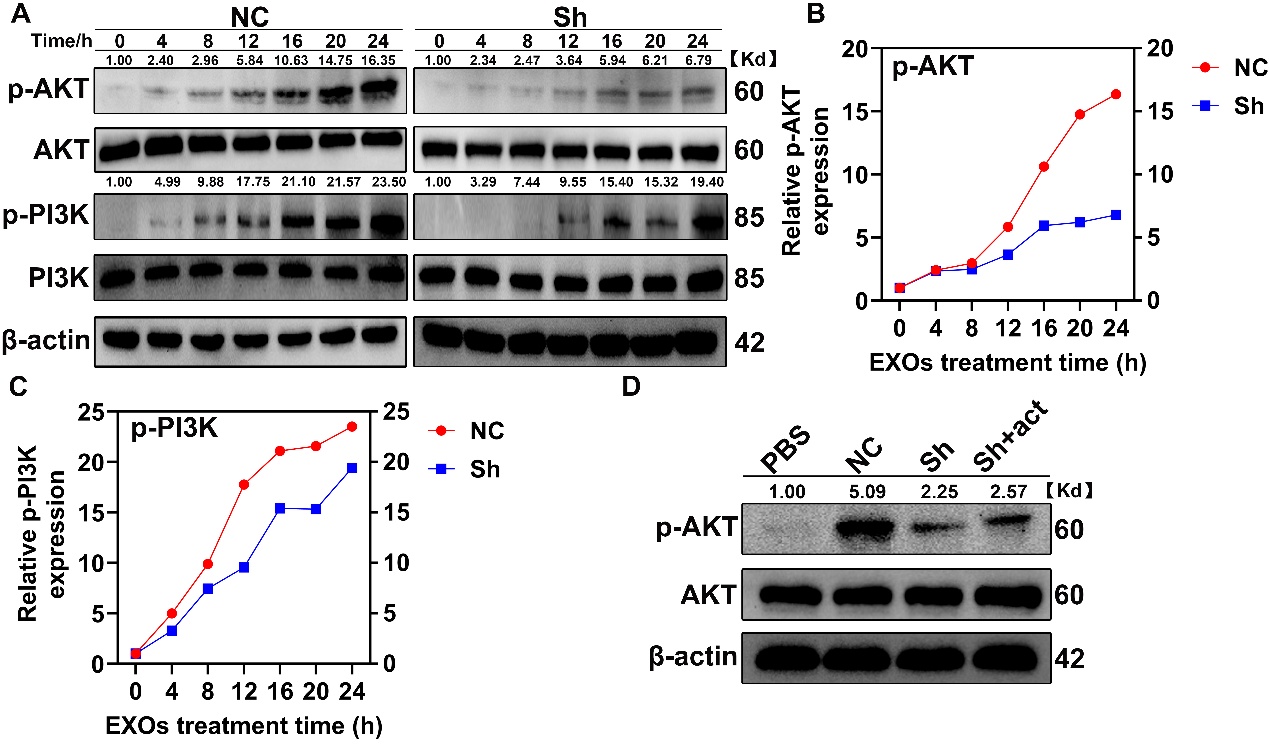
**

**Fig. S7.** (A) Western blot analysis of the activation of Akt pathways in RBMECs after treated with NC-EXOs and Sh-EXOs at different times. (B, C) Data presentation of Fig. S7A. (D) Western blot analysis of the activation of Akt pathways in RBMECs after using SC79.

**Figure S8**

**
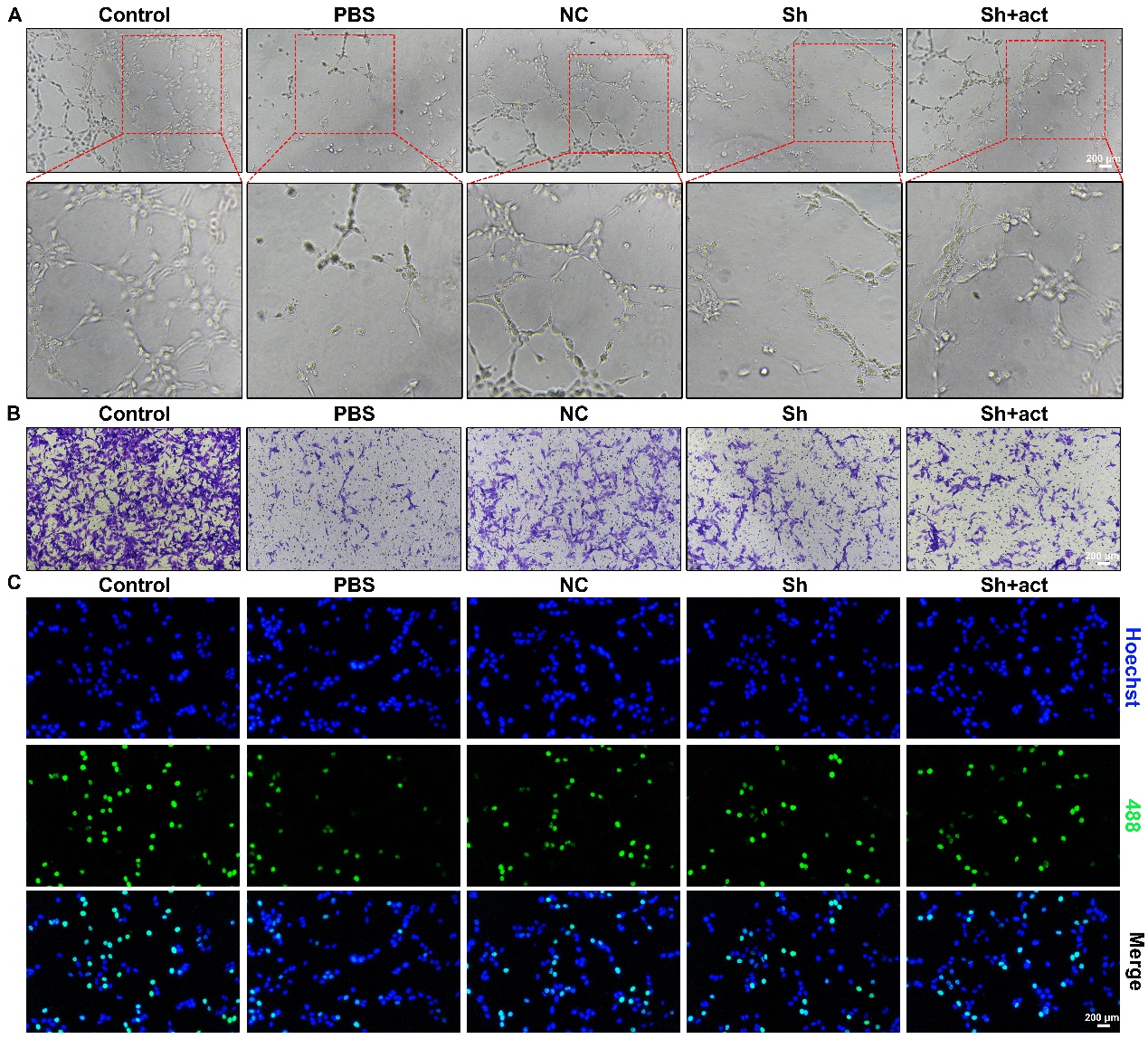
**

**Fig. S8. Exosomal HSP90 enhanced the function of hemin-treated RBMECs by activating the Akt Pathway in RBMECs.**

(A) Representative images of tube formation of RBMECs. Scale bar: 200 μm. (B) Representative images of migrating RBMECs in transwell assay. The migrating RBMECs stained purple by crystal violet. Scale bar: 200 μm. (C) Representative images of proliferative RBMECs in EdU staining. The proliferative cells were stained with green color, and their cellular nucleis were stained with blue color. Scale bar: 200 μm.

**Figure S9**

**
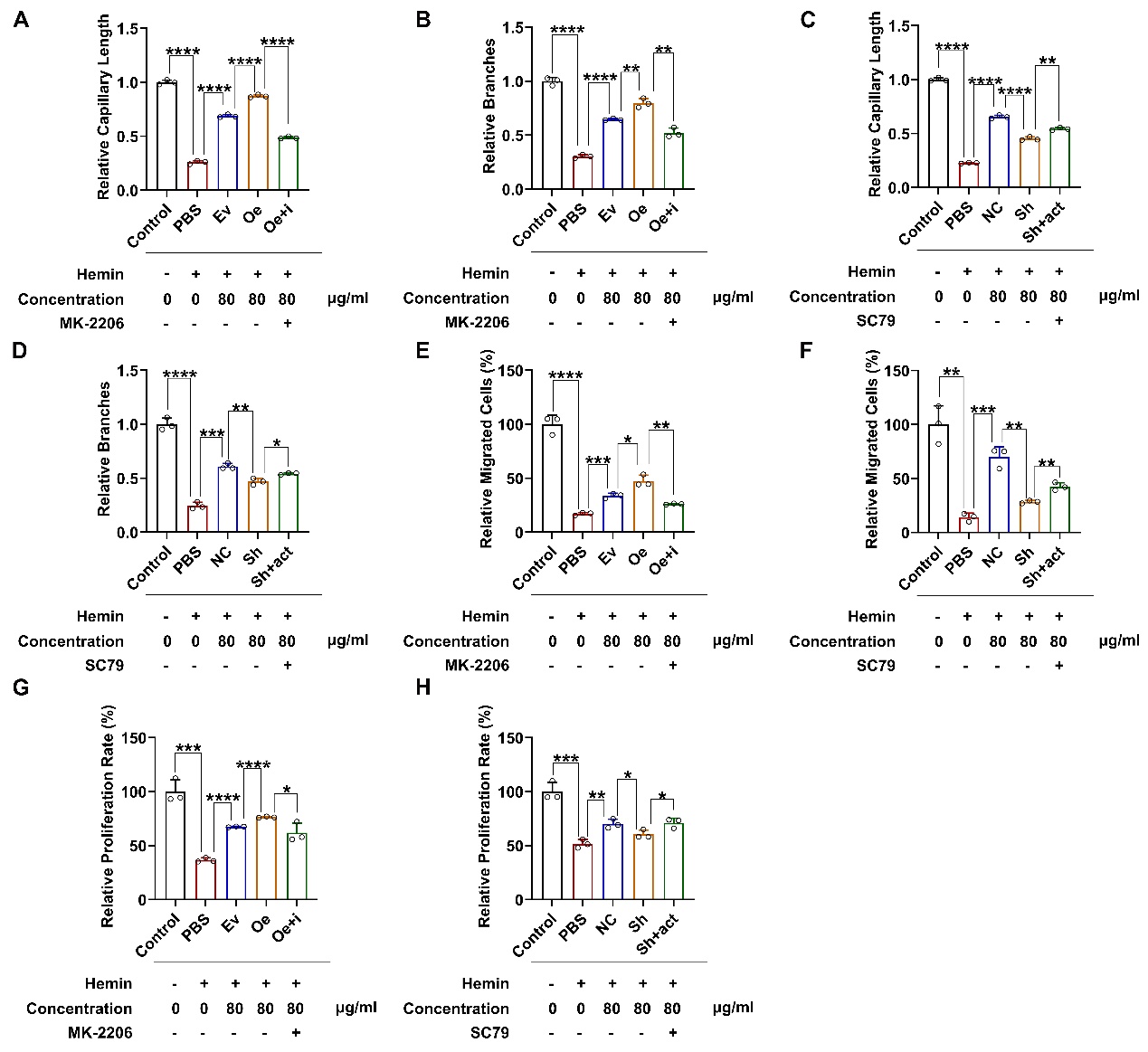
**

**Fig. S9. Statistical analysis of RBMEC functions.**

(A, B) Statistical analysis of Fig. 4D. N = 3. (C, D) Statistical analysis of Fig. S8A. N = 3. (E) Statistical analysis of Fig. 4E. N = 3. (F) Statistical analysis of Fig. S8B. N = 3. (G) Statistical analysis of Fig. 4F. N = 3. (H) Statistical analysis of Fig. S8C. N = 3. (ns indicates no significant, *p < 0.05, **p < 0.01, ***p < 0.001, ****p < 0.0001).

**Figure S10**

**
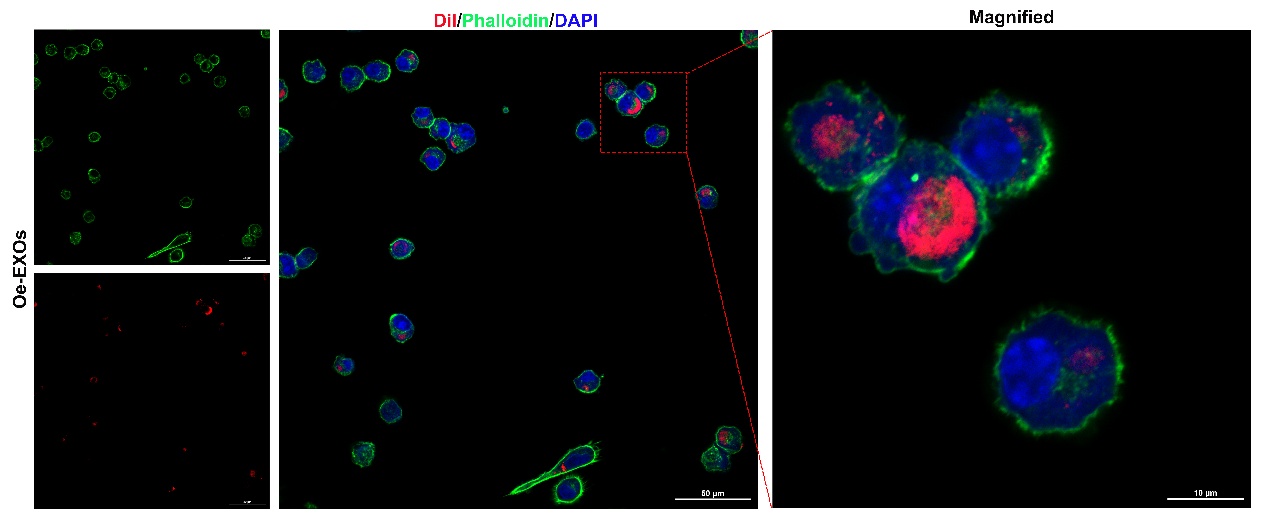
**

**Fig. S10. Confocal images of microglia****.**

Oe-EXOs were readily phagocytosed by microglia. The Oe-EXOs were labeled with red color, the cytoskeleton was stained with green color and their cellular nucleis were stained with blue color. Scale bar: 50 and 10 μm.

**Figure S11**

**
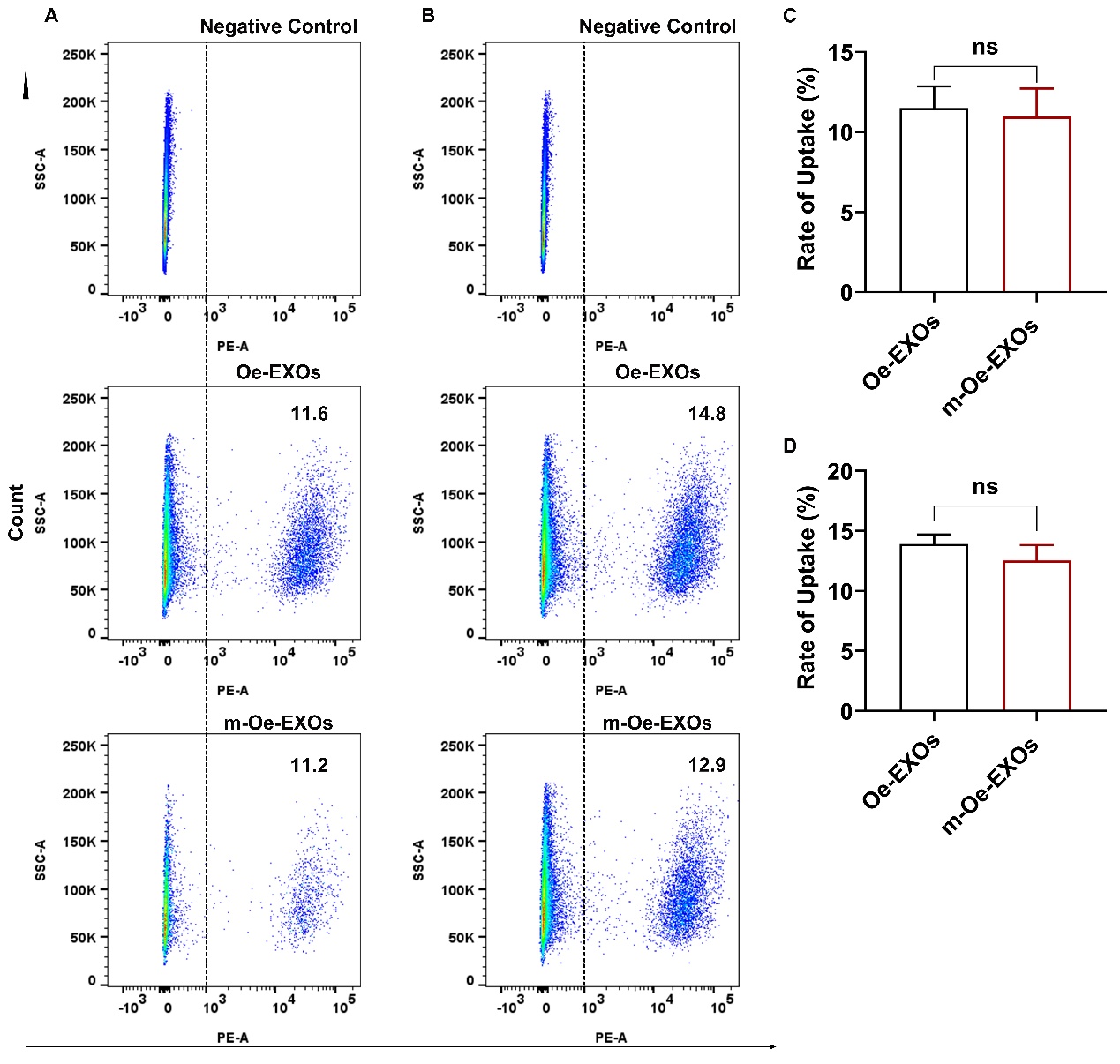
**

**Fig. S11.** (A) Rate of uptake of HT22 detected by flow cytometry analysis. (B) Rate of uptake of CTX-TNA2 detected by flow cytometry analysis. (C) Statistical analysis of Fig. S11A. N = 3. (D) Statistical analysis of Fig. S11B. N = 3. (ns indicates no significant, *p < 0.05, **p < 0.01, ***p < 0.001, ****p < 0.0001).

**Figure S12**

**
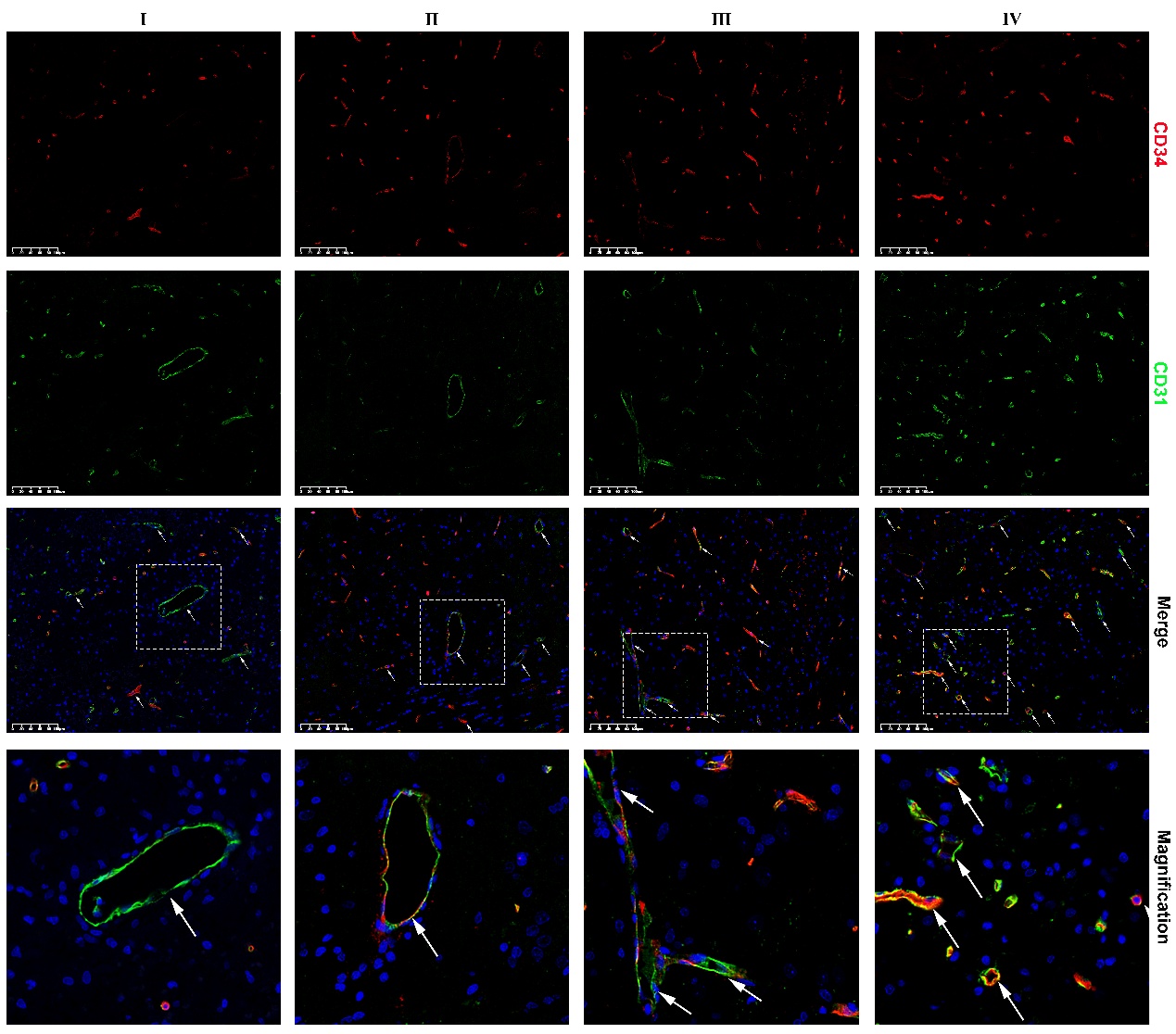
**

**Fig. S12.** Immunofluorescent staining of CD31 and CD34. CD31, CD34 and cell nuclei (DAPI) were stained with green, red and blue, respectively. Scale bar: 100 μm. Ⅰ: PBS delivered by SI; Ⅱ: PBS delivered by nasal endoscope; Ⅲ: m-Oe-EXOs delivered by SI; Ⅳ: m-Oe-EXOs delivered by nasal endoscope.

**Figure S13**

**
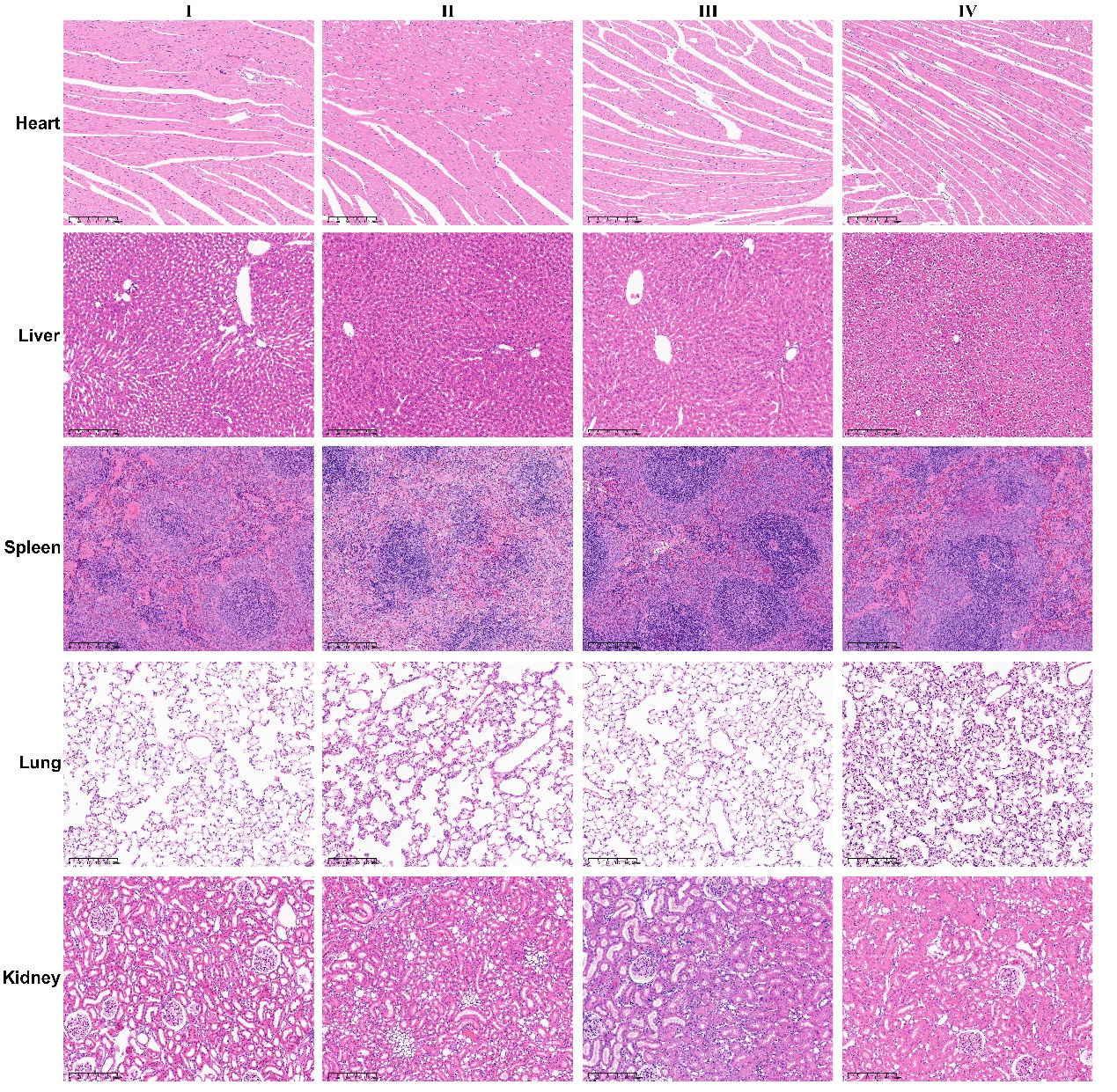
**

**Fig. S13.** Toxicity assessment by HE staining of major organs from ICH rats on day 7 post-ICH. Ⅰ: PBS delivered by SI; Ⅱ: PBS delivered by nasal endoscope; Ⅲ: m-Oe-EXOs delivered by SI; Ⅳ: m-Oe-EXOs delivered by nasal endoscope.

**Figure S14**

**
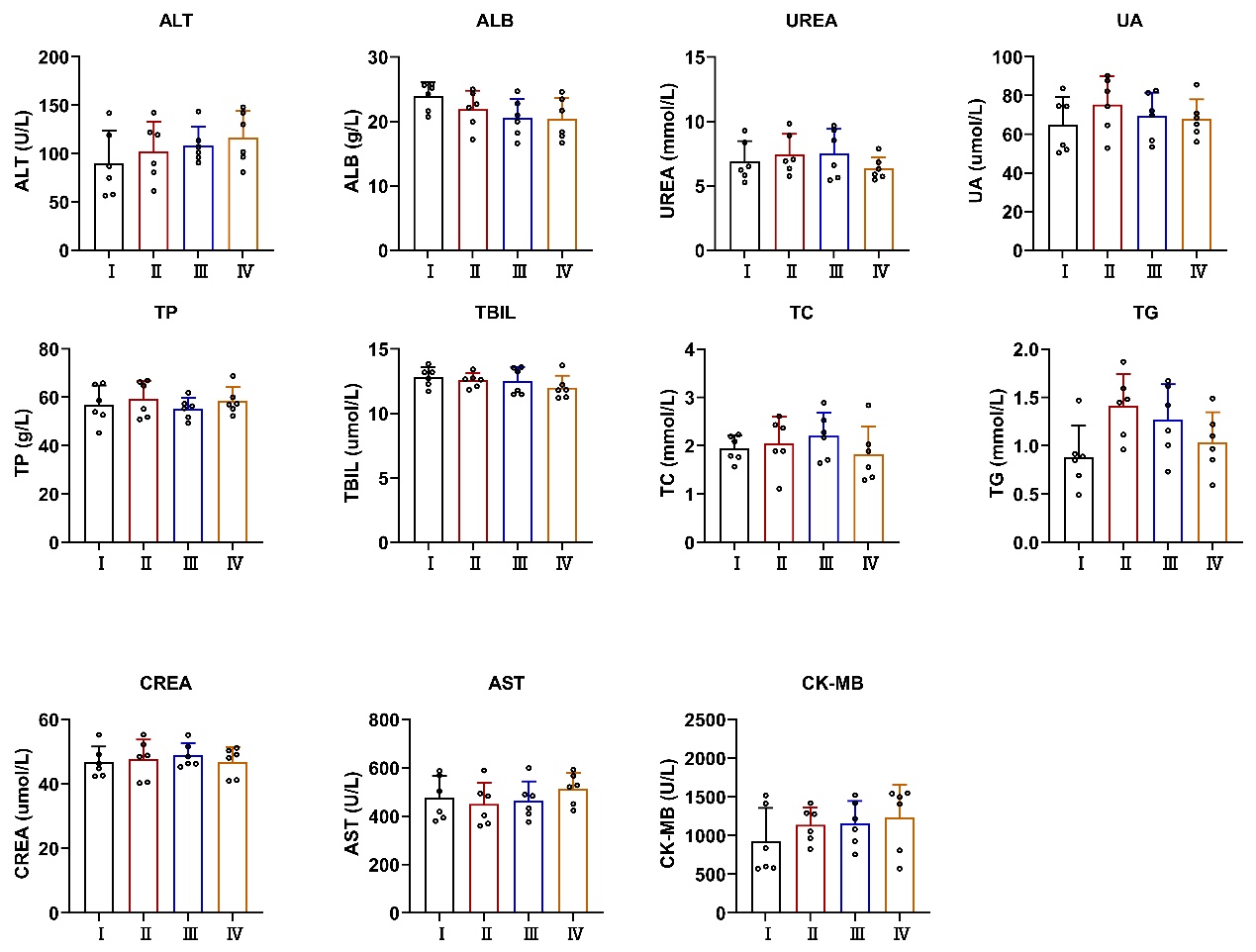
**

**Fig. S14.** Toxicity assessment by serum biochemical analysis of peripheral blood from ICH rats on day 7 post-ICH. N = 6. Ⅰ: PBS delivered by SI; Ⅱ: PBS delivered by nasal endoscope; Ⅲ: m-Oe-EXOs delivered by SI; Ⅳ: m-Oe-EXOs delivered by nasal endoscope.

**3.** **Supplemental Table**

**Table S1: HSP90 ShRNAs sequences**

| Name | Target Sequence (5’ – 3’) |
| --- | --- |
| Sh1 | GCTTGACCGACCCTAGTAAAC |
| Sh2 | GCTGCACATTAATCTCATTCC |
| Sh3 | GCTGGTGCAGATATCTCTATG |

**Table S2: The primer sequences for qRT-PCR analysis**

| HSP90 | Forward | CTTTGAGAACAAGAAGAAGAAGAAC |
| --- | --- | --- |
|  | Reverse | CACACCCTTAATGAAGTTGAGGTAC |
| β-actin | Forward | TCACTGTCCACCTTCCAGCAG |
|  | Reverse | ACGCAGCTCAGTAACAGTCCG |

**Table S3: The antibodies information used in this study**

| Target | Host | Reference | Supplier |
| --- | --- | --- | --- |
| CD34 | Rabbit | ab81289 | Abcam |
| vWF | Rabbit | 27186-1-AP | Proteintech |
| CD31 | Rabbit | 15585 | Cell Signaling Technology |
| CD133 | Rabbit | 18470-1-AP | Proteintech |
| CD9 | Rabbit | ab307085 | Abcam |
| CD63 | Rabbit | 25682-1-AP | Cell Signaling Technology |
| TSG101 | Rabbit | ab125011 | Abcam |
| Calnexin | Rabbit | 10427-2-AP | Proteintech |
| HSP90 | Rabbit | 13171-1-AP | Proteintech |
| p-AKT | Rabbit | 4060 | Cell Signaling Technology |
| AKT | Rabbit | 4691 | Cell Signaling Technology |
| p-PI3K | Rabbit | 310164 | ZEN-BIOSCIENCE |
| PI3K | Rabbit | A11177 | ABclonal |
| β-actin | Rabbit | 20536-1-AP | Proteintech |
| CD47 | Rabbit | 20305-1-AP | Proteintech |
| Claudin-5 | Rabbit | 29767-1-AP | Proteintech |
| ZO-3 | Rabbit | HY-P82711 | MedChemExpress |
| Occludin | Rabbit | 27260-1-AP | Proteintech |
| ZO-2 | Rabbit | 18900-1-AP | Proteintech |
