# Supplementary material for: Endoscopic nasal delivery of engineered endothelial progenitor cell-derived exosomes improves angiogenesis and neurological deficits in rats with intracerebral hemorrhage
Source: Mater Today Bio. 2025 Mar 11;32:101652. doi: 10.1016/j.mtbio.2025.101652 (PMC11953990; doi:10.1016/j.mtbio.2025.101652)

Figure 1D-CD9

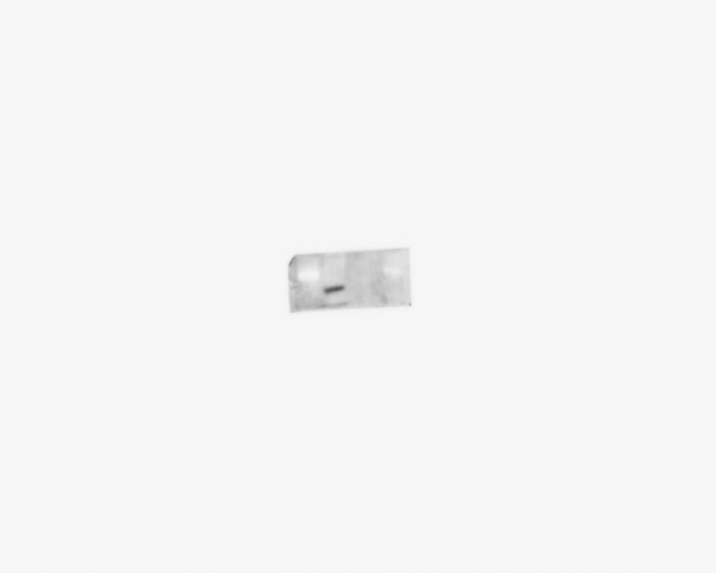

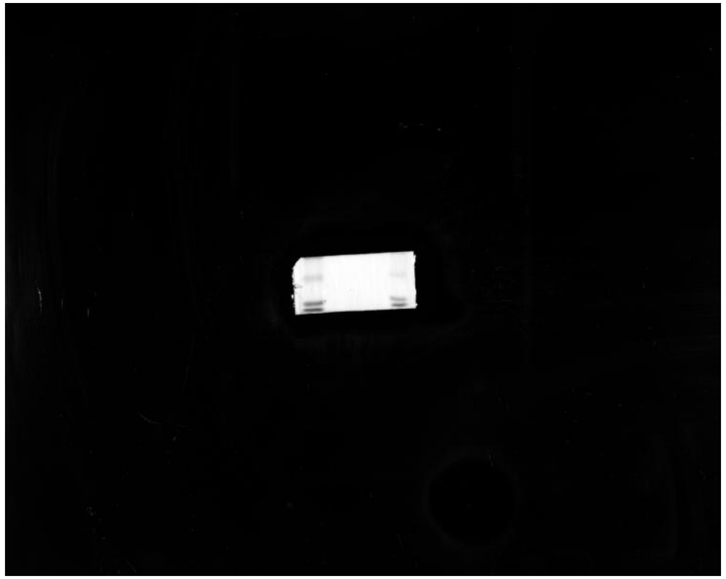

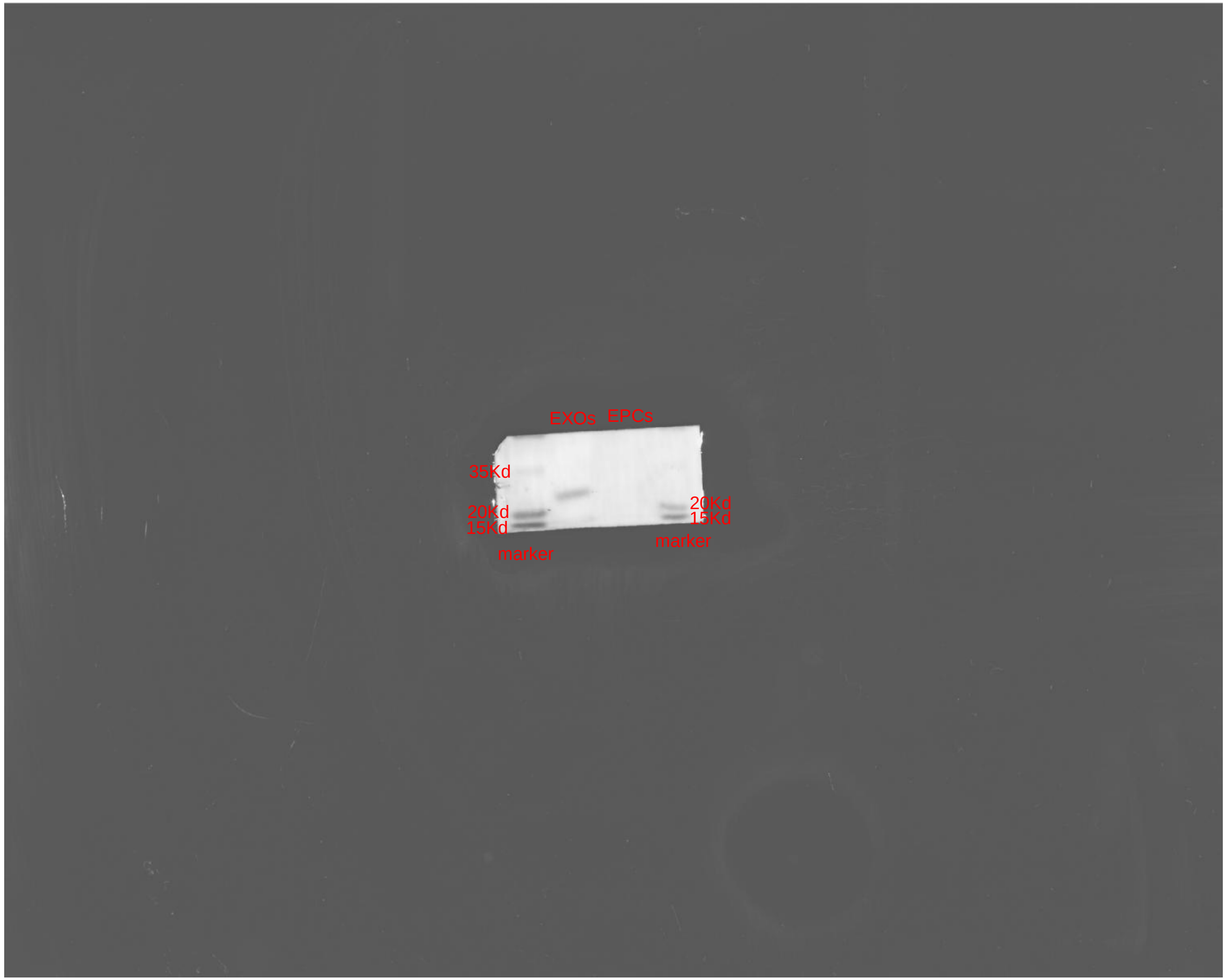

Figure 1D-CD63

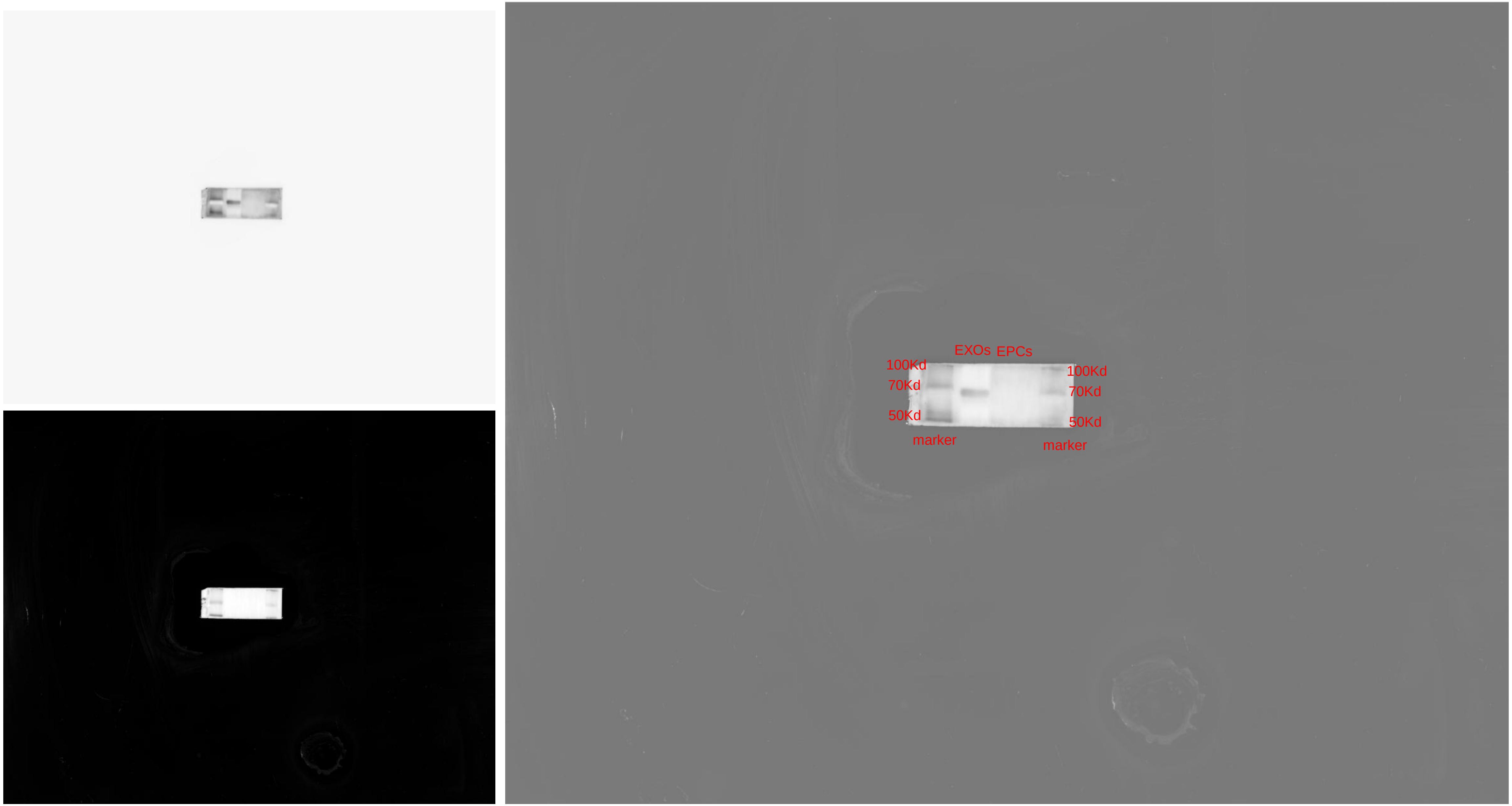

Figure 1D-TSG101

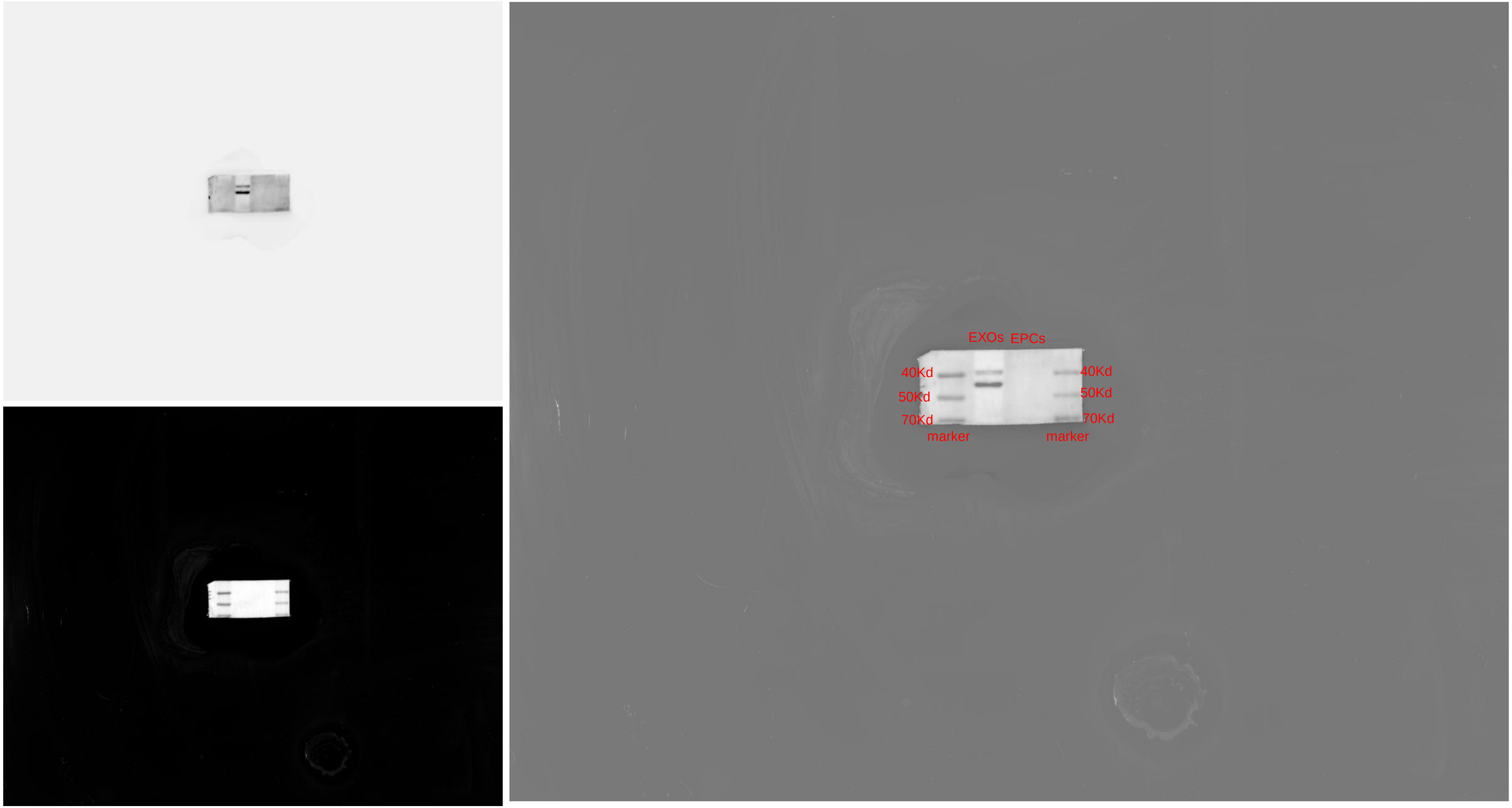

Figure 1D-Calnexin

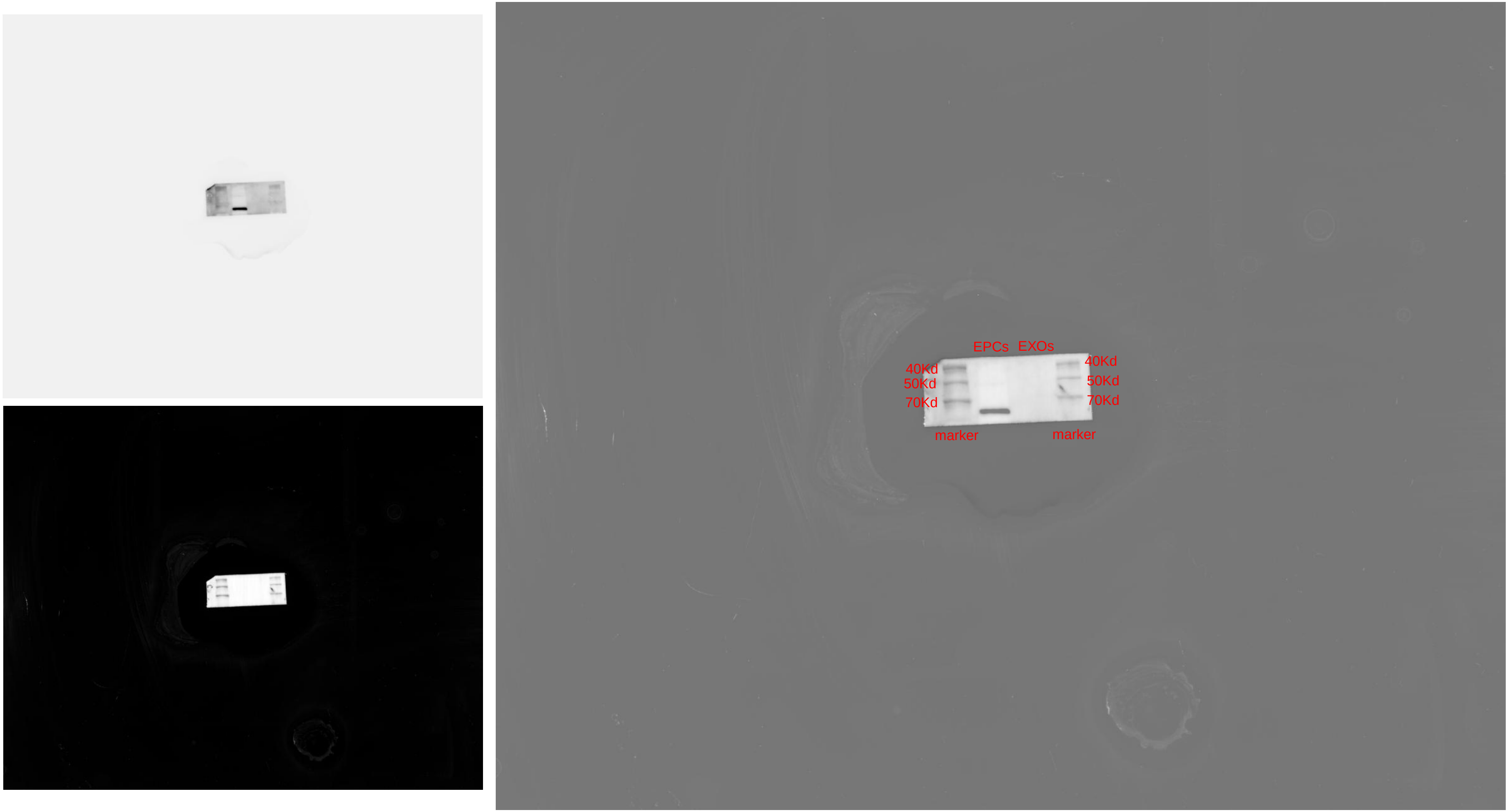

Figure 3C-HSP90

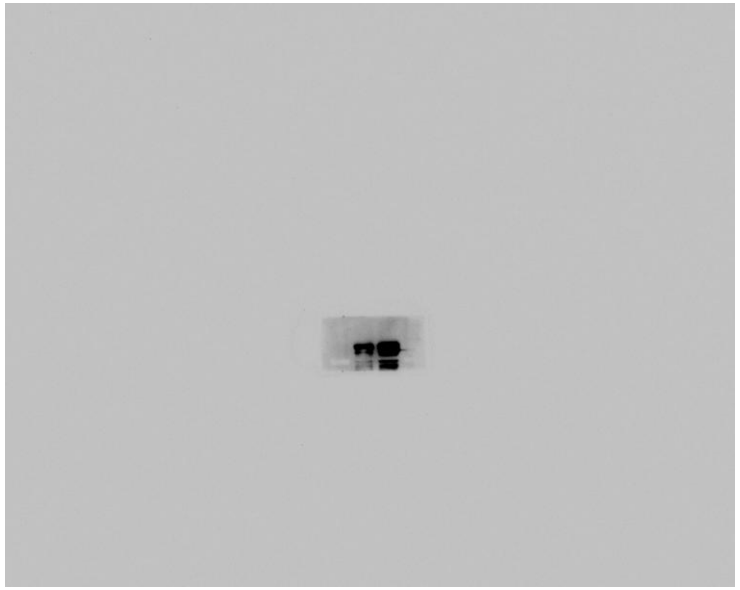

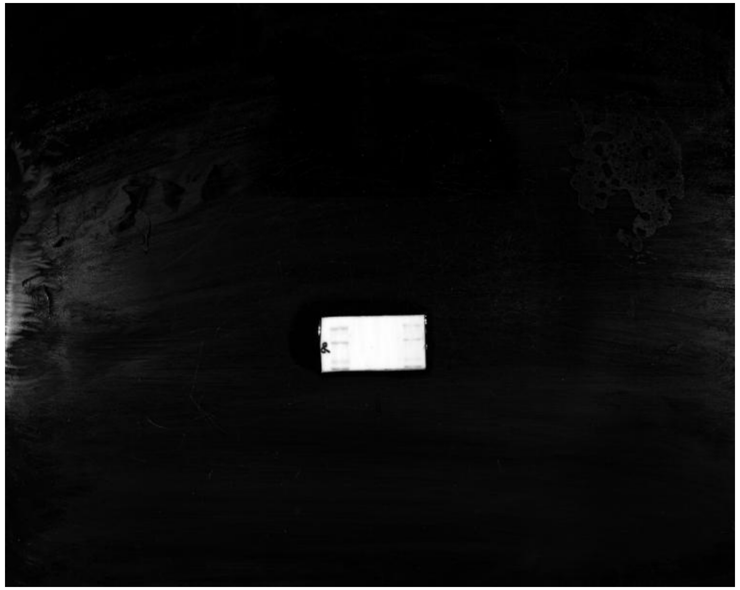

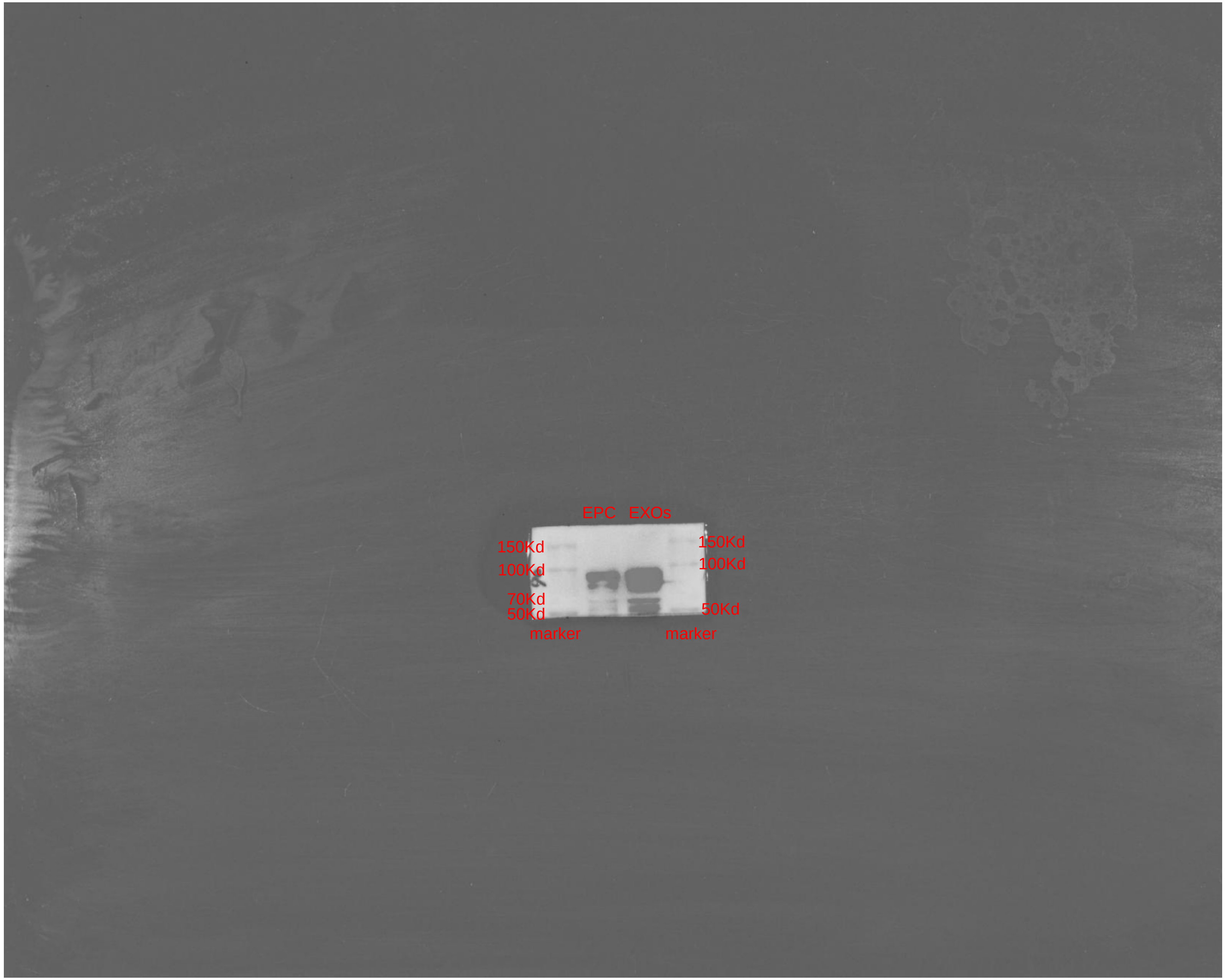

Figure 3D-HSP90

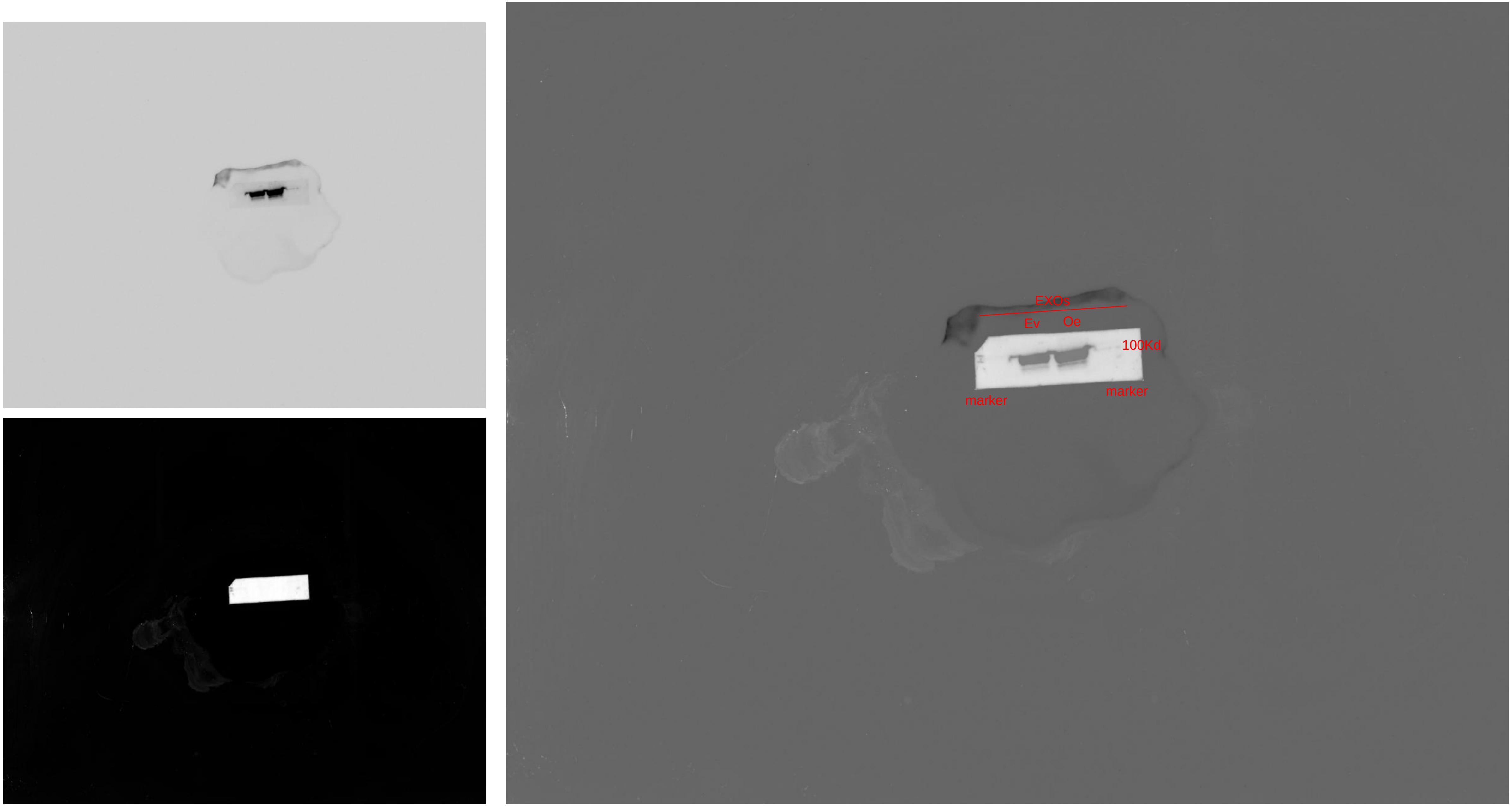

Figure 3D-TSG101

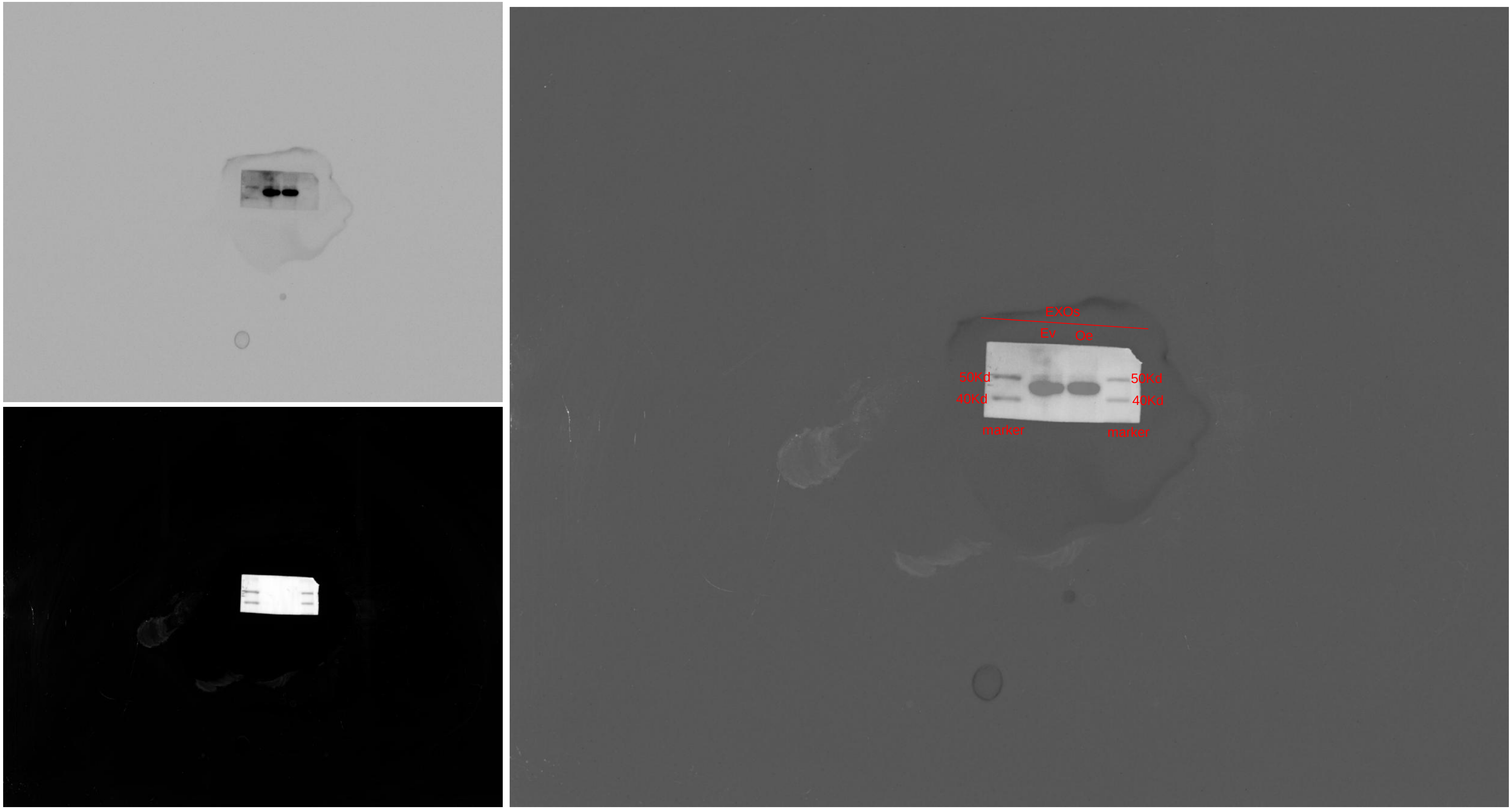

Figure 3E-HSP90

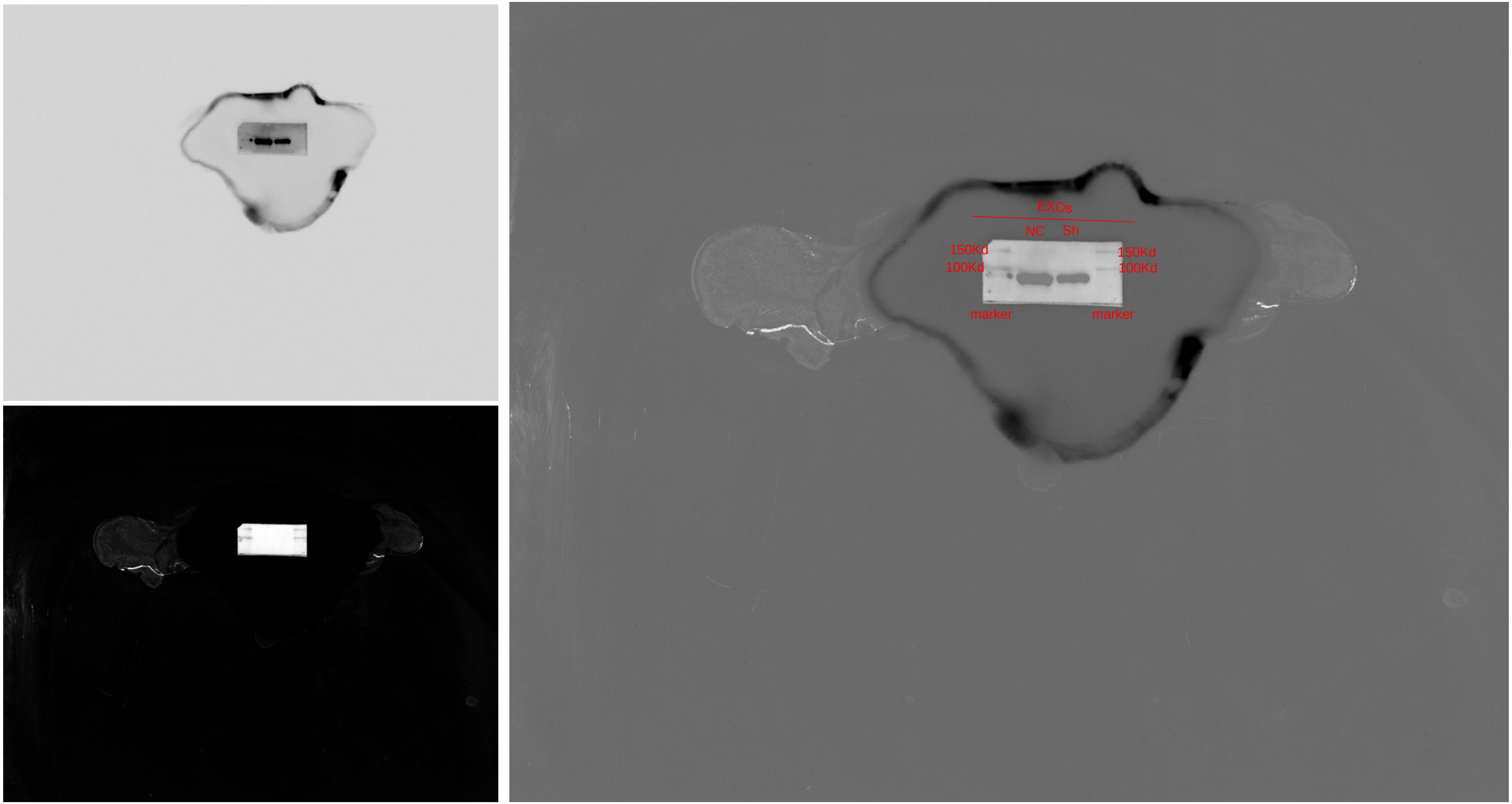

Figure 3E-TSG101

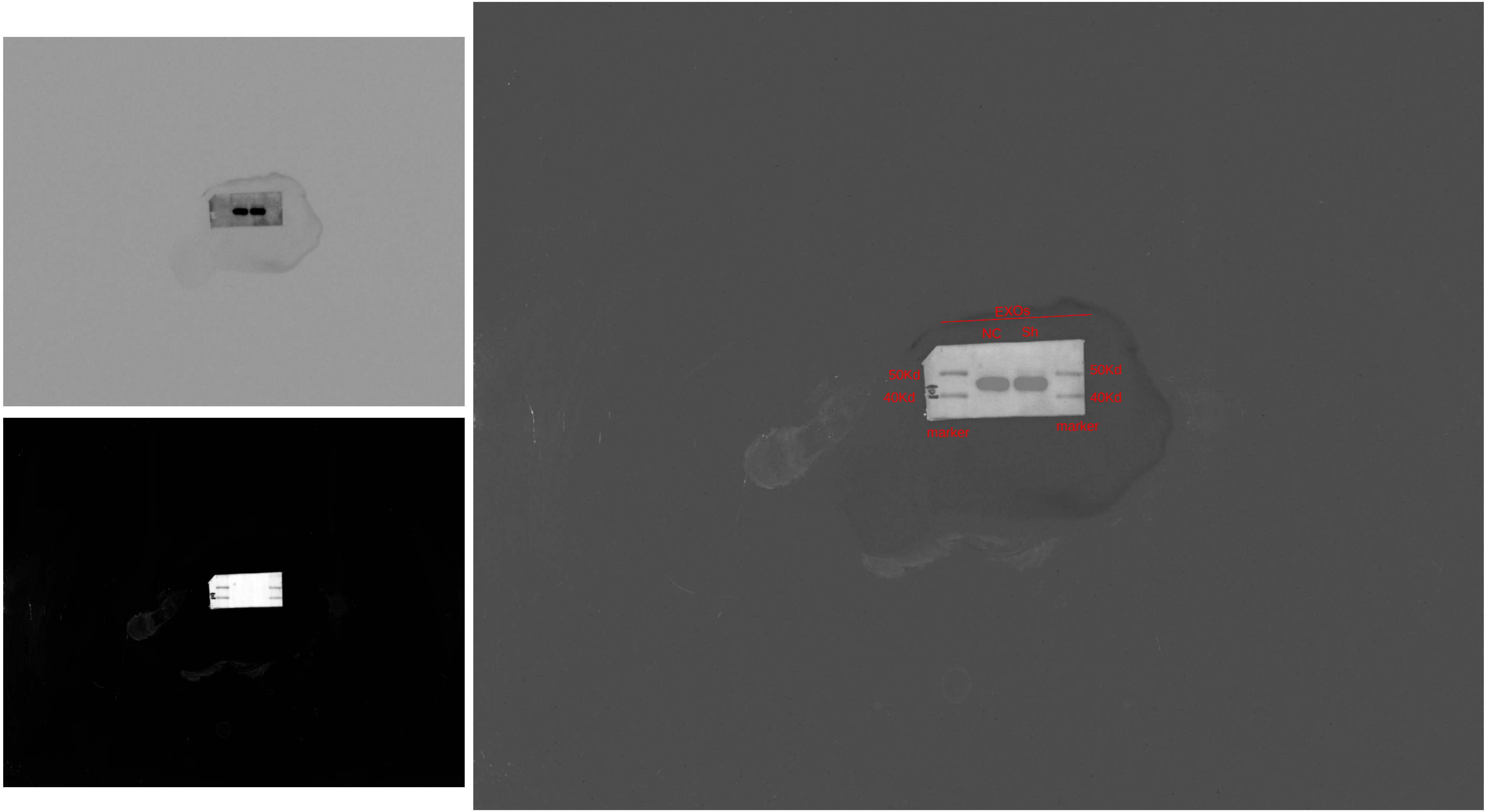

Figure 4A-p-AKT

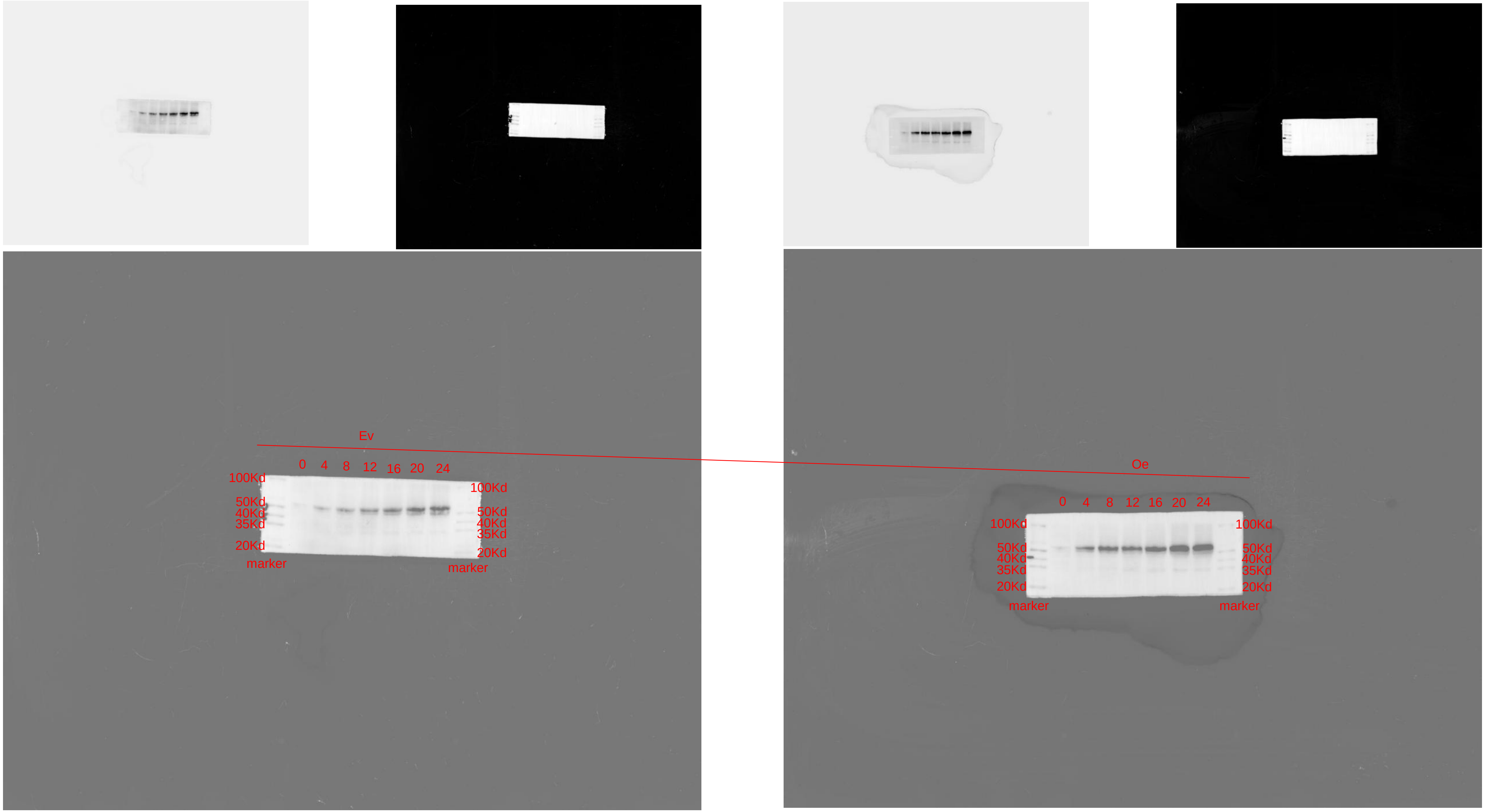

Figure 4A-AKT

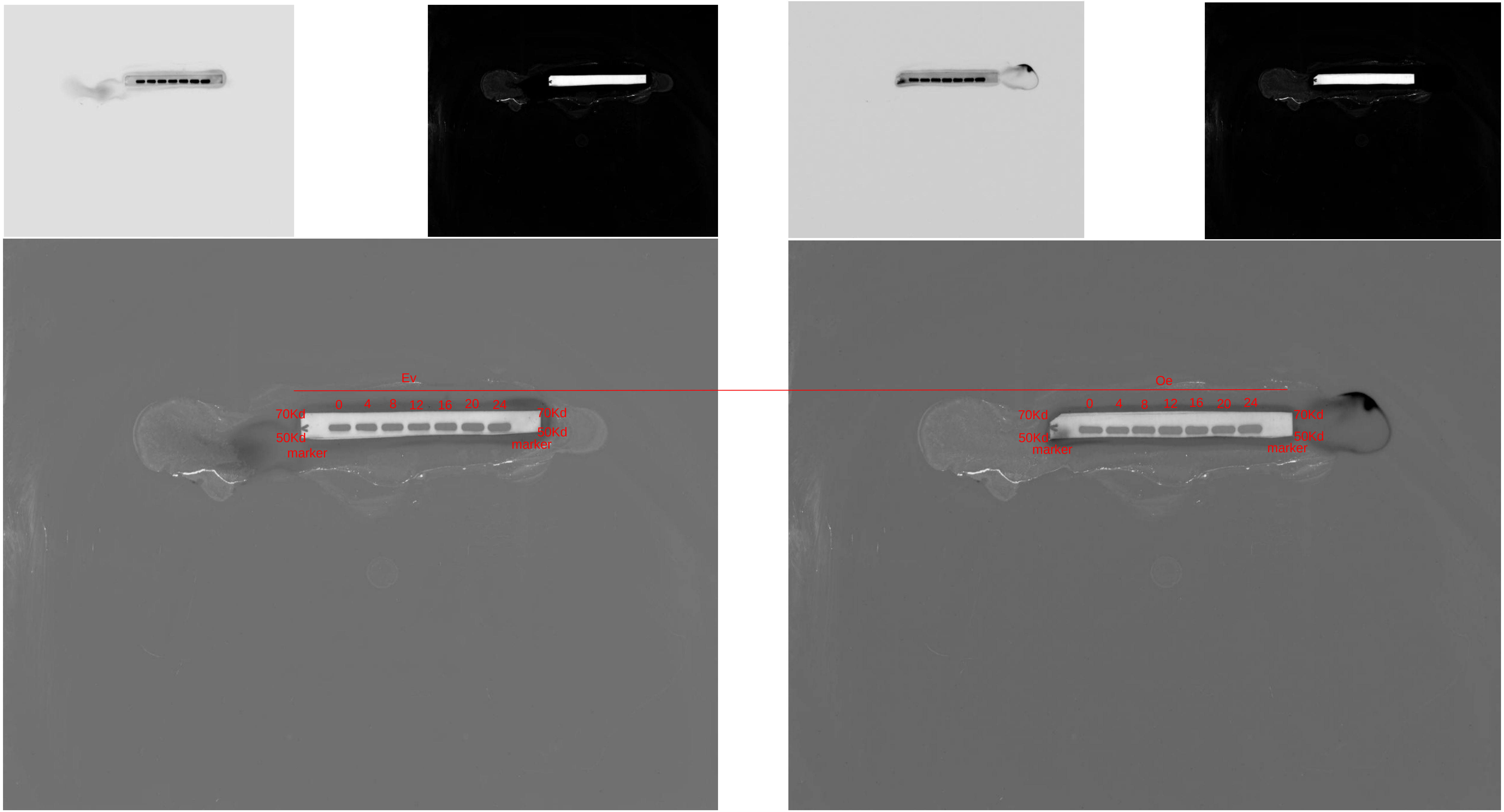

Figure 4A-p-PI3K

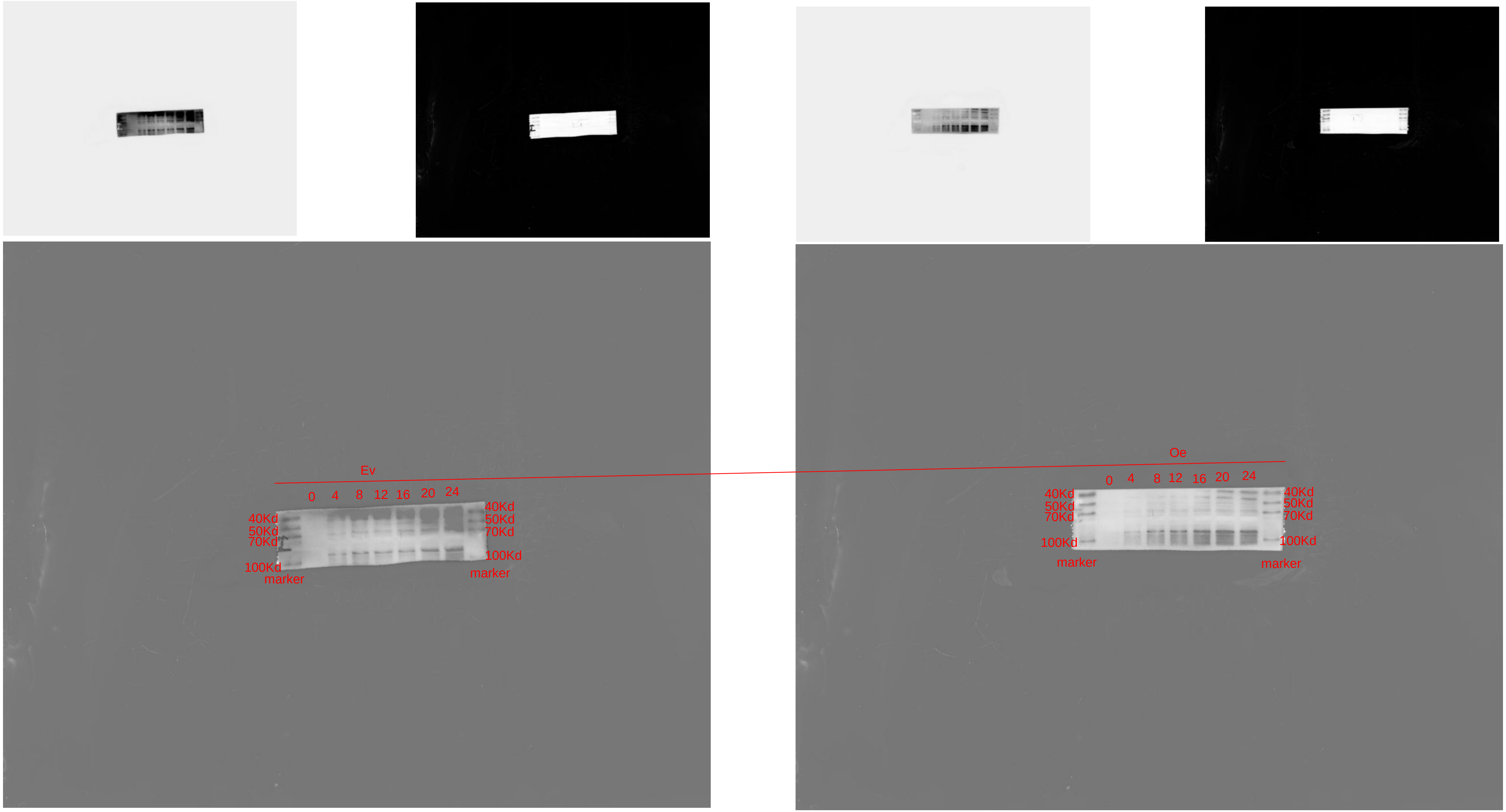

Figure 4A-PI3K

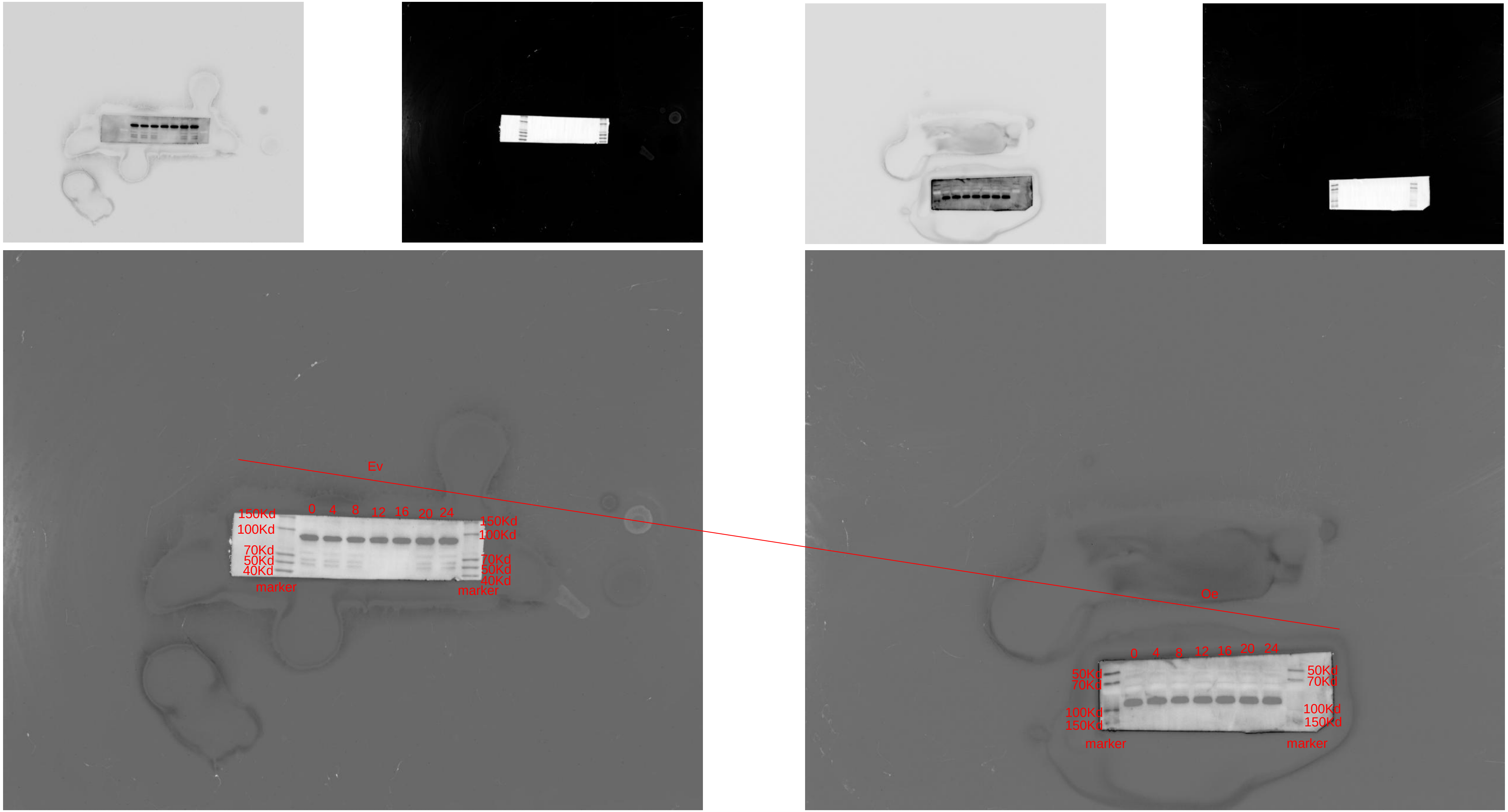

Figure 4A-β-actin

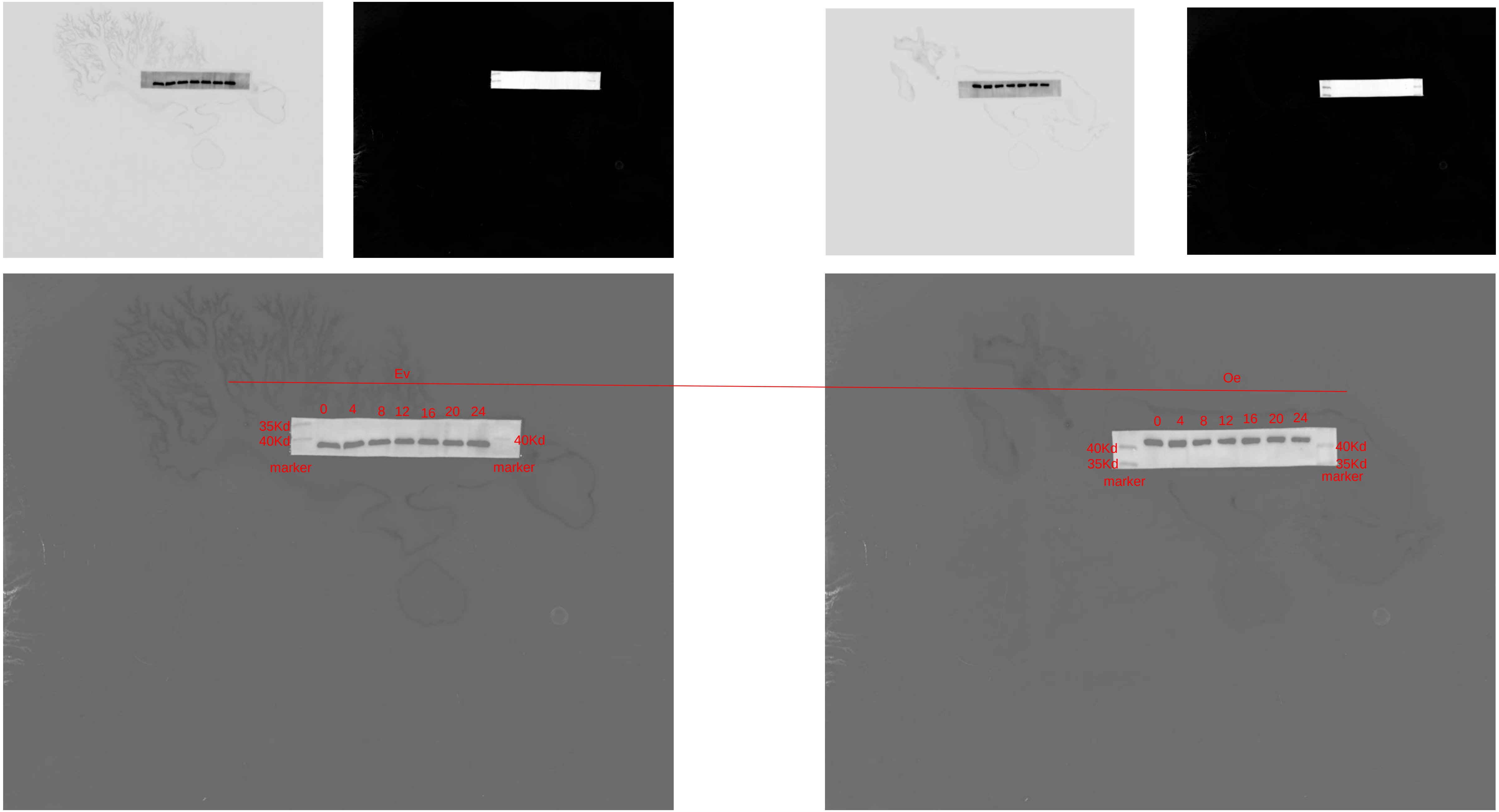

Figure 4C-p-AKT

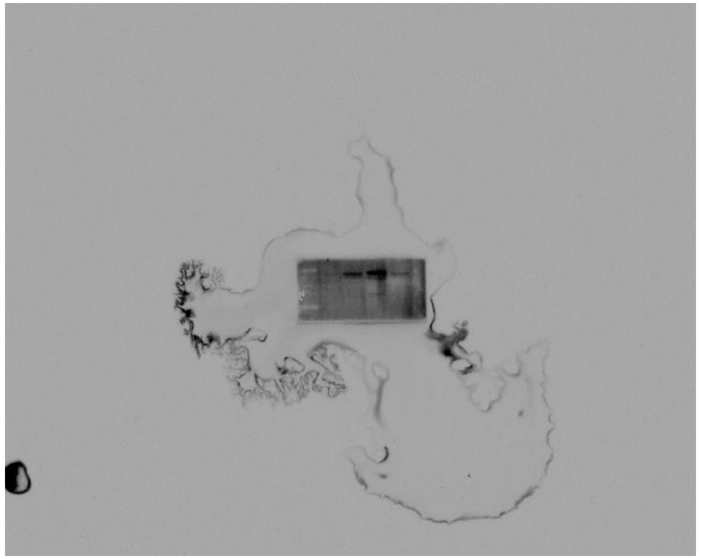

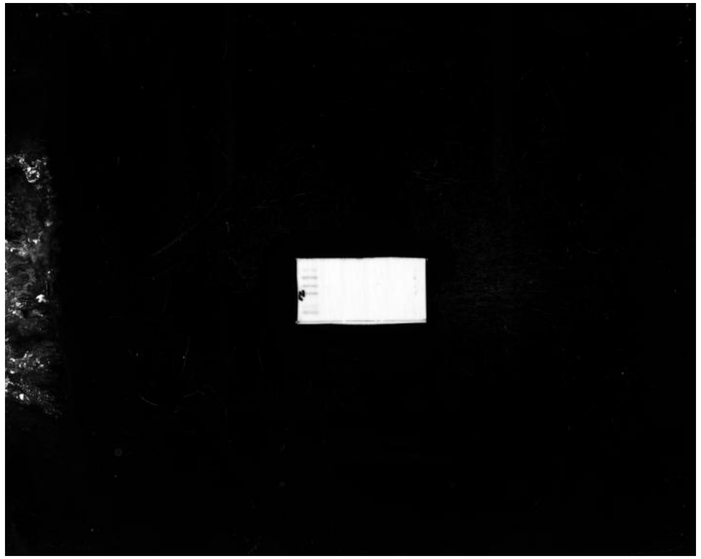

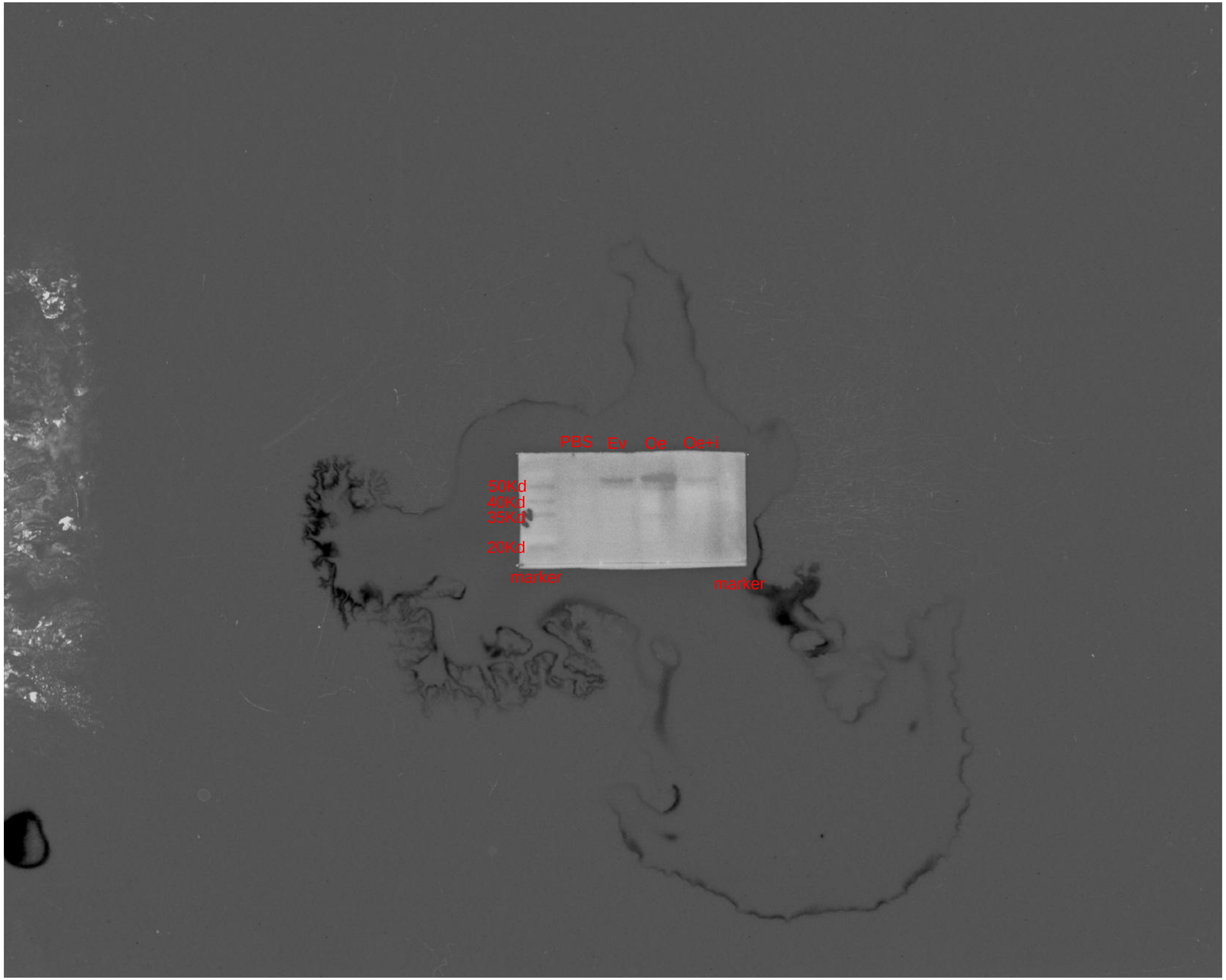

Figure 4C-AKT

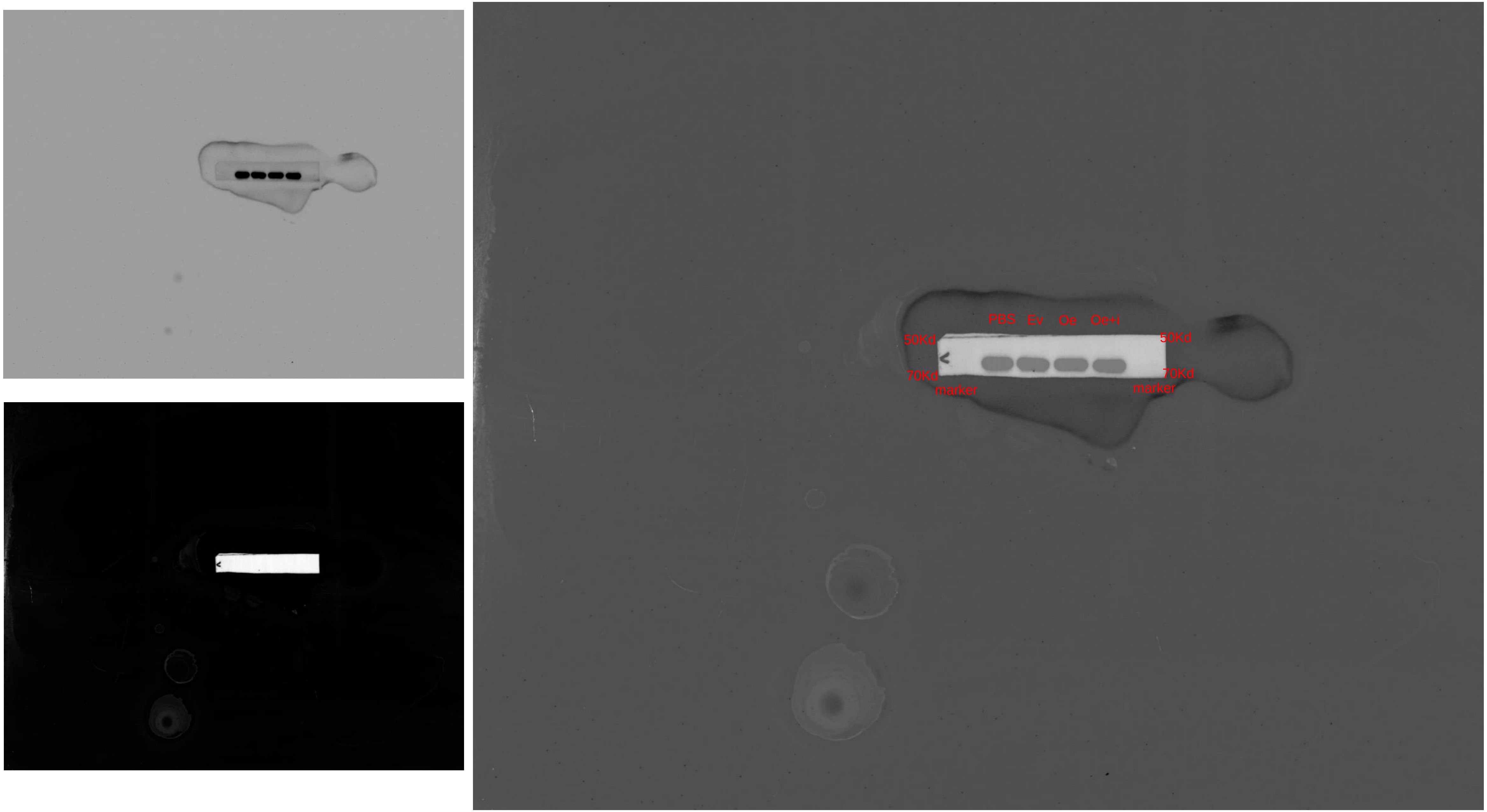

Figure 4C-β-actin

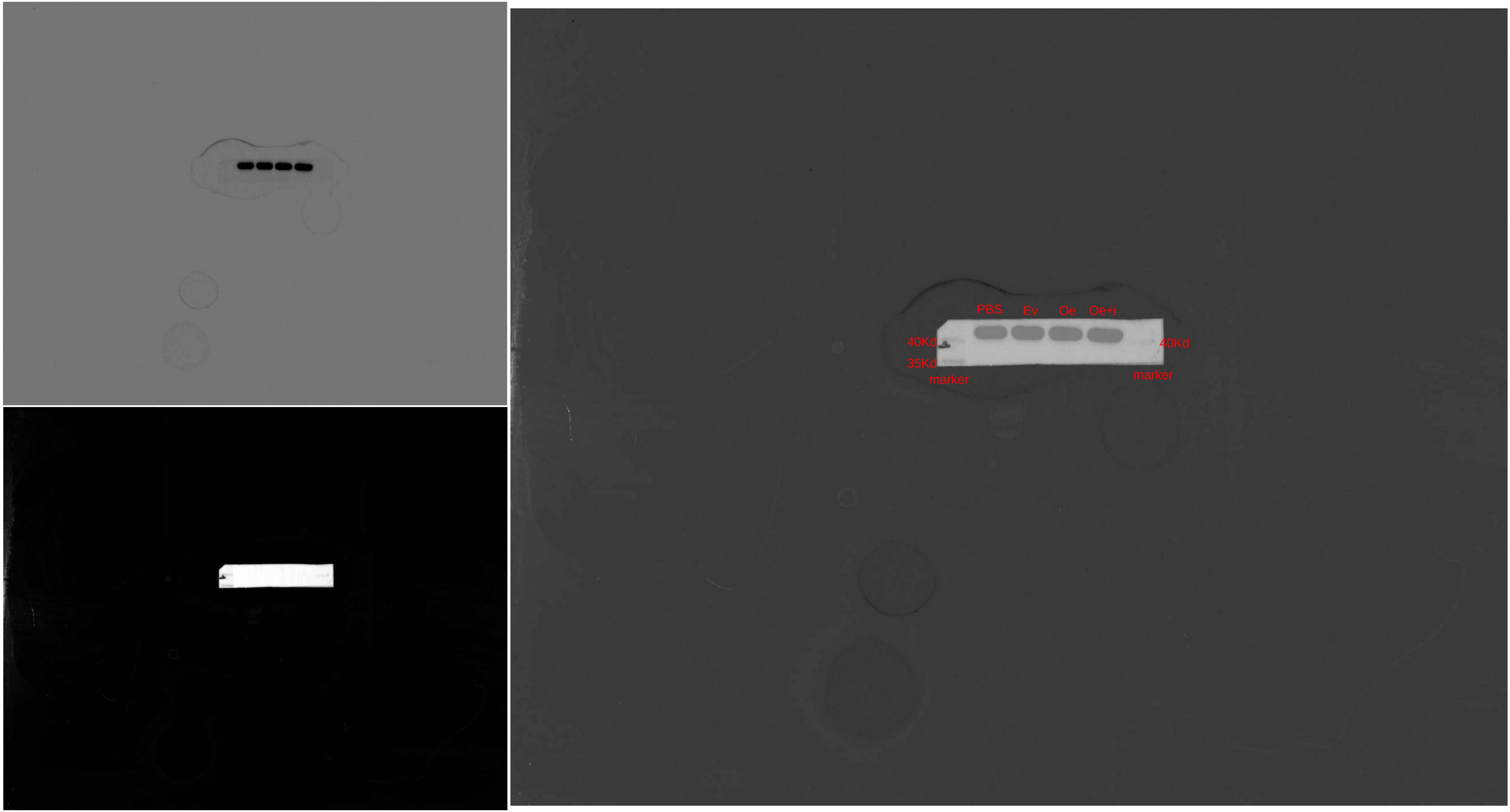

Figure 5J-CD9

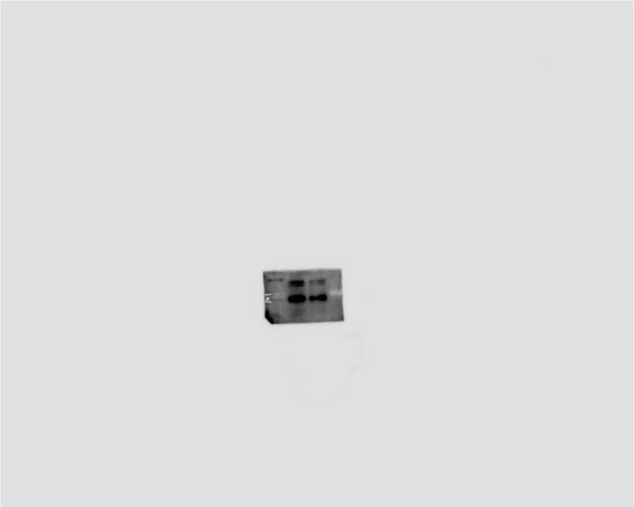

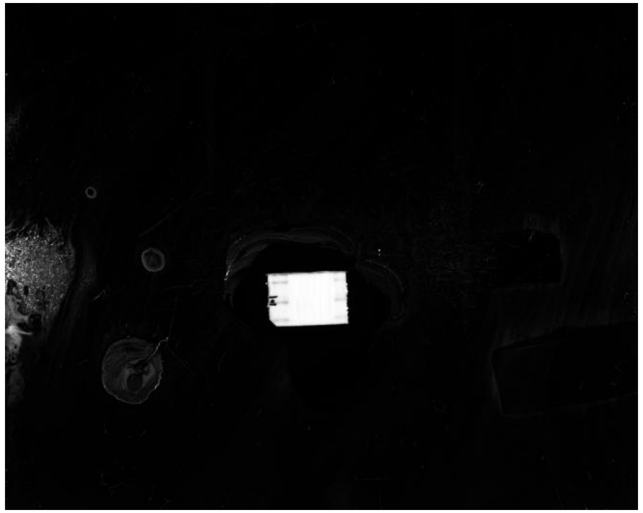

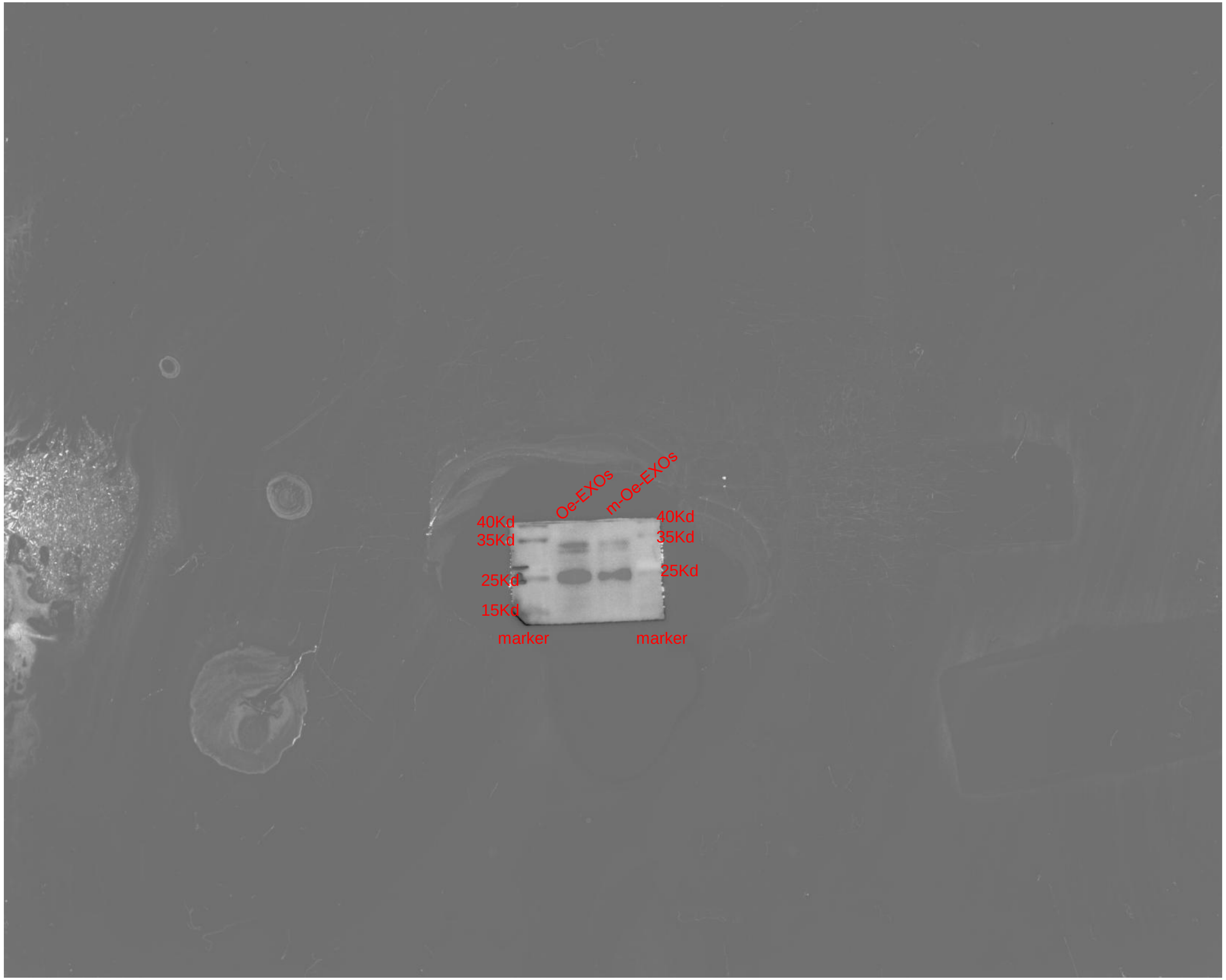

Figure 5J-CD63

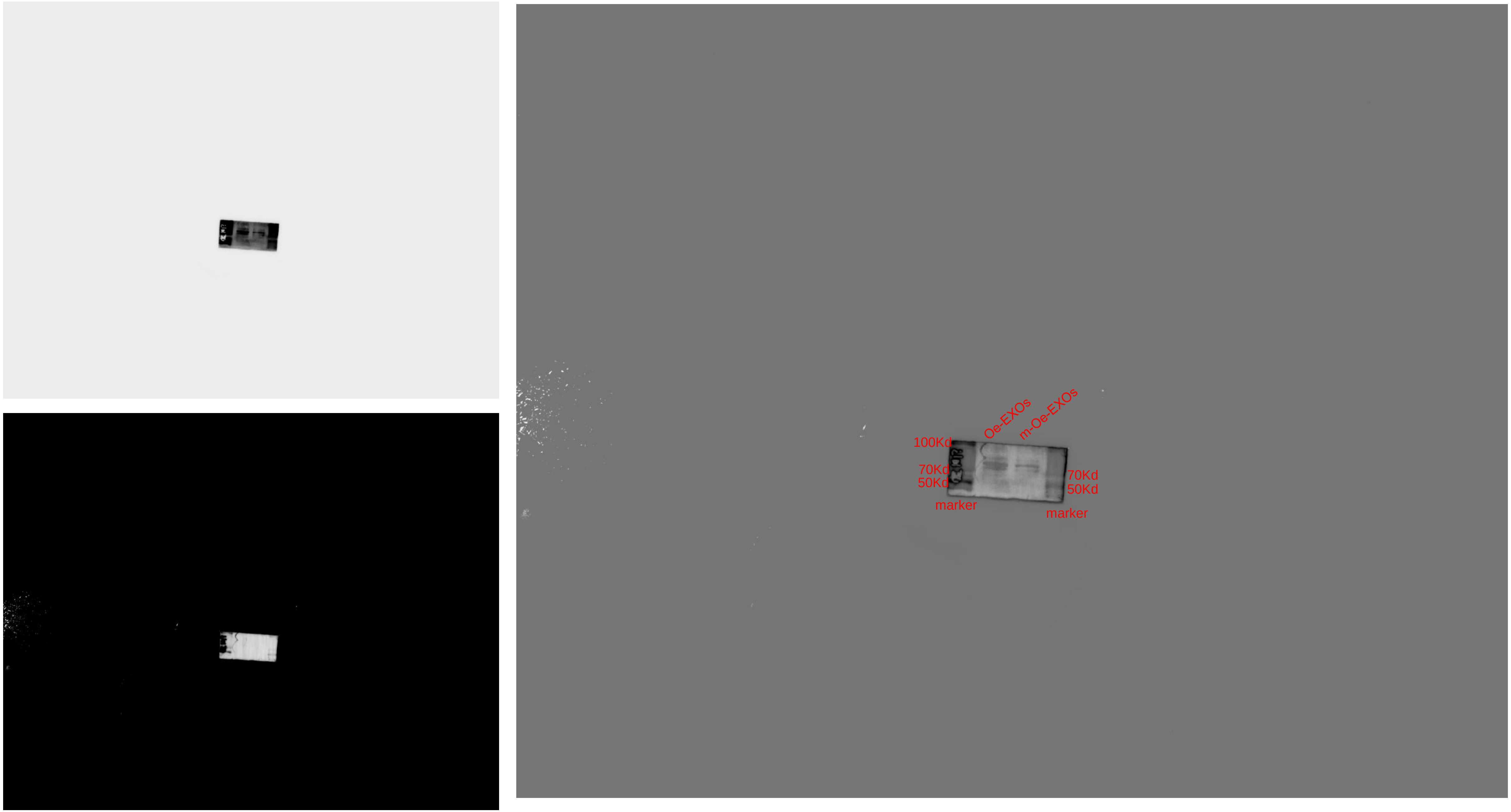

Figure 5J-TSG101

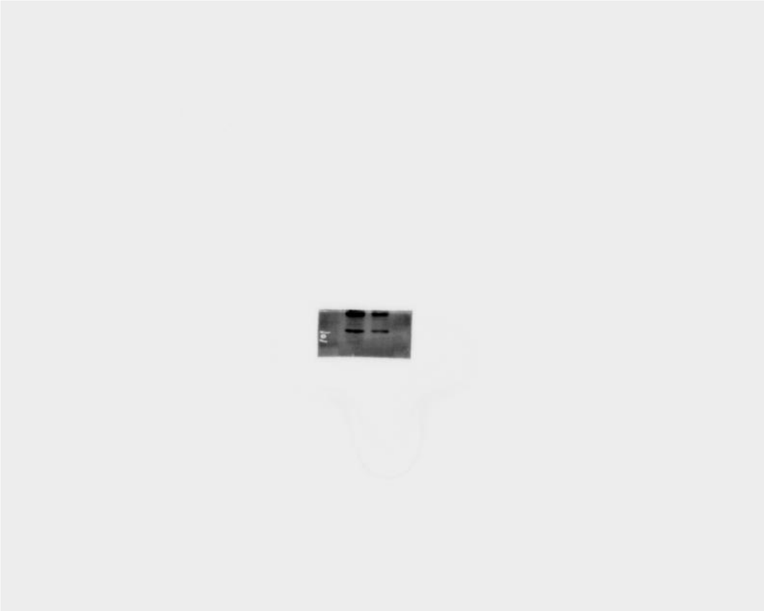

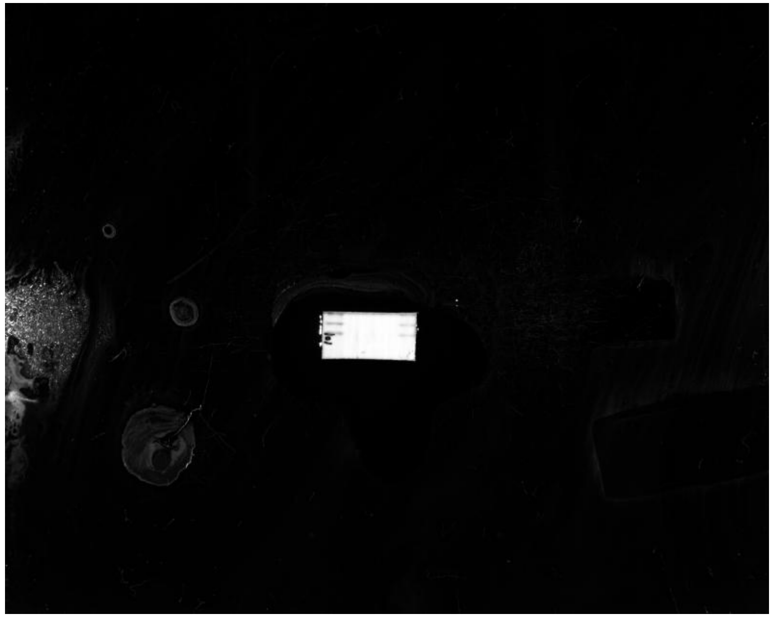

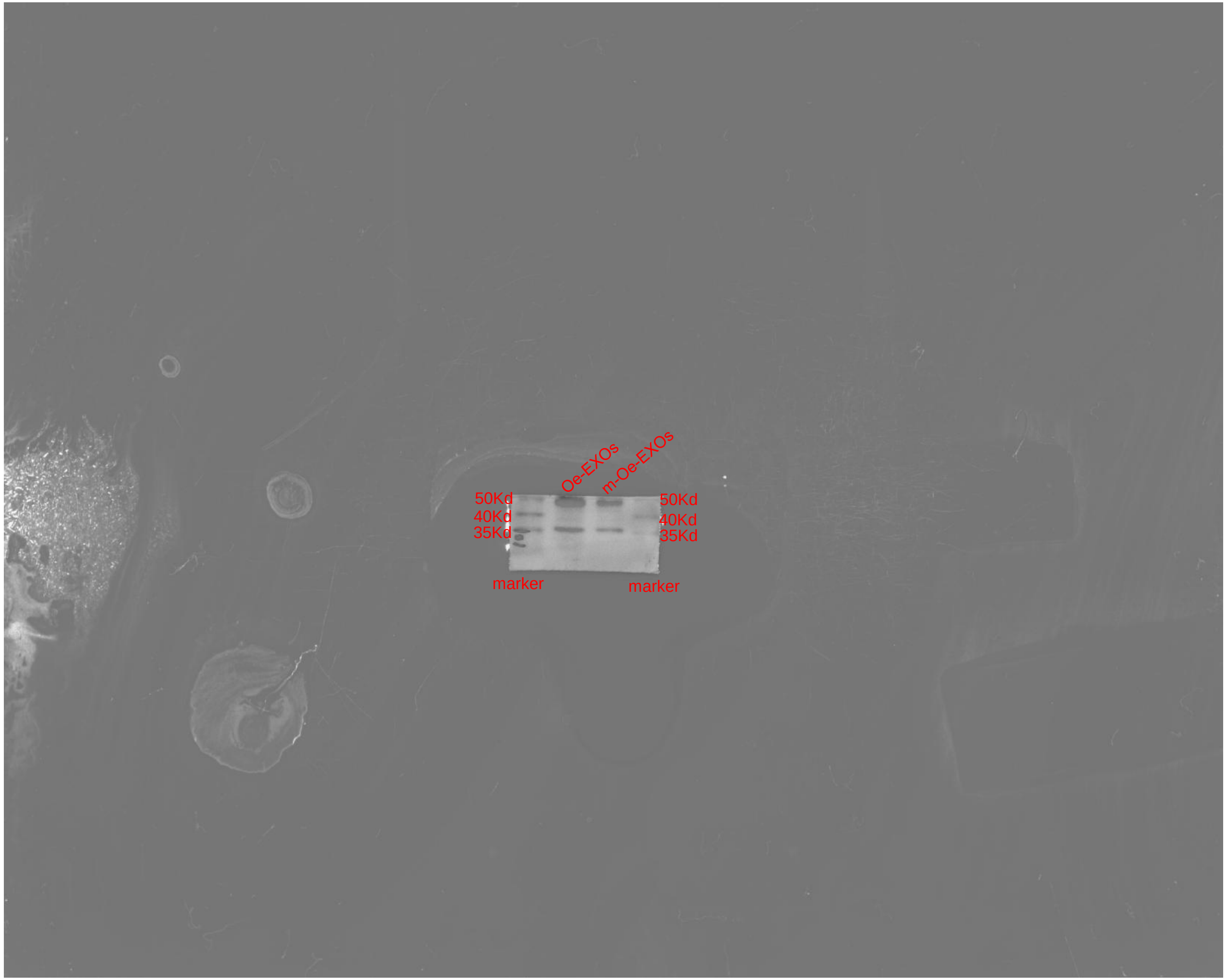

Figure 5J-CD47

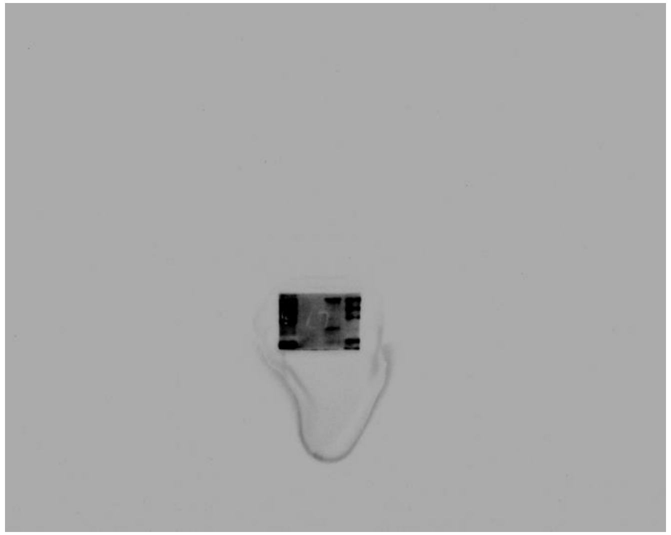

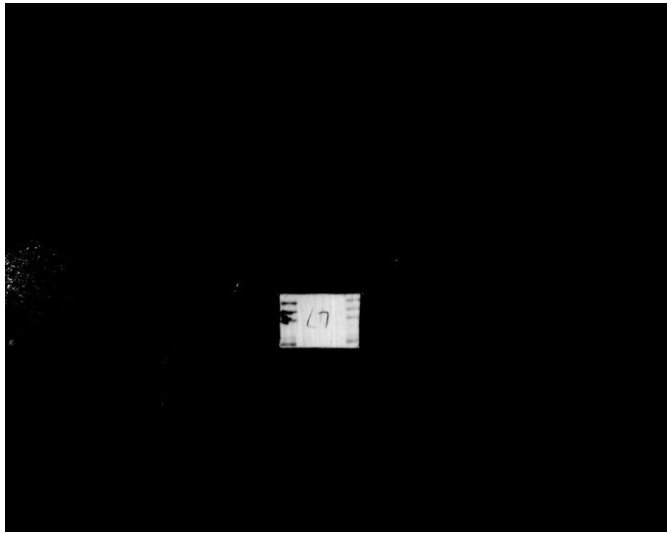

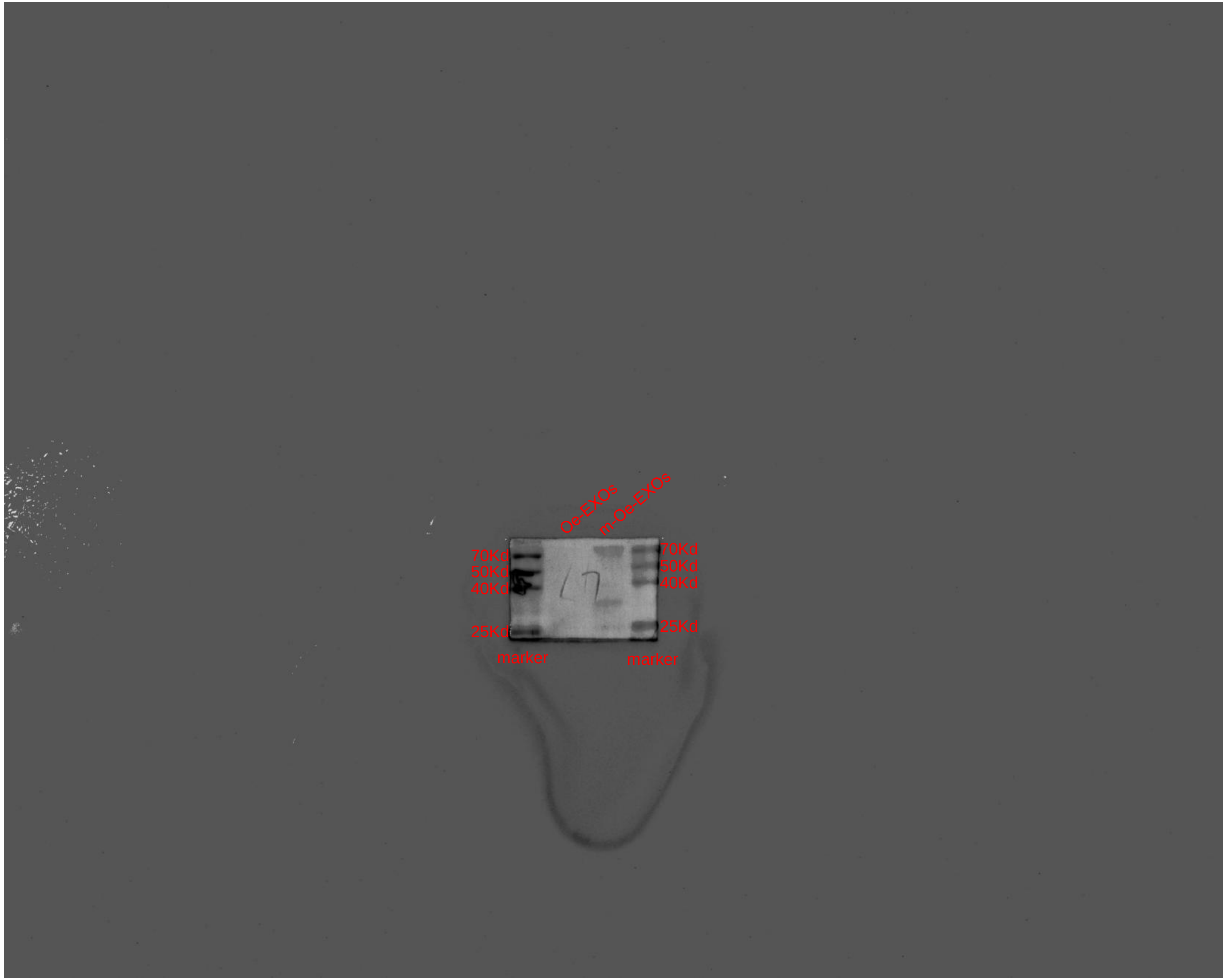

Figure 6G-Claudin-5

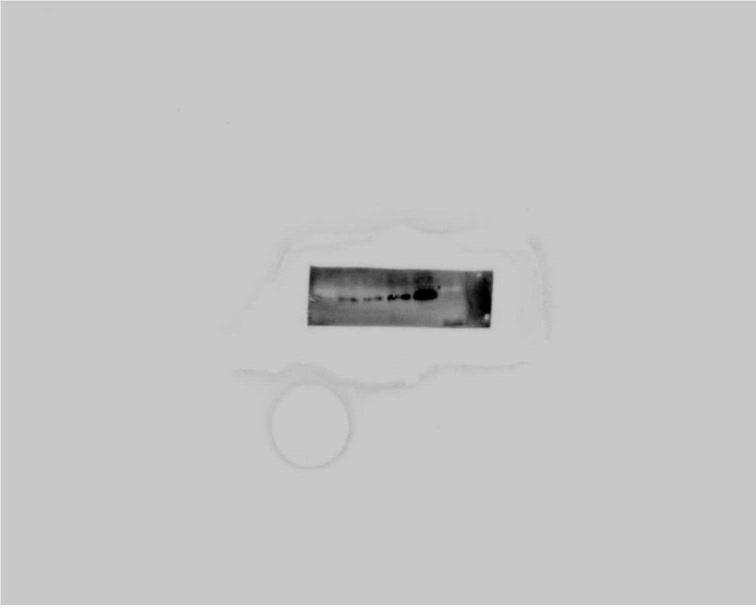

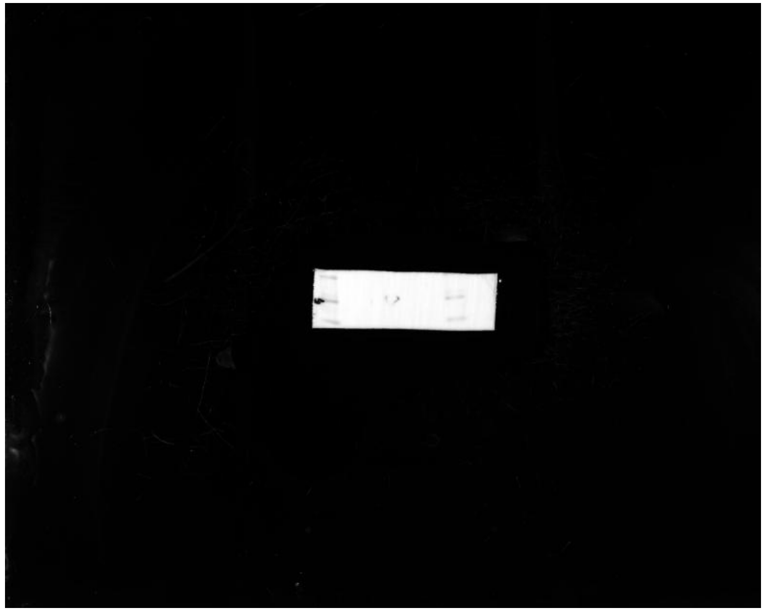

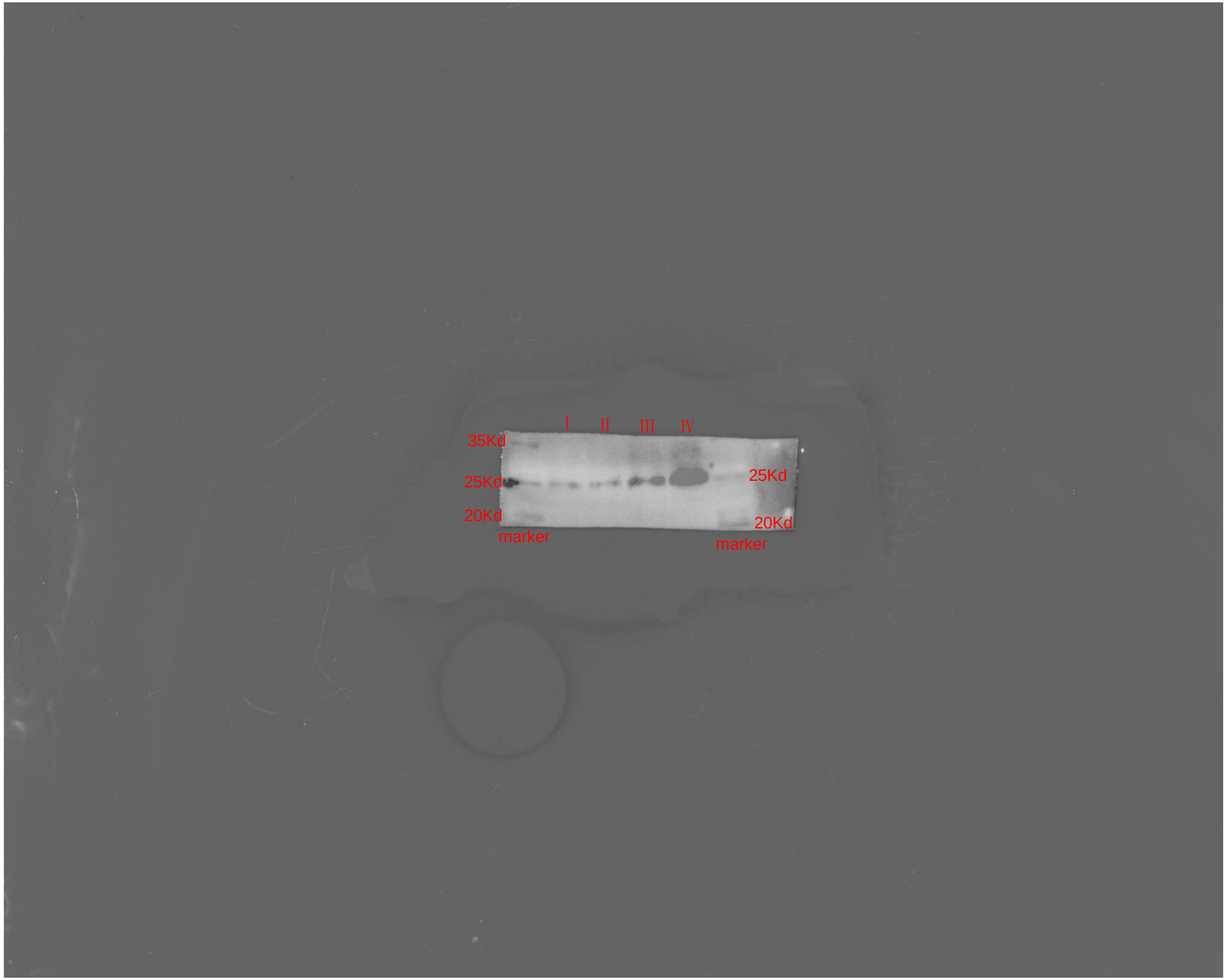

Figure 6G-ZO-3

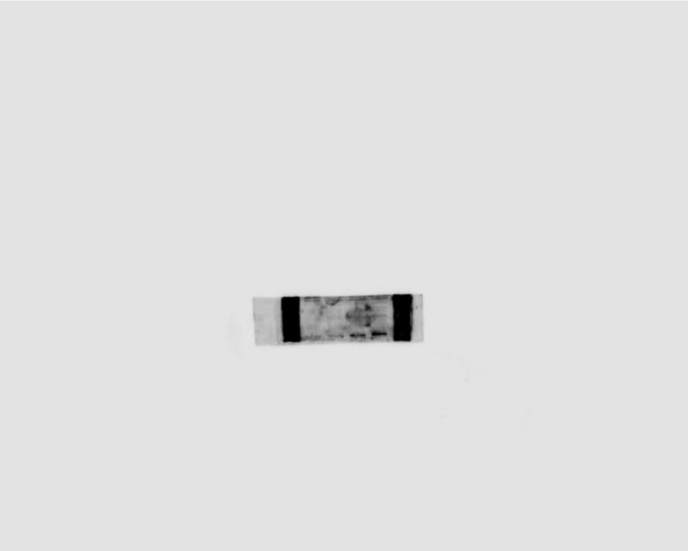

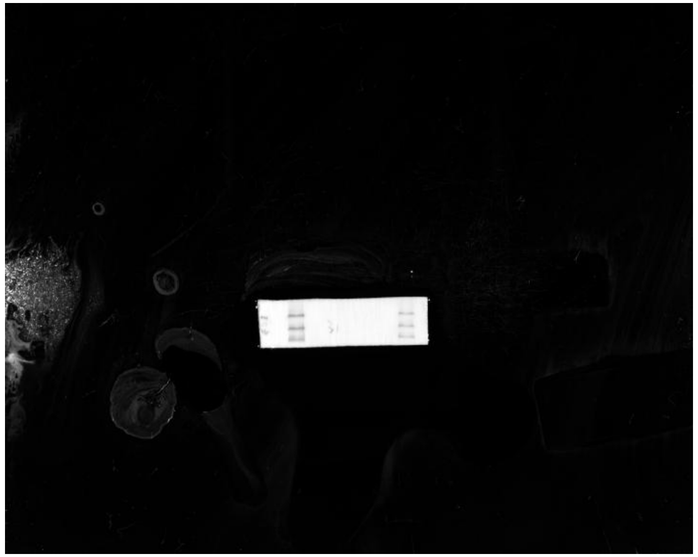

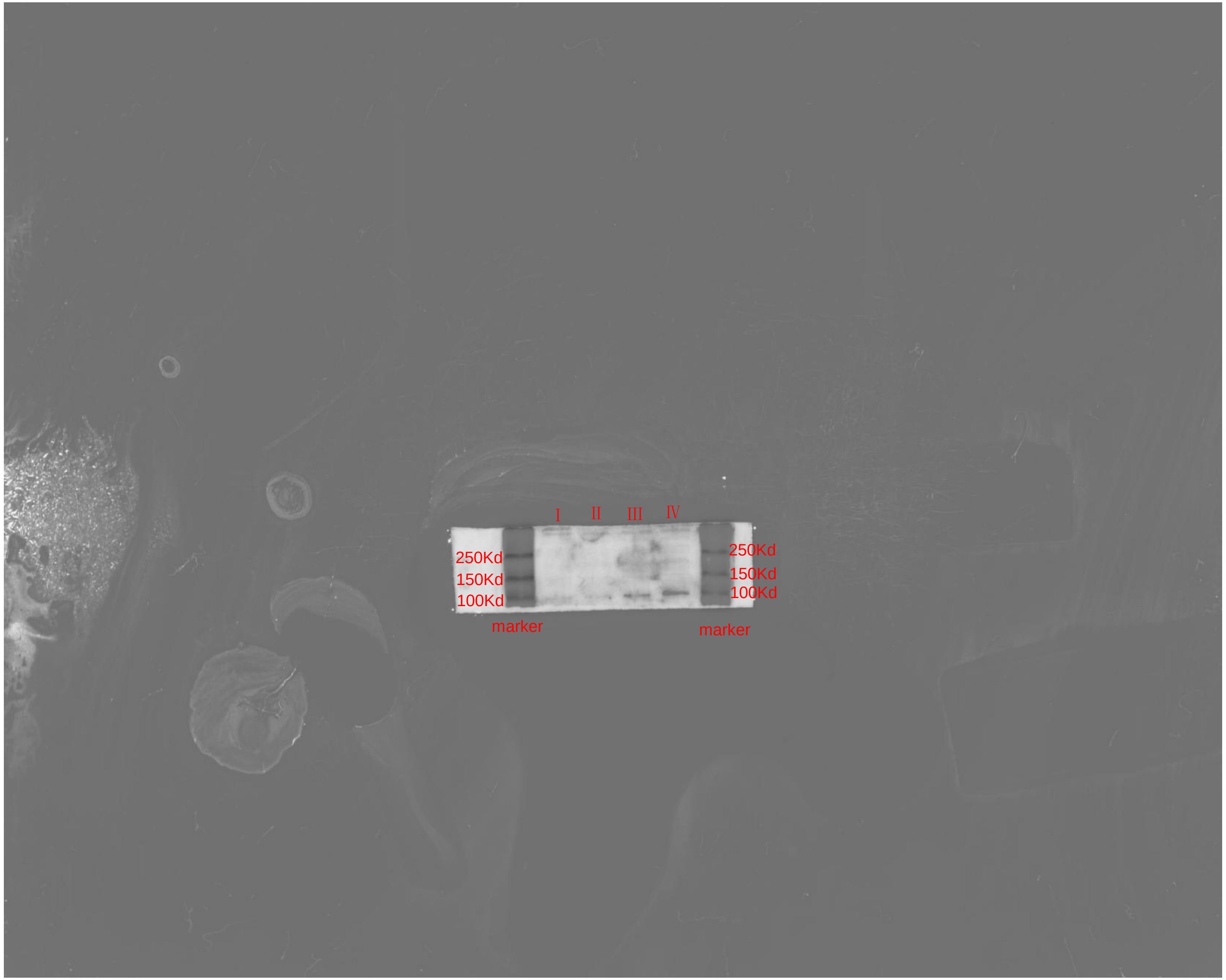

Figure 6G-Occludin

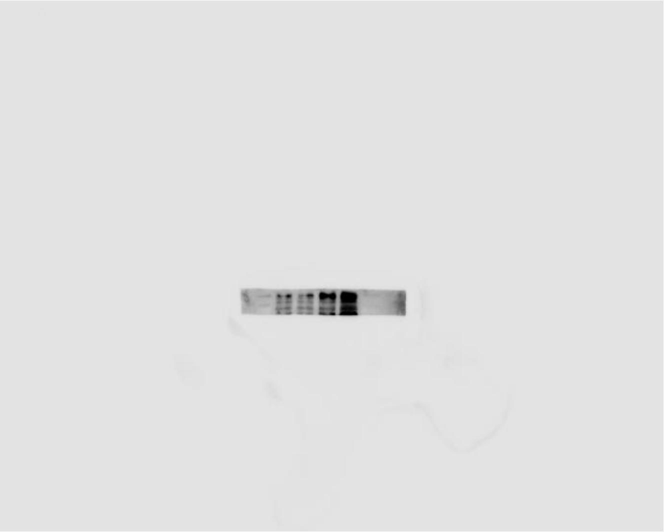

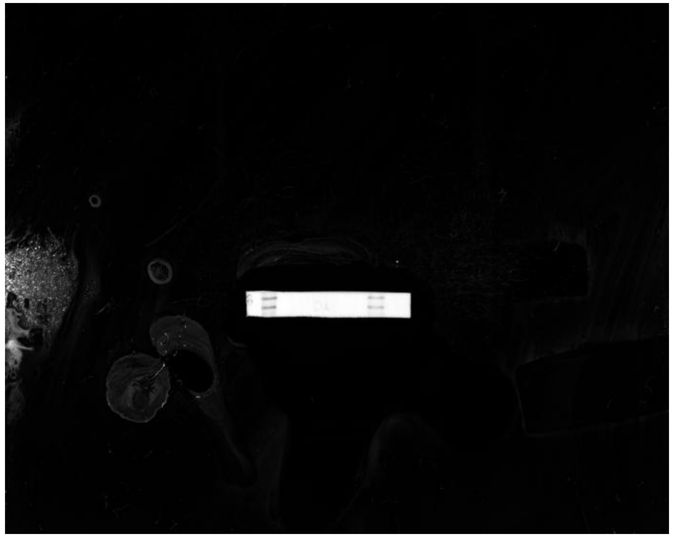

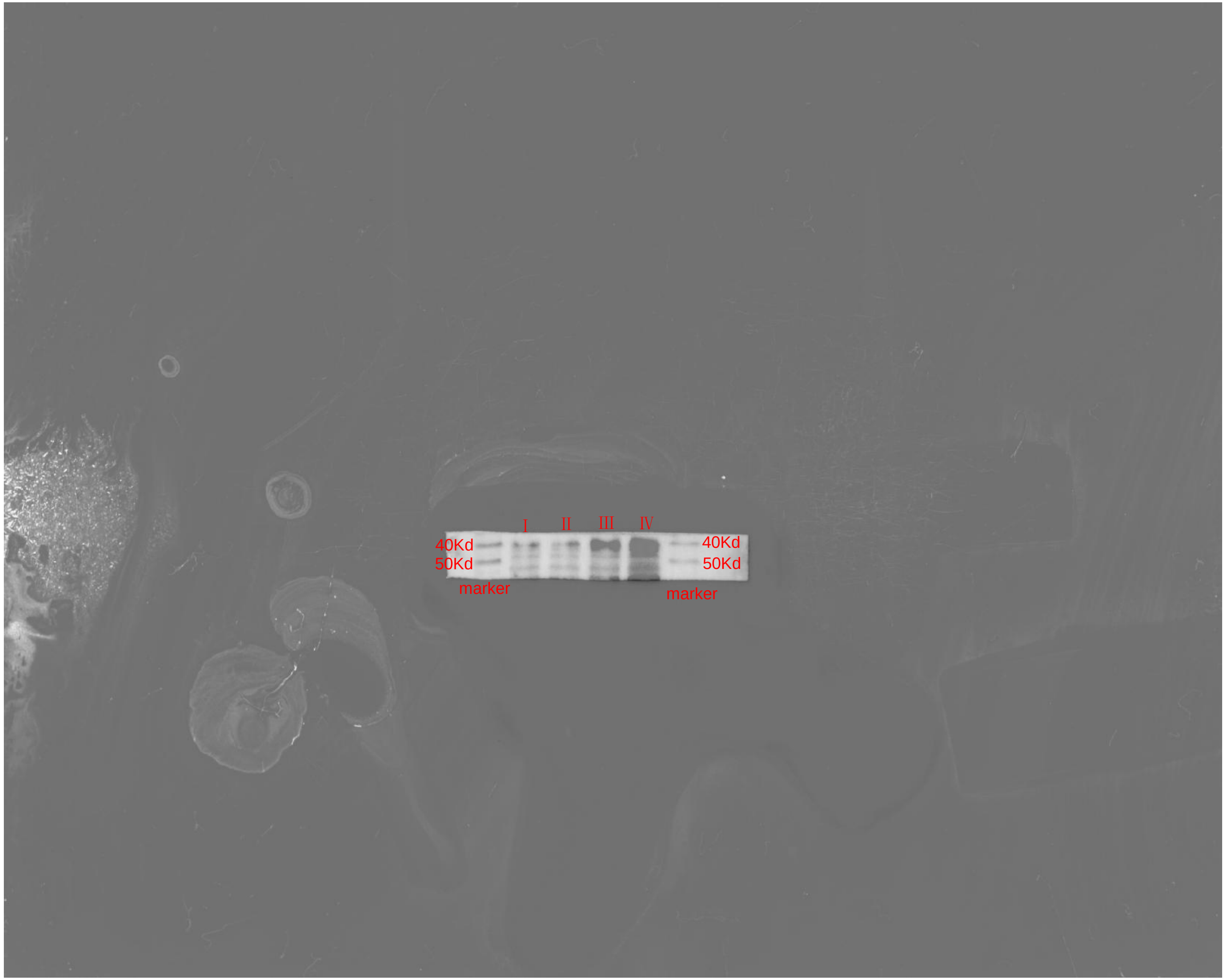

Figure 6G-ZO-2

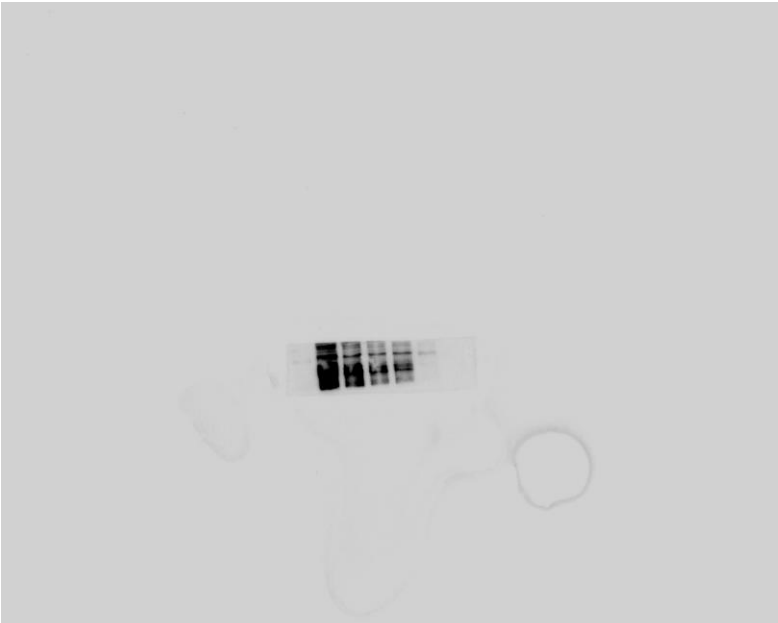

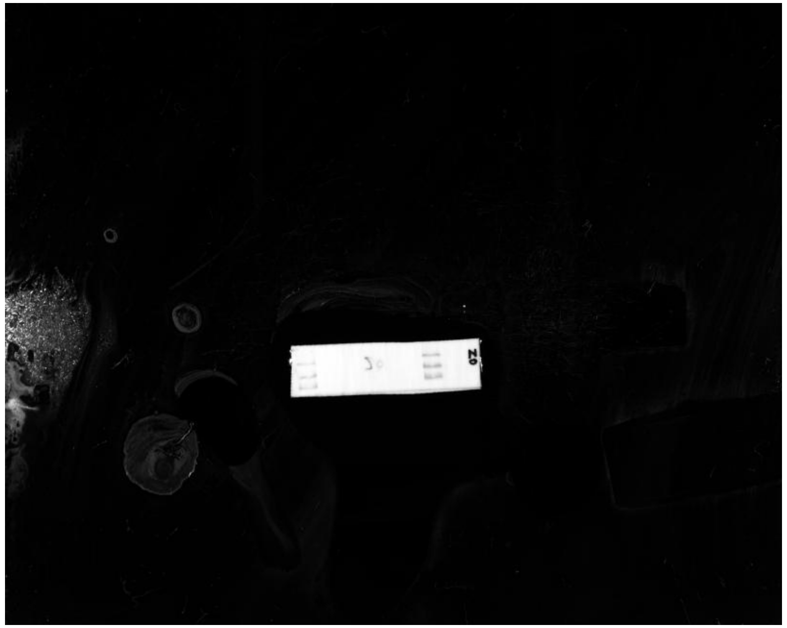

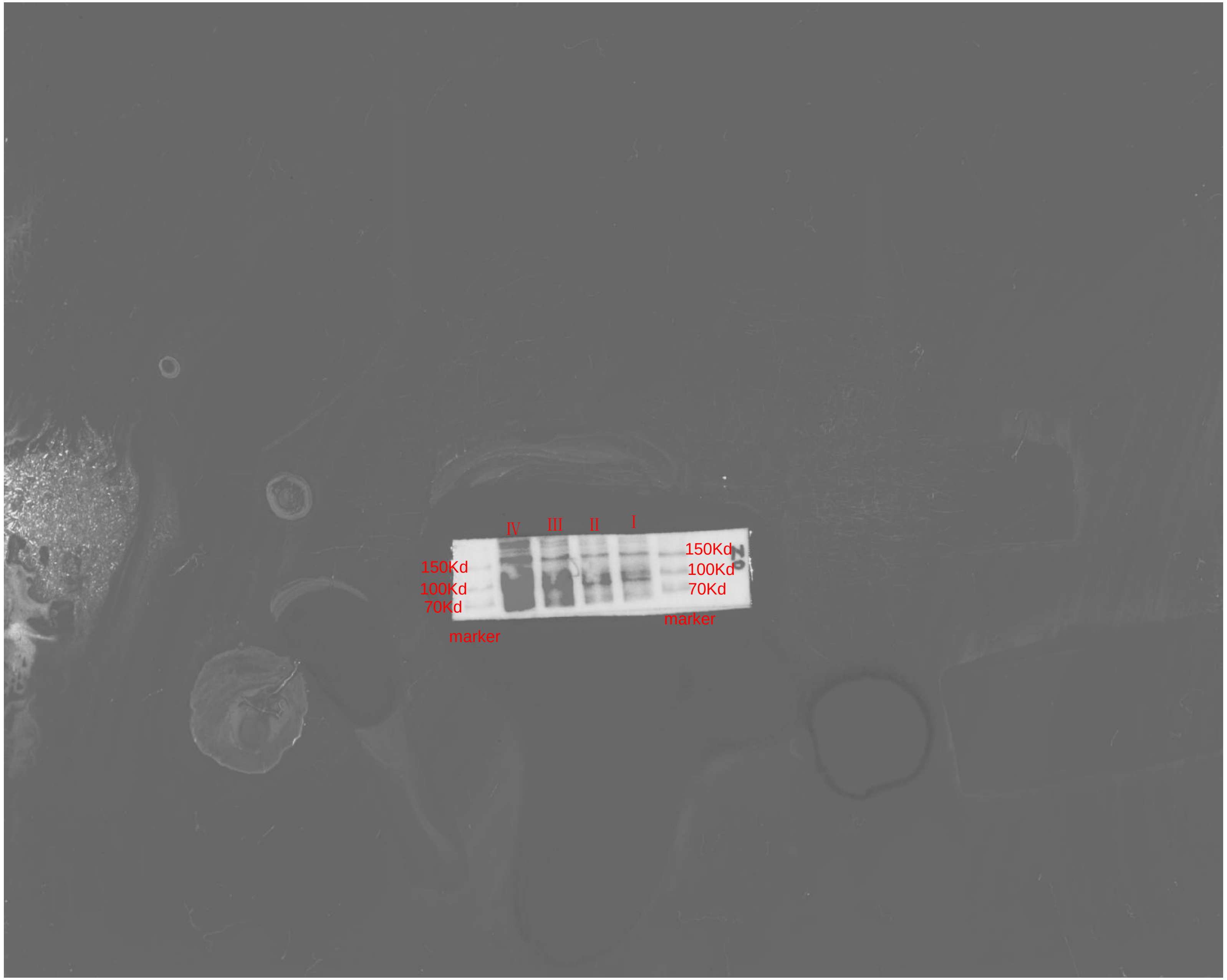

Figure 6G-β-actin

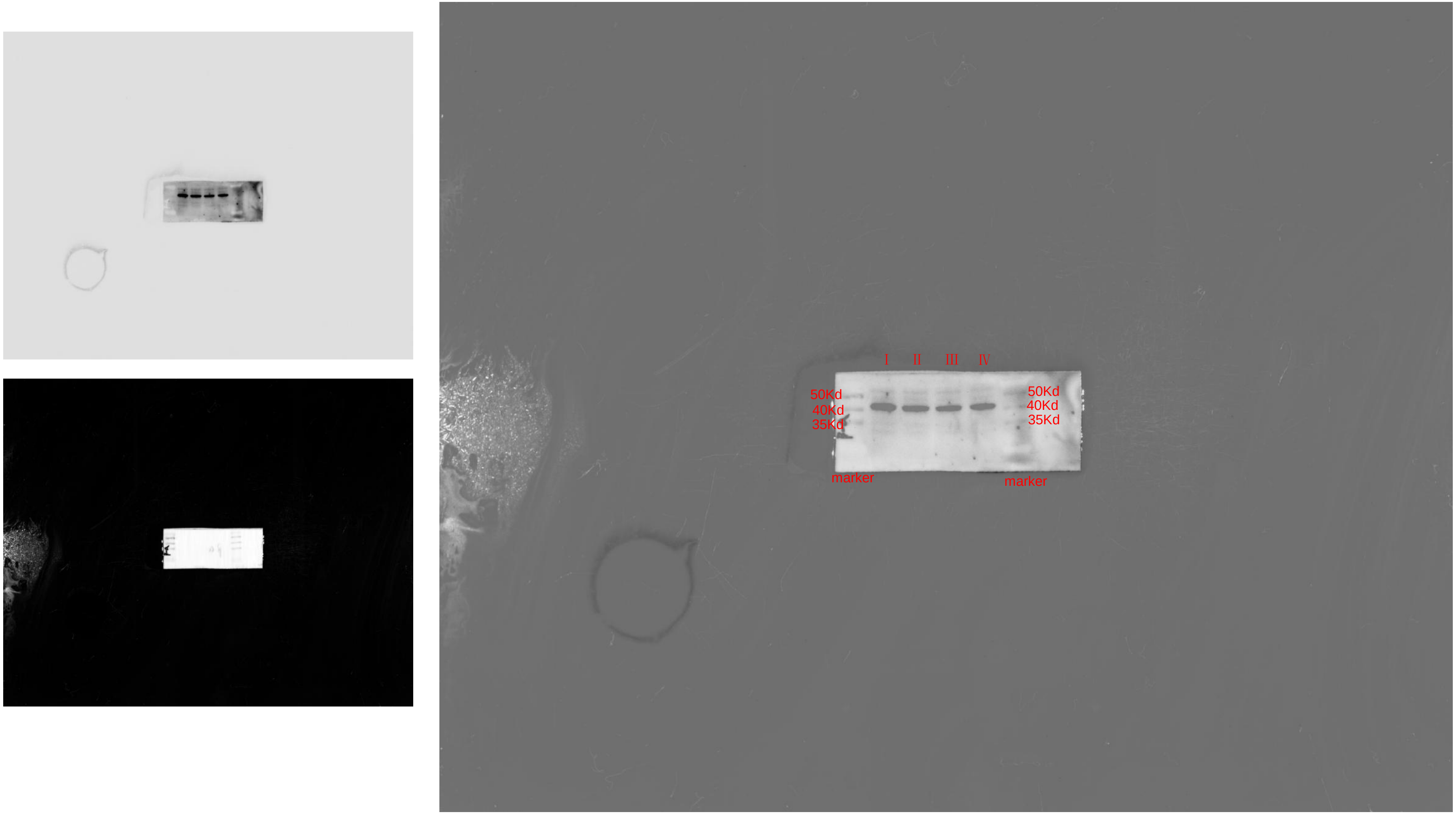

Figure. S6-CD9

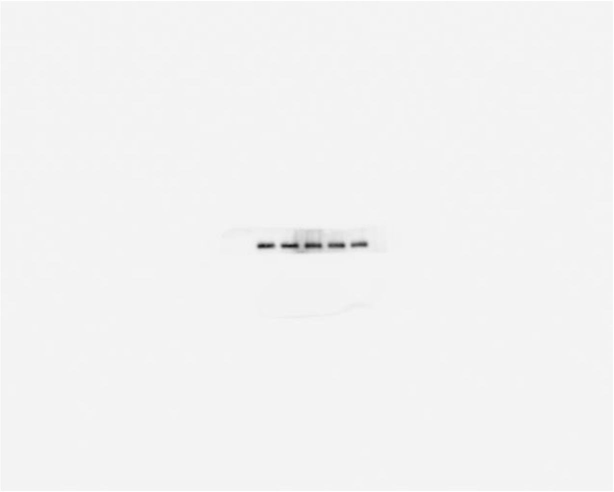

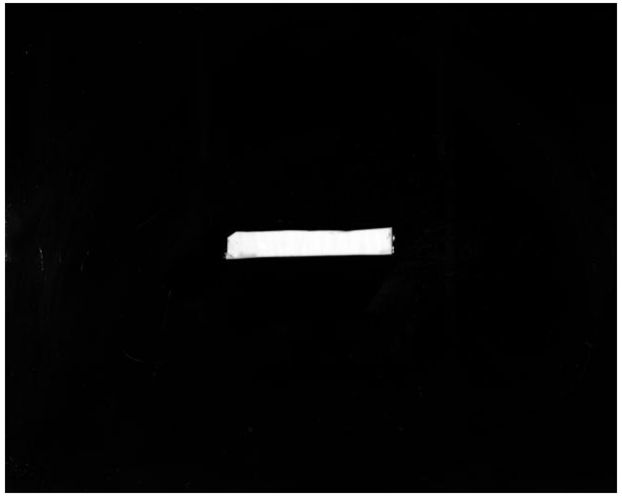

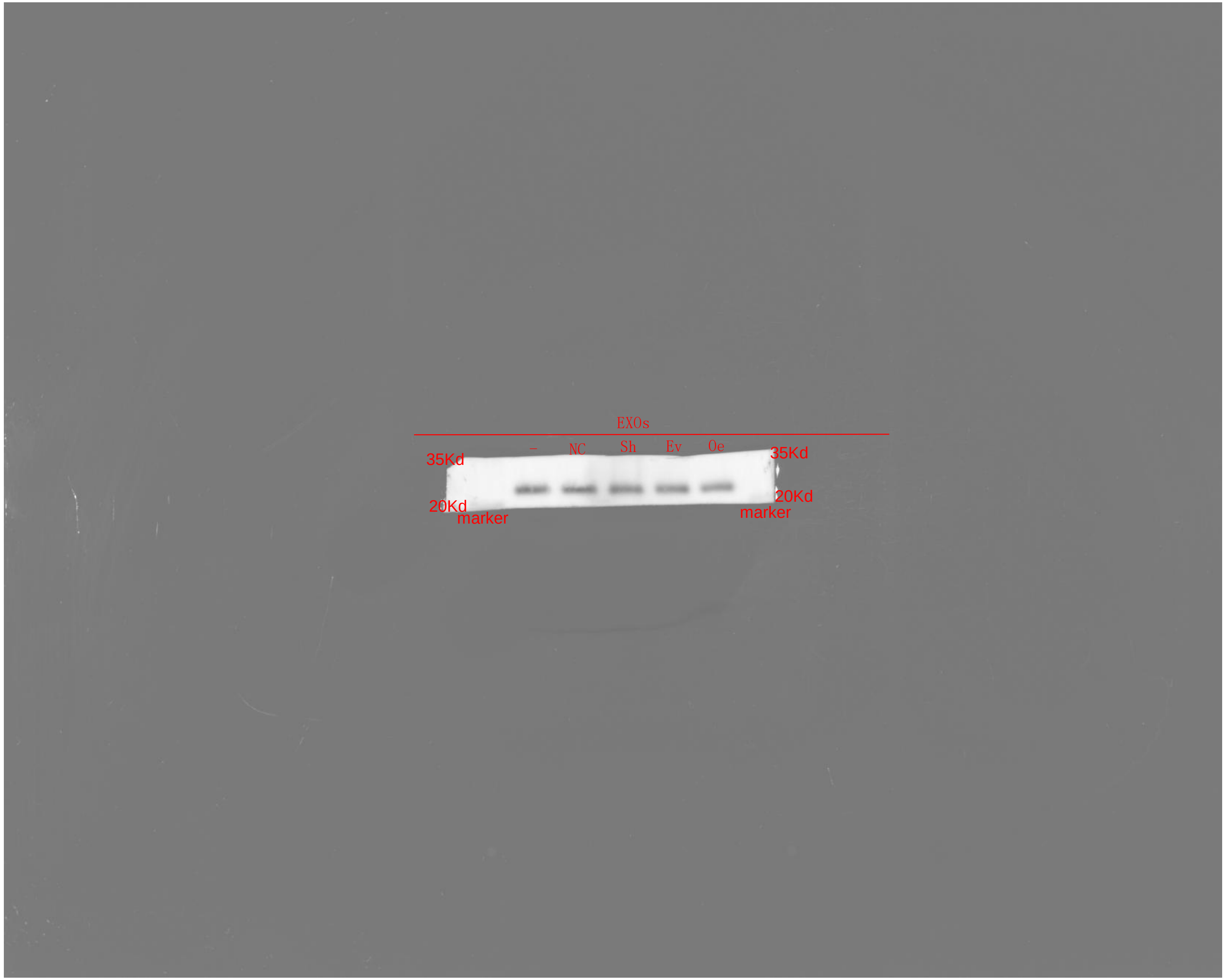

Figure. S6-CD63

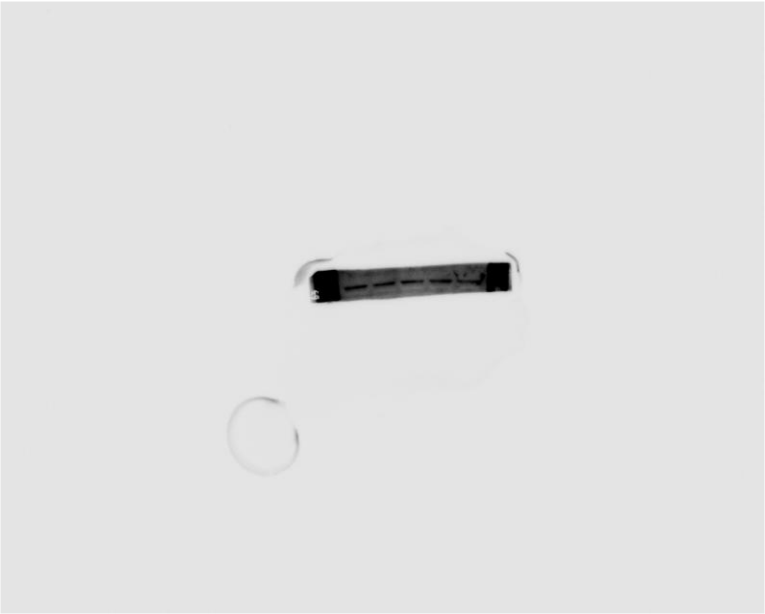

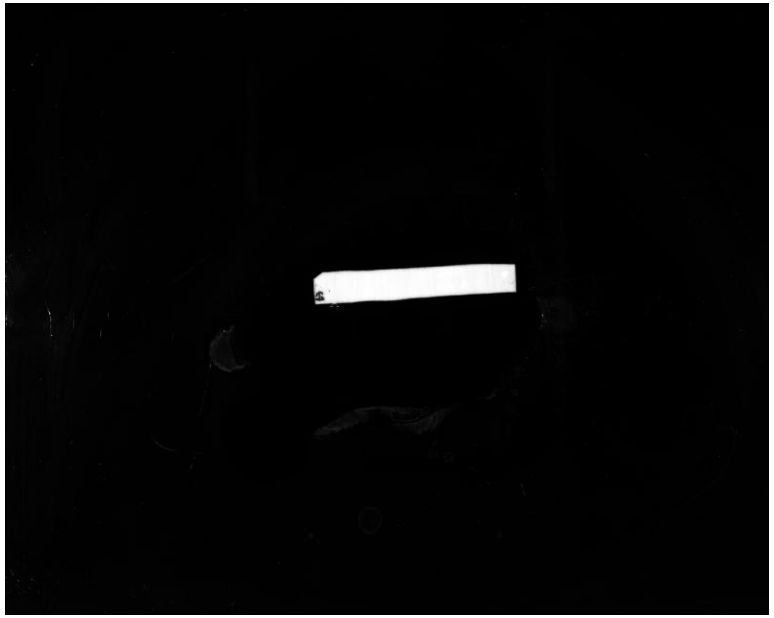

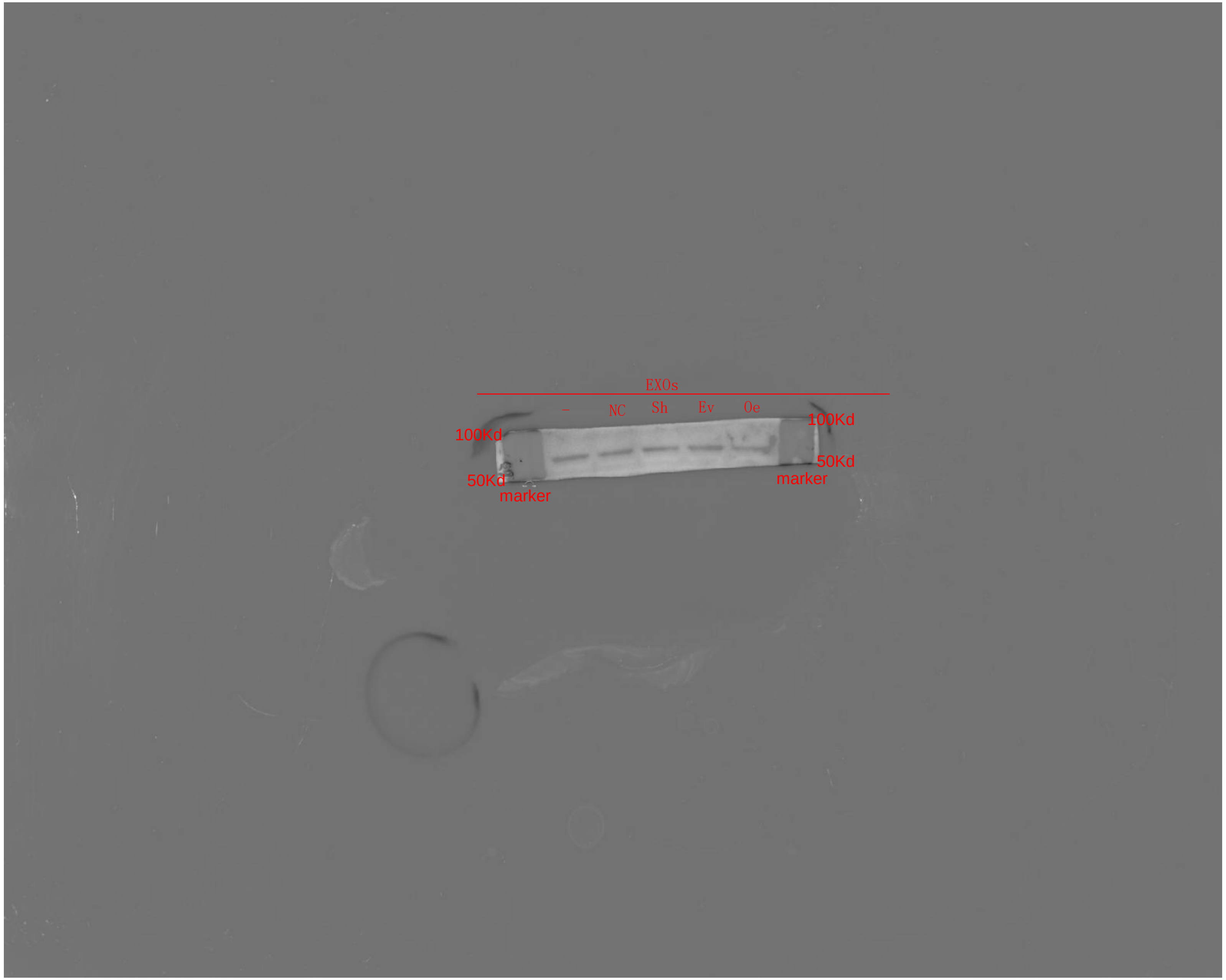

Figure. S6-TSG101

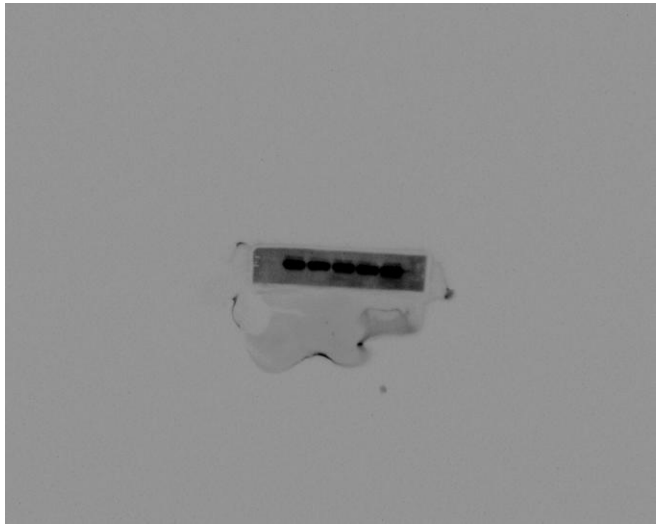

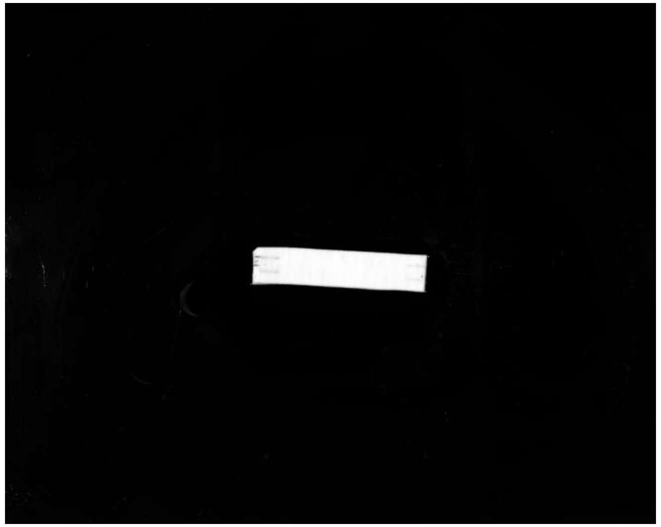

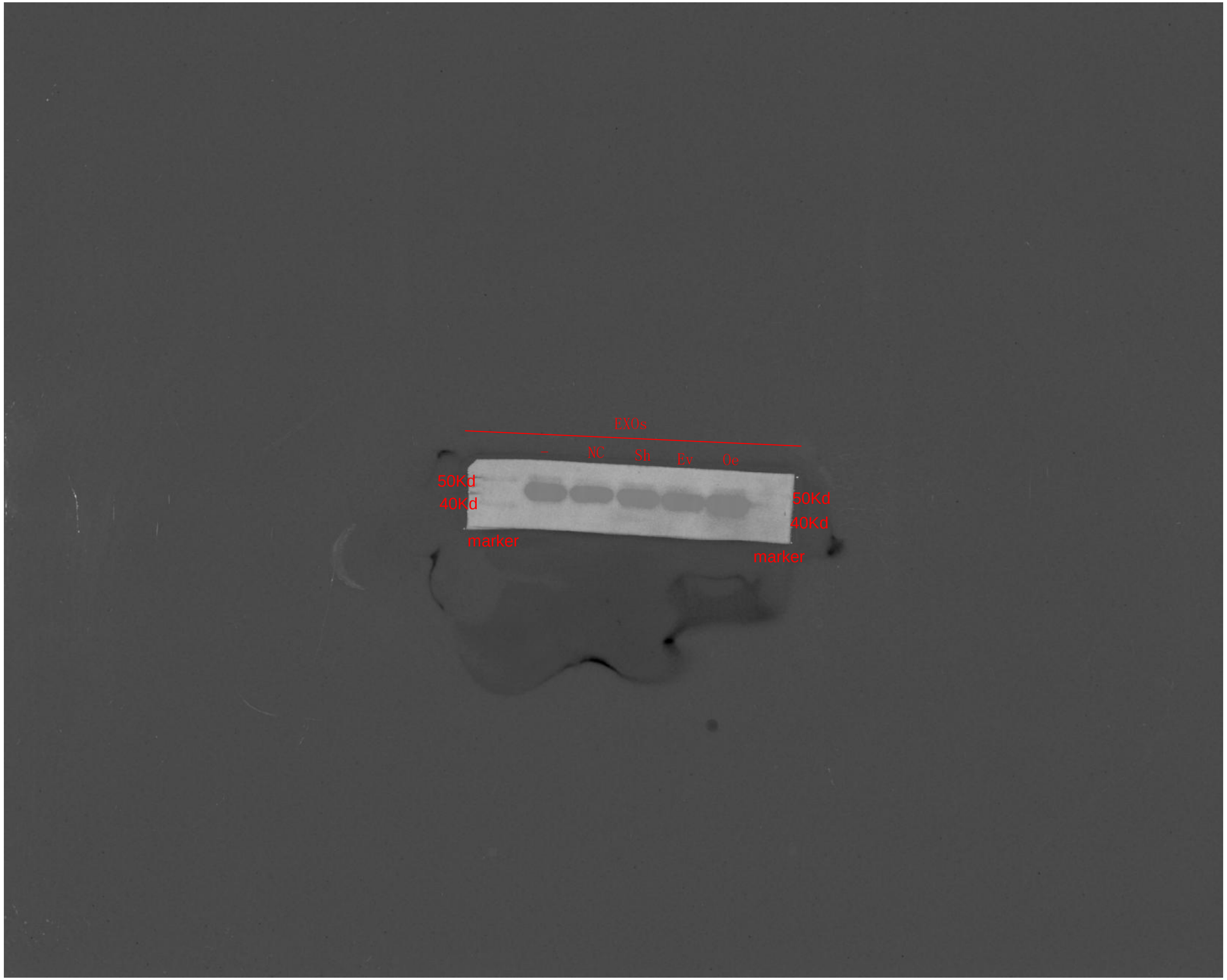

Figure. S7A-p-AKT

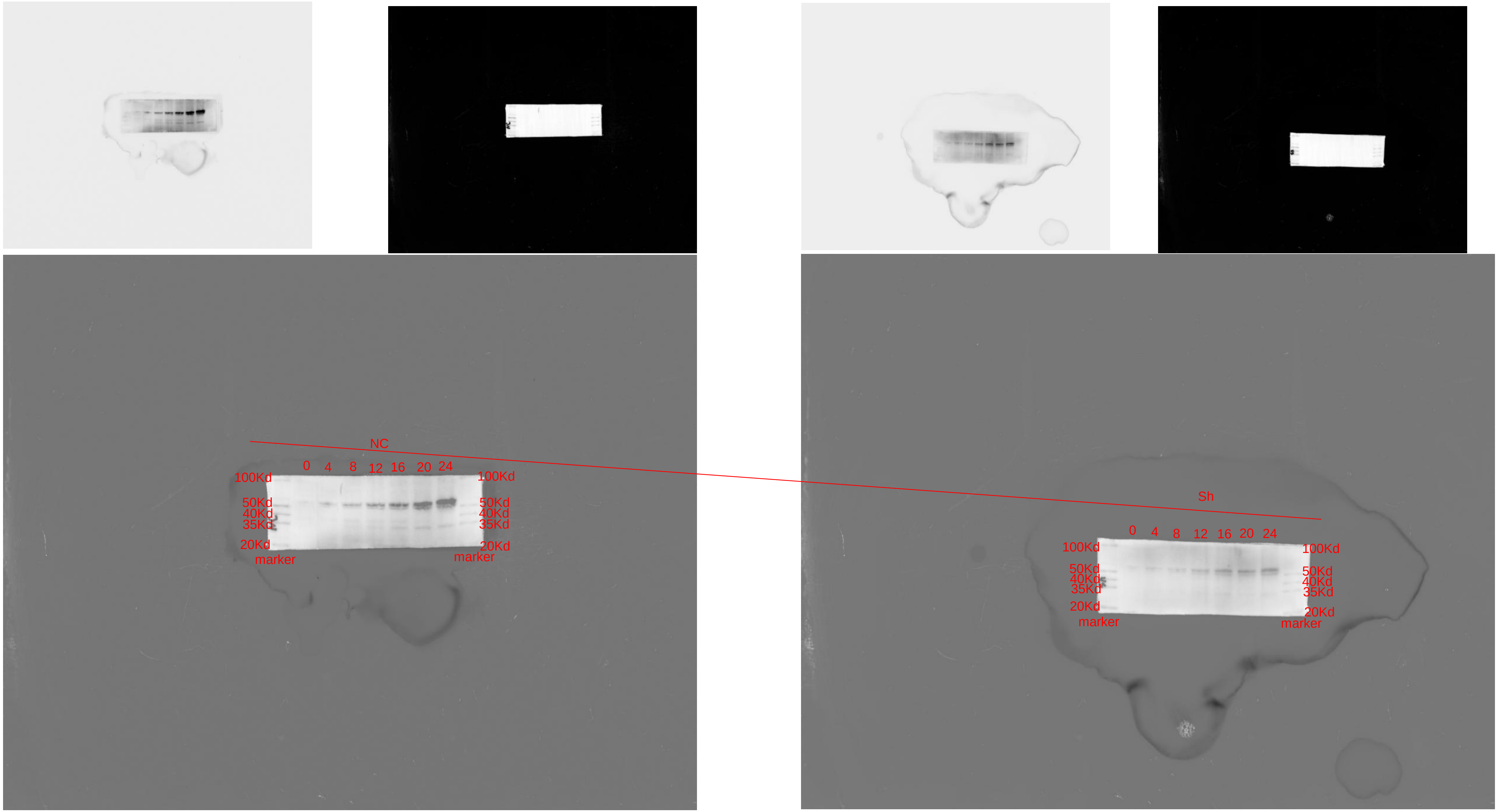

Figure. S7A-AKT

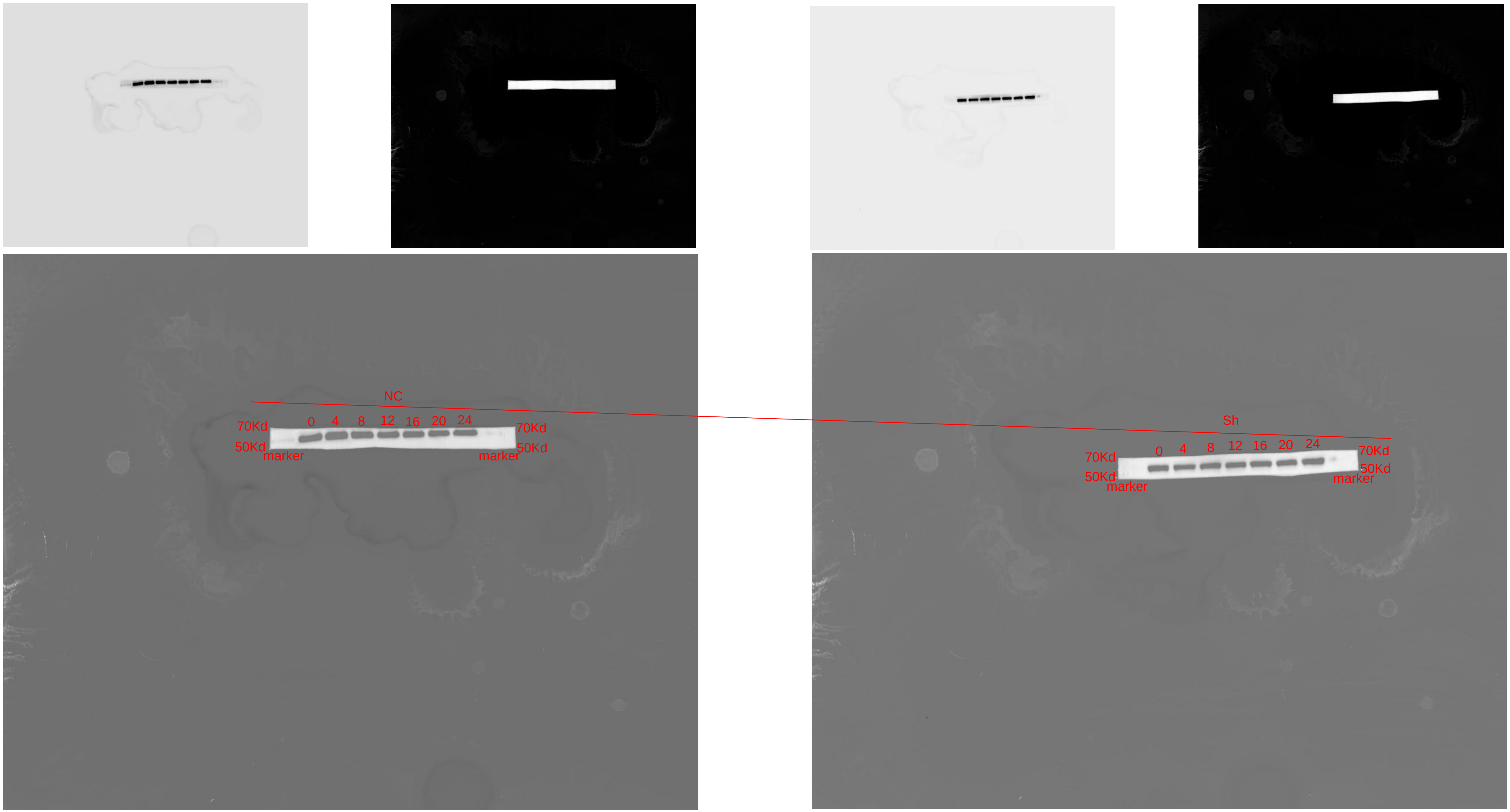

Figure. S7A-p-PI3K

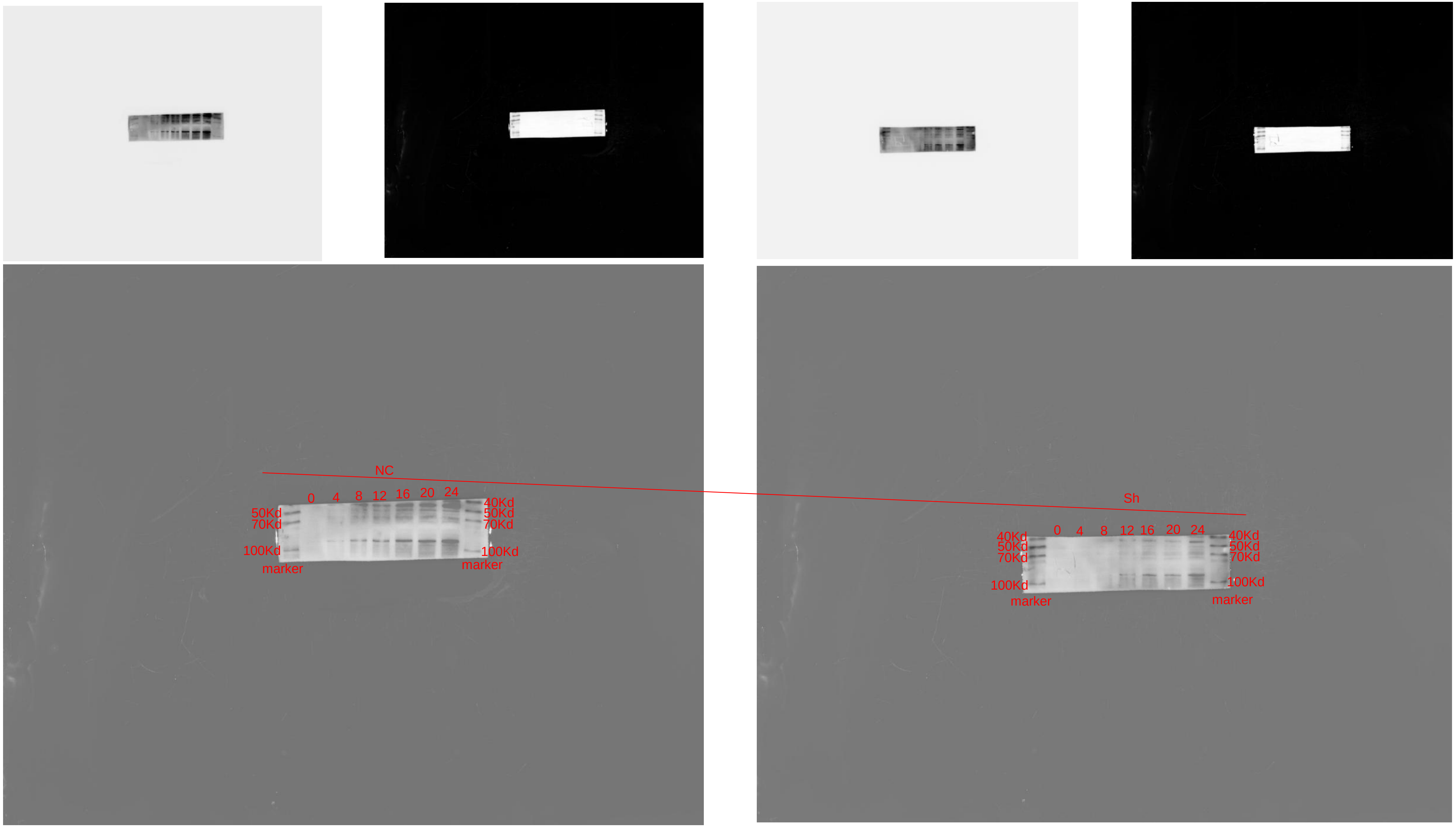

Figure. S7A-PI3K

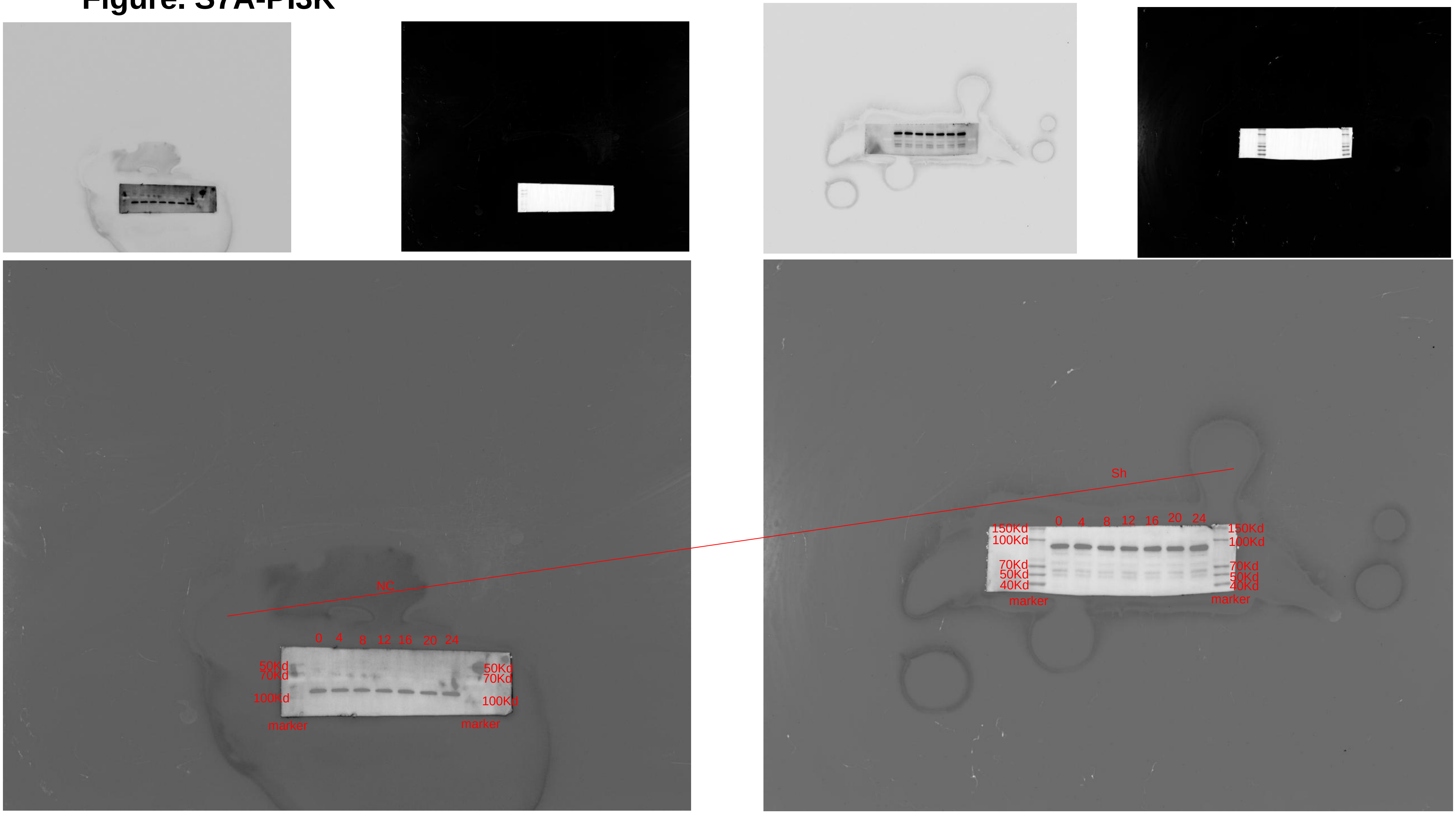

Figure. S7A-β-actin

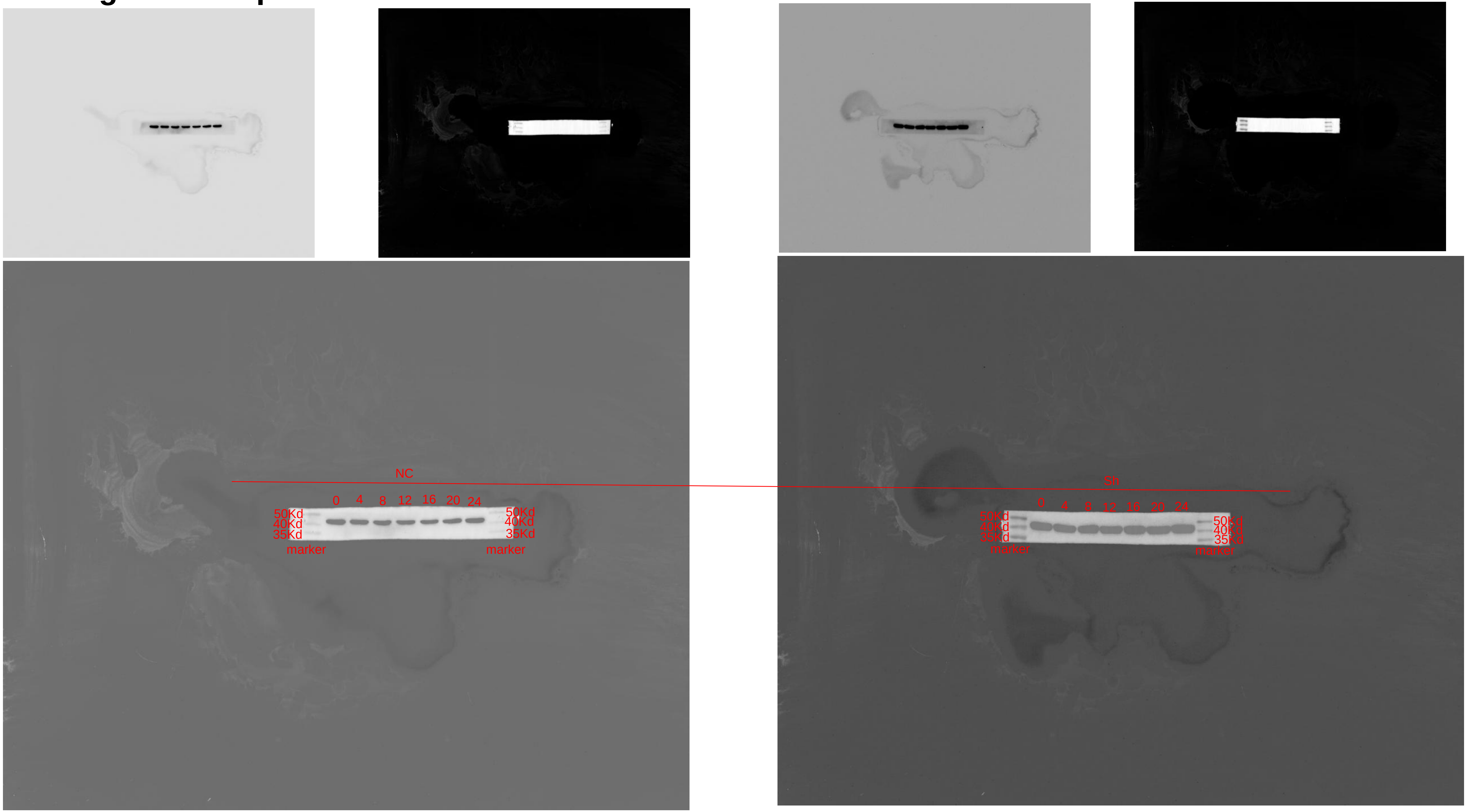

Figure. S7D-p-AKT

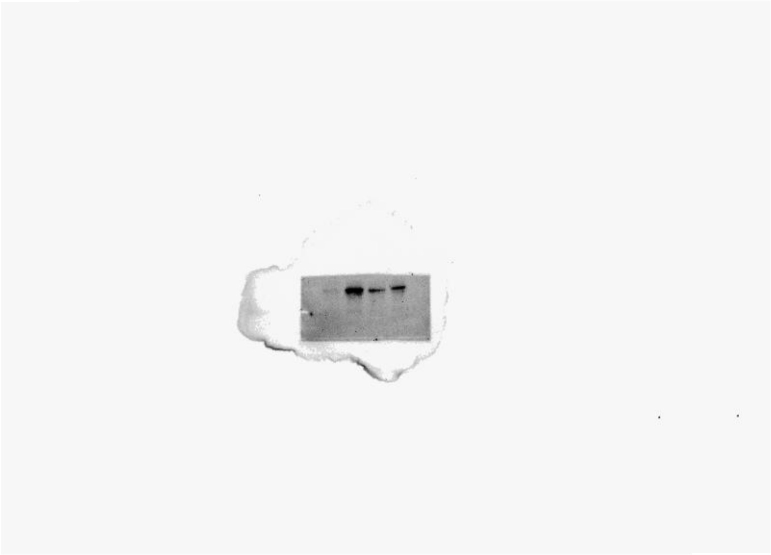

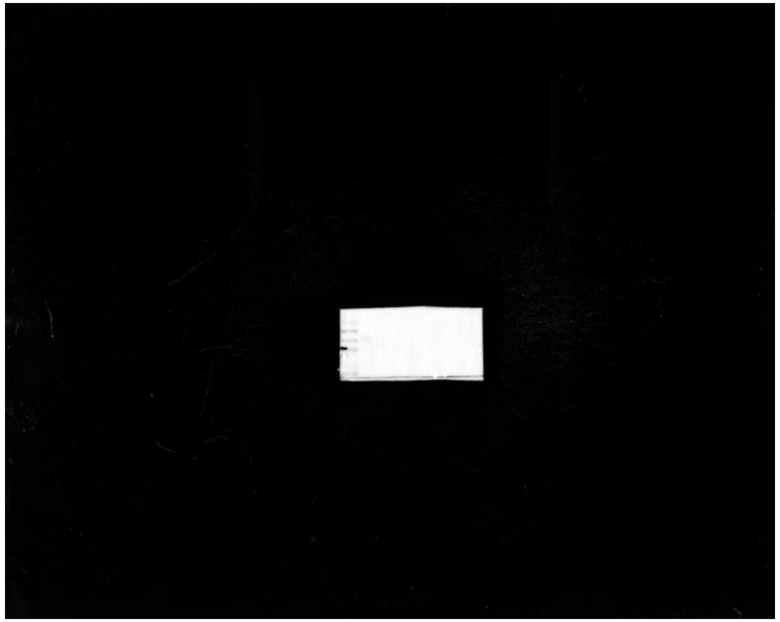

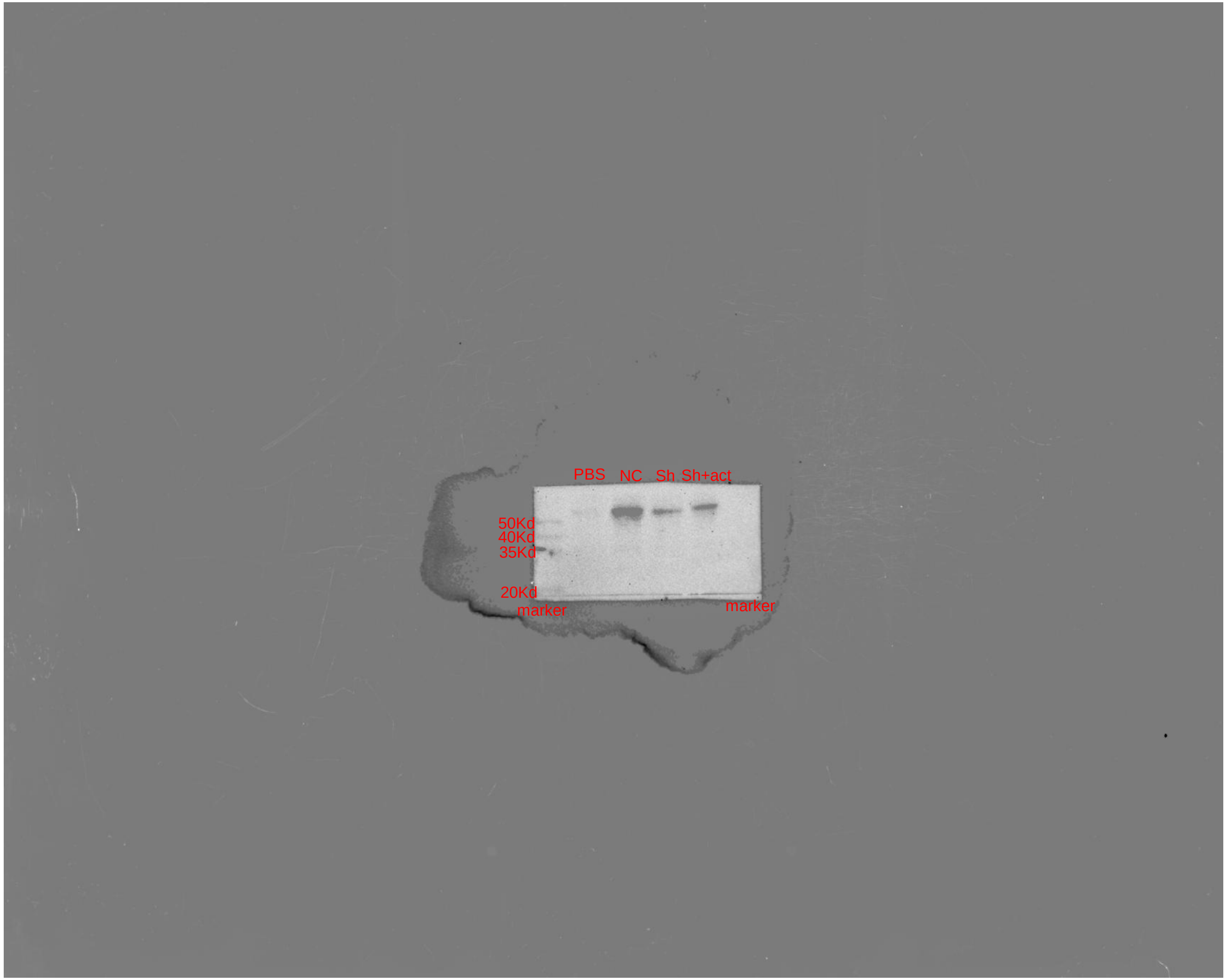

Figure. S7D-AKT

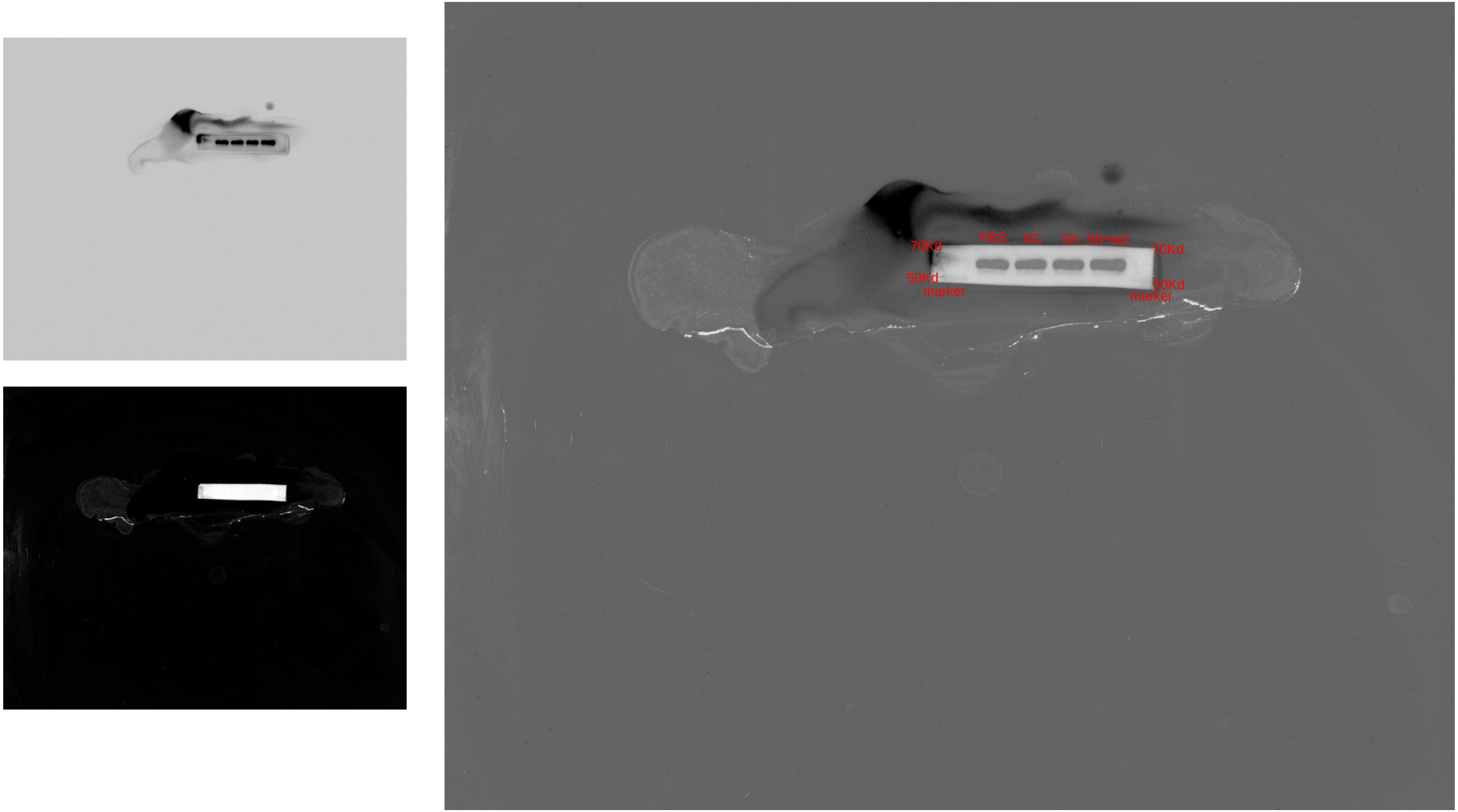

Figure. S7D-β-actin

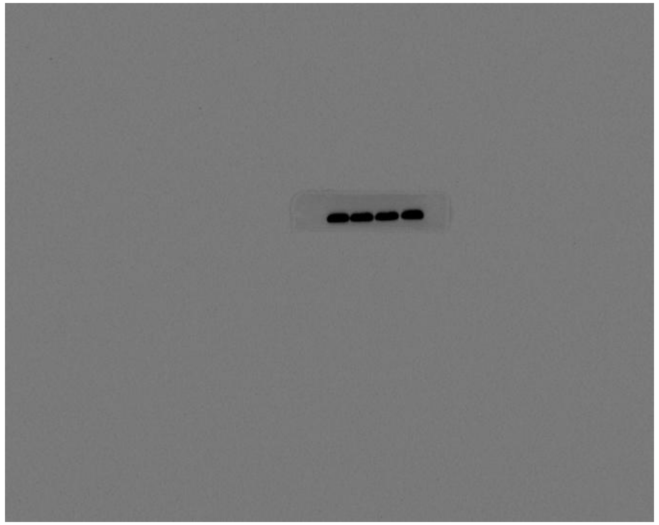

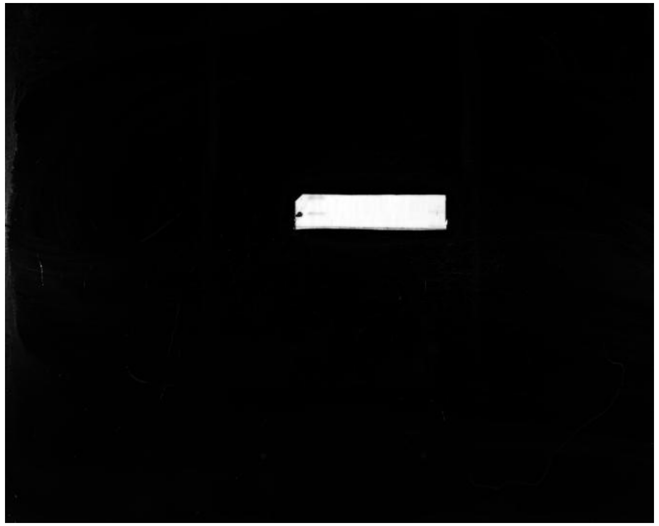

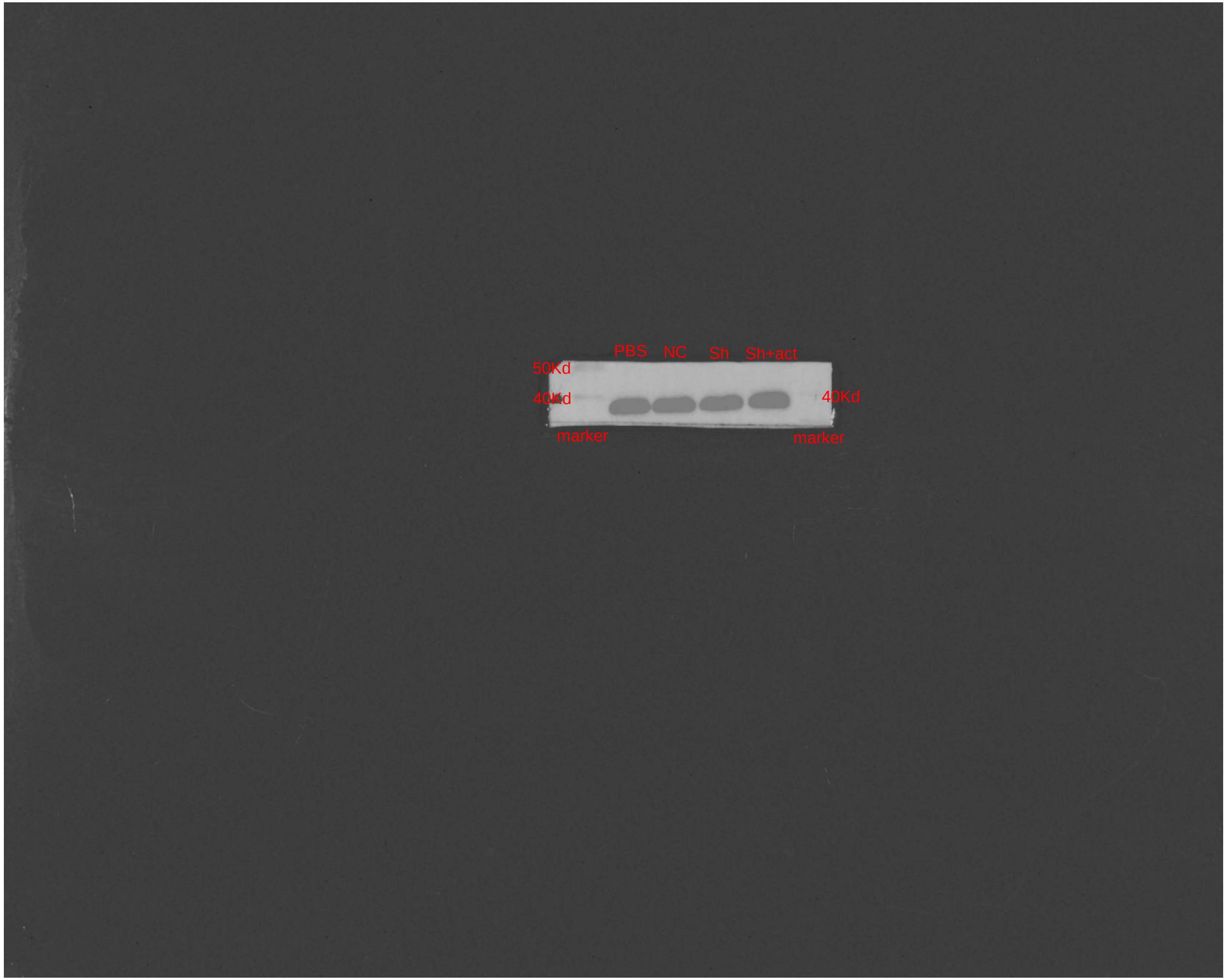

Supplement: Multimedia component 3 [file mmc3.pdf]
